# Preparation of 3-(alkylamino)imidazo[1,2-*a*]pyridine-2-carbaldehydes via Kornblum oxidation and unexpected ring-opening reactions of the corresponding alcohols under oxidative conditions

**DOI:** 10.3762/bjoc.22.58

**Published:** 2026-05-19

**Authors:** Sandile J Mkhize, Memory Zimuwandeyi, Manuel A Fernandes, Amanda L Rousseau, Moira L Bode

**Affiliations:** 1 Molecular Sciences Institute, School of Chemistry, University of the Witwatersrand, Private Bag 3, PO WITS, 2050, South Africa

**Keywords:** 3-aminoimidazo[1,2-*a*]pyridine-2-carbaldehydes, Groebke–Blackburn–Bienaymé reaction, Kornblum oxidation, oxidative ring-opening, reductive amination

## Abstract

The first synthesis of 3-(alkylamino)imidazo[1,2-*a*]pyridine-2-carbaldehydes is reported. A Groebke–Blackburn–Bienaymé reaction between 2-aminopyridine derivatives, cyclohexyl isocyanide and glyoxylic acid in the presence of methanol and an acid catalyst gave the 2-ester derivatives that were reduced to give the corresponding alcohols. Mild Kornblum oxidation conditions, reaction in the presence of DMSO and NaHCO_3_ under conventional or microwave heating to ≈100 °C, were applied to the bromides derived from these alcohols by treatment with PBr_3_, resulting in the desired aldehydes which successfully underwent reductive amination reactions with 2-chloroaniline. Alternative oxidation conditions such as PCC, IBX or *T. versicolor* laccase applied to the alcohols led only to oxidative ring-opening to give oxalamide derivatives, with no aldehyde being isolated.

## Introduction

**Figure 1:**
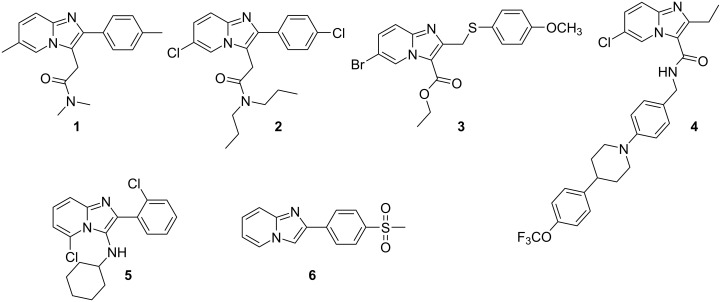
Examples of biologically active imidazo[1,2-*a*]pyridines.

Imidazo[1,2-*a*]pyridines display a wide range of different biological activities, and this scaffold has come to be regarded as being biologically privileged [1]. Compounds containing the imidazo[1,2-*a*]pyridine core have been found to act on targets in the central nervous system. For example, zolpidem (**1**) is used for the treatment of sleeping disorders, while alpidem (**2**), now discontinued due to safety concerns, was used for the treatment of anxiety (Figure 1). Other derivatives are currently under investigation for their CNS-active properties, showing promise for the treatment of conditions such as Parkinson’s disease [2]. Compounds containing the imidazo[1,2-*a*]pyridine core have also shown potential as anti-infective agents, with some compounds showing activity against *Streptococcus pneumoniae* (**3**) [3], tuberculosis (**4**) [4], and HIV (**5**), [5] to name a few (Figure 1). The imidazo[1,2-*a*]pyridine drug zolimidine (**6**) is used for the treatment of gastroesophageal reflux disease [6] while other compounds have exhibited anticancer [7–8] and anti-inflammatory [9–10] activity. More recently, imidazo[1,2-*a*]pyridine derivatives have been found to be of interest in the development of organic materials with interesting properties [11–12].

The importance of imidazo[1,2-*a*]pyridines is evidenced by the plethora of recent review articles covering methods for their preparation [13–16] and functionalisation reactions [17–19].

**Figure 2:**
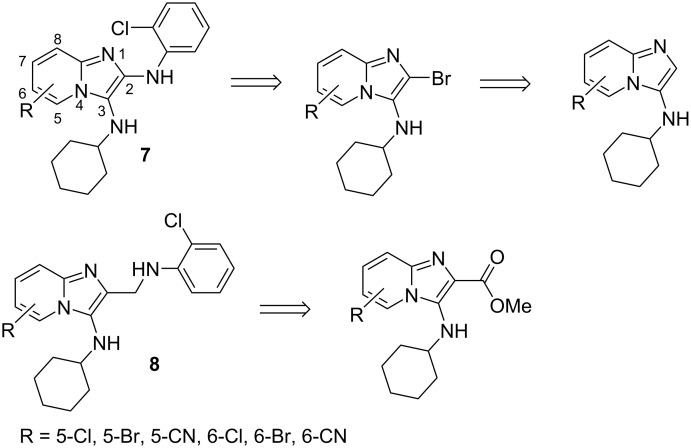
Compounds envisaged for synthesis.

Our own interest in the chemistry of imidazo[1,2-*a*]pyridines arose from the discovery that compounds such as **5** exhibit significant activity against wild-type HIV-1, acting as non-nucleoside reverse transcriptase inhibitors (NNRTIs) [5]. Subsequent investigations revealed that these compounds displayed reduced activity against mutant viral strains, possibly because of a lack of torsional flexibility which could potentially be increased by introduction of a heteroatom between the two aromatic moieties. Thus, we embarked upon this current study to prepare derivatives possessing increased torsional flexibility which might maintain activity against the viral mutants. The plan was to increase compound flexibility through introduction of a bridging nitrogen atom between the imidazopyridine ring and the benzene ring of the original compounds such as **5**. Initially, it was envisaged that chemotype **7** could be accessed from the corresponding 2-bromo derivative as shown in Figure 2 (top). Halogen substituents or a cyano group were planned at positions 5 and 6 of the imidazo[1,2-*a*]pyridine ring and the *ortho*-chloroaniline moiety was envisaged to be appended directly to position 2 because our earlier studies had shown that anti-HIV activity was highly dependent on the presence of these groups [5]. However, during the course of this work, we discovered a superior strategy to the 2-aminomethyl derivatives **8**, as shown in Figure 2 (bottom), which could be easily accessed from the corresponding ester. In this paper, we describe the synthesis of a library of derivatives of **8**, whose imidazo[1,2-*a*]pyridine core could be accessed via the Groebke–Blackburn–Bienayme (GBB) multicomponent coupling reaction [20–23].

## Results and Discussion

**Scheme 1:**
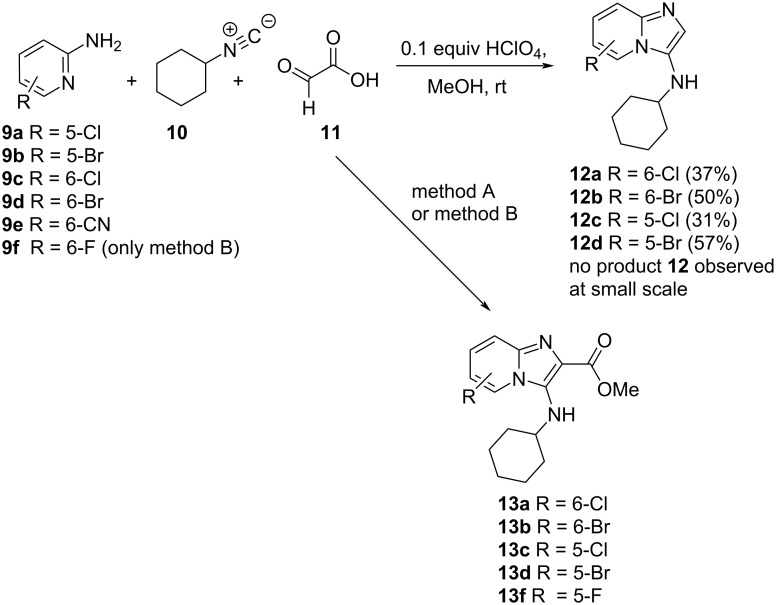
Preparation of methyl esters **13** versus unsubstituted derivatives **12** under various conditions. Method A: 0.1 equiv HClO_4_, MeOH, rt (≈350 mg aminopyridine); method B: 1. glyoxylic acid monohydrate, 0.2 equiv HClO_4_, MeOH, reflux, 2 h; 2. aminopyridine **9**, cyclohexyl isocyanide **10**, rt, 20–24 h.

In order to access our initial target compounds **7**, the GBB reaction was performed using a suitably substituted 2-aminopyridine **9a–e**, cyclohexyl isocyanide (**10**) and glyoxylic acid monohydrate (**11**) in the presence of 0.1 equiv HClO_4_ (relative to aminopyridine) to afford the imidazo[1,2-*a*]pyridin-3-amine product **12** unsubstituted at position 2 (Scheme 1), using conditions described by Gladysz et al. [24]. Reaction with glyoxylic acid as the aldehyde component has previously been reported to yield the 2-unsubstituted product as a result of in situ decarboxylation [25]. Unexpectedly, on product isolation we discovered that compounds **13a**–**d**, the methyl esters, had been obtained from 2-aminopyridines **9a**–**d** while no reaction was observed for 2-aminopyridine **9e** (Scheme 1). We reasoned that at the relatively small scale of the reaction (≈350 mg aminopyridine) and because a period of 30 min stirring of all reactants had been allowed prior to addition of the isocyanide, the glyoxylic acid had undergone in situ esterification in the presence of methanol and the acid catalyst.

On scaling the reaction (≈2 g aminopyridine), the originally anticipated unsubstituted products **12a**–**d** were prepared as the sole products in yields ranging from 31% to 57%. Once again, only starting material was recovered from reaction of compound **9e**. In order to prevent possible mixtures of the two products being formed with slight variation in reaction conditions, the reaction to prepare ester **13** was properly optimised to ensure that full in situ esterification of the glyoxylic acid had taken place before addition of the other reagents. Increasing the amount of acid catalyst to 0.2 equiv significantly increased the yield of the ester products **13**, although the yields were still modest (Table 1). A further increase to 0.3 equiv of acid catalyst did not lead to further yield improvement. Compound **9f** was also successfully converted into **13f** under the conditions shown in Table 1. Nenajdenko et al. reported a few examples of the preparation of imidazo[1,2-*a*]pyridin-3-amine ethyl esters starting from ethyl glyoxylate in toluene and using ammonium chloride as a catalyst in yields of 30–35% [26].

**Table 1:** Yield optimisation using varying amounts of acid catalyst.^a^

Compound	HClO_4_		

	0.1 equiv	0.2 equiv	0.3 equiv

**13a**	26%	41%	43%
**13b**	28%	58%	56%
**13c**	42%	51%	48%
**13d**	44%	55%	56%
**13f**	–	51%	–

^a^Reaction conditions: aminopyridine (1 equiv), cyclohexyl isocyanide (1.1 equiv), glyoxylic acid monohydrate (1.5 equiv), perchloric acid as specified above.

**Scheme 2:**
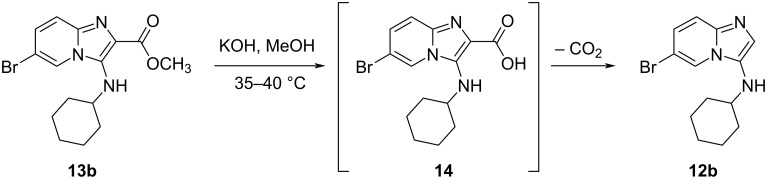
Ester hydrolysis and in situ decarboxylation.

With the relatively facile preparation of esters **13a**–**d** and **13f** now demonstrated, we shifted our focus to investigate possible routes to compounds such as **8**, that have not been reported previously. There were three immediate possibilities to consider: i) reaction of the ester directly with 2-chloroaniline, followed by reduction; ii) reaction of the corresponding carboxylic acid with 2-chloroaniline under peptide-coupling conditions followed by reduction; and iii) reduction of the ester to the aldehyde, followed by reductive amination to give compounds **8**. Initial attempts at direct conversion of ester **13b** into the corresponding amide using AlCl_3_ were not promising and therefore the hydrolysis of this ester to corresponding carboxylic acid **14** was tested. Using KOH in MeOH at 35–40 °C for the ester hydrolysis reaction resulted in decarboxylated compound **12b** being isolated as the sole product in 60% yield (Scheme 2).

**Figure 3:**
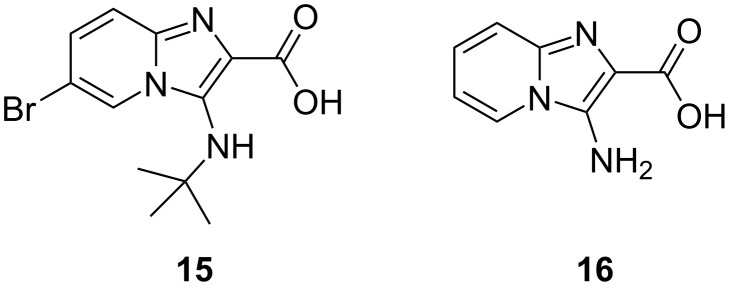
Previously reported 3-amino-2-carboxylic acid derivatives.

Interestingly, there appears to be only one report, in the patent literature [27], of a compound (**15**) containing both 2-carboxylic acid and 3-alkylamino substituents (Figure 3). The unsubstituted 3-aminoimidazo[1,2-*a*]pyridine-2-carboxylic acid (**16**) has also been reported [28]. The very small number of compounds of this type reported is suggestive of their inherent instability and their tendency to readily decarboxylate.

**Scheme 3:**
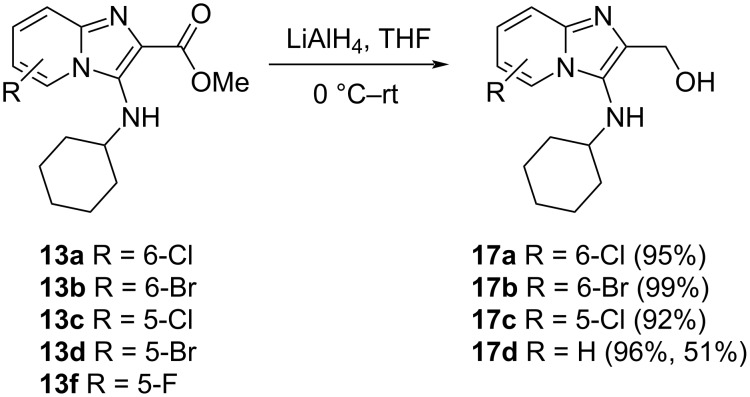
Ester reduction to the corresponding alcohols. Reaction yields are provided in parentheses.

Having ruled out using a peptide-coupling approach we moved to investigate the preparation of the 2-substituted aldehyde derivative, with subsequent reductive amination in mind. Imidazo[1,2-*a*]pyridin-3-amines bearing an aldehyde substituent at the 2-position have not been previously reported and we were uncertain as to their stability. Attempted reduction of the ester (**13b**) to the corresponding aldehyde using DIBAL-H met with failure, with unreacted ester being recovered. Thus, for the preparation of the aldehydes we opted for reduction of the esters **13a**–**d** and **13f** to their corresponding alcohol derivatives (**17**) followed by oxidation to the aldehyde. Esters **13a–c** were readily converted into the corresponding alcohols **17a–c** in excellent yields of 92–99% using LiAlH_4_ reduction (Scheme 3). Unexpectedly, reduction of **13d** and **13f** led to the same compound, the dehalogenated derivative **17d** in 96% and 51% yield, respectively, presumably through nucleophilic aromatic substitution [29].

Oxidation of alcohols **17a**–**d** using pyridinium chlorochromate (PCC) did not lead to the anticipated aldehyde derivatives. Although a cursory inspection of the ^1^H NMR spectrum seemed to indicate that an aldehyde had been formed, with a single proton signal appearing at 9.8 ppm (Figure S24, in Supporting Information File 1), on closer inspection it became clear that there was an additional proton observed in the aromatic region and one missing from the aliphatic region.

**Figure 4:**
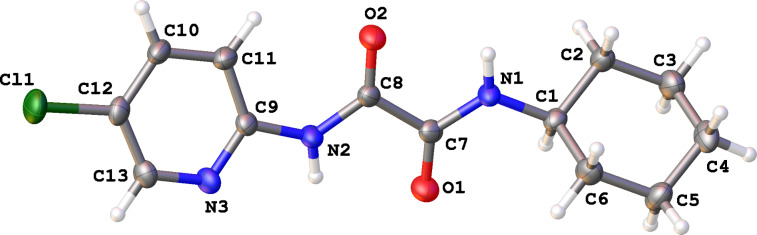
Single crystal X-ray structure of **18a**. ORTEP diagram drawn at 50% probability level.

Crystals were grown for the unexpected oxidation product formed from **17a** and X-ray crystallography revealed that oxidative ring-opening had occurred to give product **18a** (Figure 4).

**Scheme 4:**
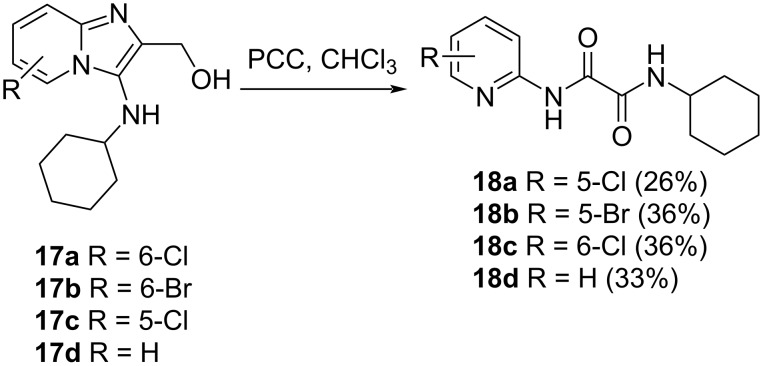
Oxidative ring-opening with loss of one carbon atom. Yields are provided in parentheses.

Carrying out similar reactions on alcohols **17b–d** led to the corresponding ring-opened products **18b**–**d** (Scheme 4). The ring-opened products were isolated in yields of 26–36%, with loss of one carbon atom occurring (-CH_2_OH).

**Scheme 5:**
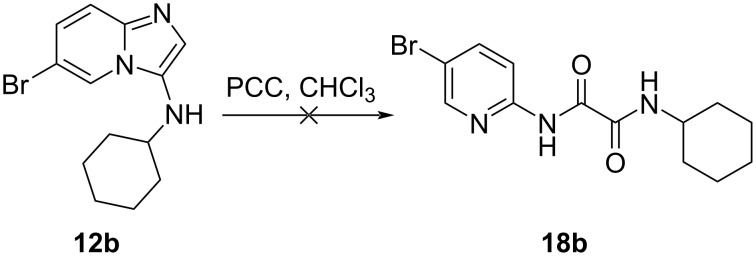
Oxidative conditions applied to decarboxylated compound **12b**.

To test whether C-2 decarboxylation could have immediately preceded ring-opening, decarboxylated compound **12b** was subjected to the same oxidative conditions applied to **17** and the reaction mixture was monitored for any sign of compound **18b** (Scheme 5). Only starting material was isolated from the reaction, indicating that it is unlikely that decarboxylation is the first step of the ring-opening mechanism.

**Figure 5:**
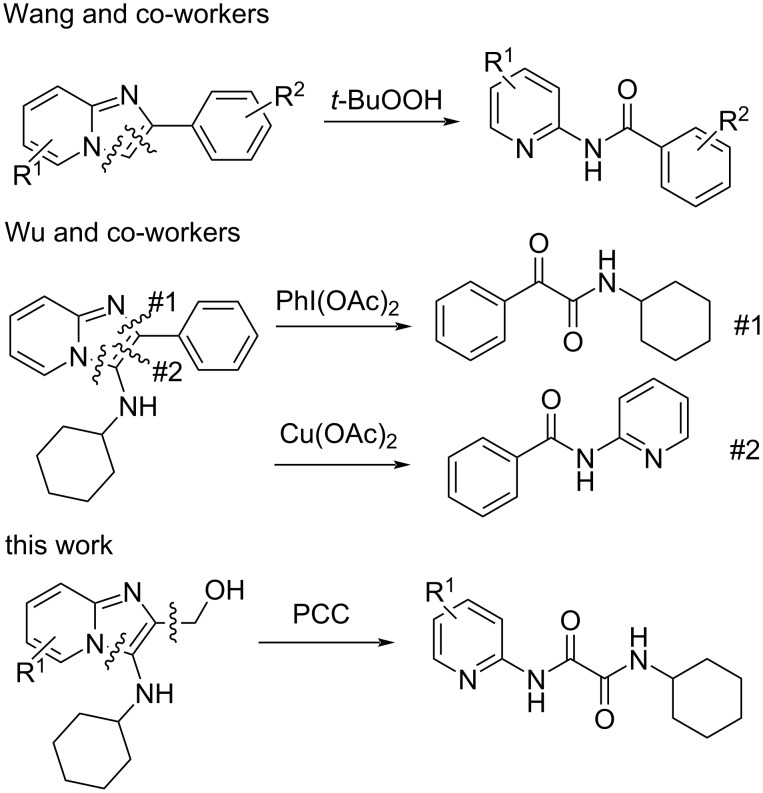
Different oxidative cleavage products obtained under different conditions.

Oxidative ring-opening of imidazo[1,2-*a*]pyridines has been reported previously, but in these cases different ring-opened products were identified. These previous results are compared to our present report in Figure 5. Wang and co-workers [30] reported ring-opening accompanied by loss of a carbon atom, giving rise to benzamides, while Wu and co-workers [31] reported ring-opening to give either benzamides or α-ketoamides. This is in contrast to our own report, where only the oxalamide derivatives **18** were isolated.

**Scheme 6:**
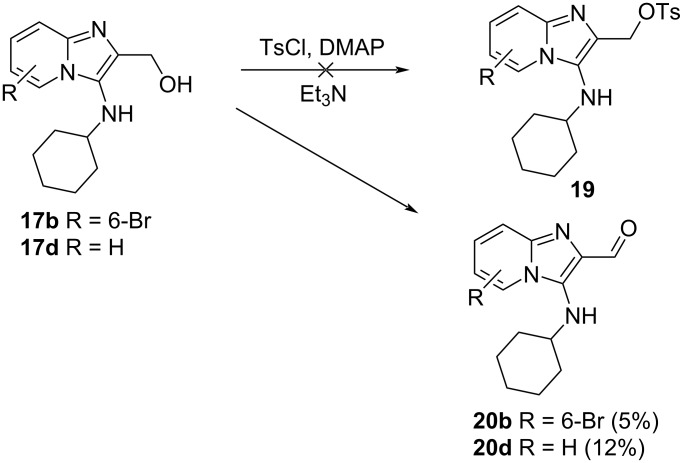
Unexpected aldehyde formation from tosylation reaction.

Two other oxidation methods, IBX and enzymatic oxidation using *T. versicolor* laccase in the presence of TEMPO and O_2_, were tested on compounds **17** in order to obtain the corresponding aldehydes, but both of these methods also gave rise to the ring-opened products **18**. A detailed investigation into the mechanism of this ring-opening reaction was not conducted. However, this failure to obtain the desired aldehydes led to a modification of the planned synthesis. A decision was made to convert alcohols **17** into the corresponding tosylate derivatives **19** (Scheme 6) and then react these with anilines to obtain the desired products **8**. To our complete surprise, reaction of **17b** and **17d** under typical tosylation conditions gave none of the anticipated tosylate derivatives **19** but instead gave small quantities of the elusive aldehydes **20b** (5%) and **20d** (12%), together with unreacted starting material. This result showed that these aldehydes are indeed stable and isolable, once prepared. 3-(Alkylamino)imidazo[1,2-*a*]pyridine-2-carbaldehydes have not been previously reported, but there is a single report of the *N*-unsubstituted 2-formyl-3-aminoimidazo[1,2-*a*]pyridine, prepared from the corresponding nitro-derivative [32].

**Scheme 7:**
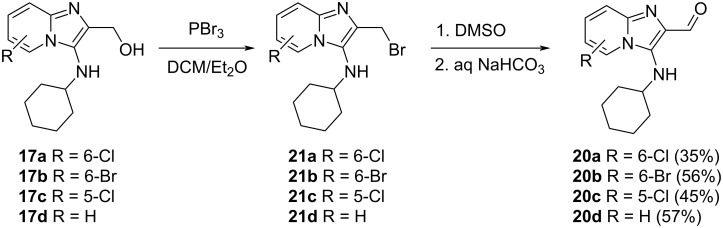
Kornblum oxidation to give imidazo[1,2-*a*]pyridine-2-carbaldehydes **20**. Yield over two steps from the alcohol is shown in parentheses. Compound **20d** was used crude in the reductive amination reaction.

The conversion of tosylates (or halides) into their corresponding aldehydes, which might have occurred here, can be achieved by means of the Kornblum oxidation [33] and thus our attention turned to the very mild oxidation conditions of this reaction and we did not explore the tosylation reaction further. Conversion of the alcohols **17a**–**d** into bromides **21a**–**d** was successfully achieved using PBr_3_ and the bromides were further reacted in situ, due to their highly hygroscopic nature, into the corresponding aldehydes **20a**–**d** using Kornblum conditions (Scheme 7). The reaction could be carried out under conventional heating or using microwave irradiation.

**Scheme 8:**
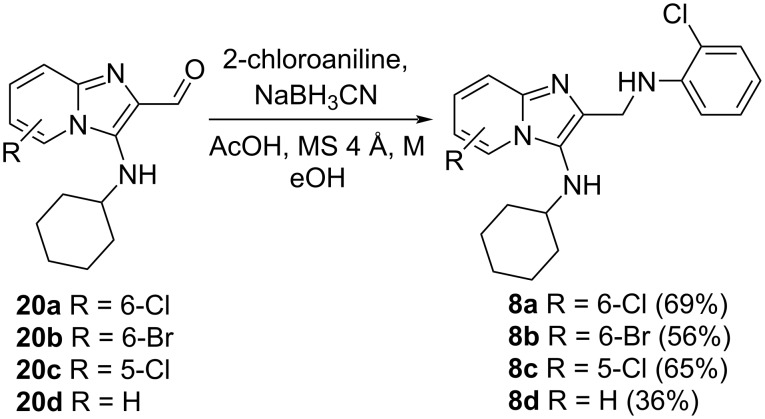
Reductive amination reactions to give target molecules **8**.

The aldehydes **20a**–**d** were successfully subjected to reductive amination conditions to give the desired amine products **8a**–**d** (Scheme 8). To the best of our knowledge, this is the first reported synthesis of aldehydes **20** and products of reductive amination, such as **8**.

## Conclusion

A method for the preparation of 3-(alkylamino)imidazo[1,2-*a*]pyridine-2-carbaldehydes has been developed that proceeds via the 2-methyl esters resulting from the Groebke–Blackburn–Bienaymé reaction, followed by reduction to the corresponding alcohols, bromination and then mild Kornblum oxidation. Other typical oxidation reagents such as PCC, IBX and laccase gave only a ring-opened oxalamide product, resulting from oxidative ring-opening. Aldehydes prepared were subjected to reaction with 2-chloroaniline in the presence of sodium cyanoborohydride to give the corresponding reductive amination products which will be tested for activity against HIV-1 reverse transcriptase in due course.

## Supporting Information

File 1 Experimental procedures, copies of NMR spectra and X-ray data of compound **18a**.

## Data Availability

All data that supports the findings of this study is available in the published article and/or the supporting information of this article. CCDC deposition number 2533649 contains the supplementary crystallographic data for this paper.

## References

[1] Devi N, Rawal R K, Singh V (2015). Tetrahedron.

[2] Vanda D, Zajdel P, Soural M (2019). Eur J Med Chem.

[3] Jahan K, Battaje R R, Pratap V, Ahire G, Pushpakaran A, Ashtam A, Bharatam P V, Panda D (2024). Eur J Med Chem.

[4] Samanta S, Kumar S, Aratikatla E K, Ghorpade S R, Singh V (2023). RSC Med Chem.

[5] Bode M L, Gravestock D, Moleele S S, van der Westhuyzen C W, Pelly S C, Steenkamp P A, Hoppe H C, Khan T, Nkabinde L A (2011). Bioorg Med Chem.

[6] Prasher P, Sharma M (2024). Chem Pap.

[7] Dam J, Ismail Z, Kurebwa T, Gangat N, Harmse L, Marques H M, Lemmerer A, Bode M L, de Koning C B (2017). Eur J Med Chem.

[8] An W, Wang W, Yu T, Zhang Y, Miao Z, Meng T, Shen J (2016). Eur J Med Chem.

[9] Hieke M, Rödl C B, Wisniewska J M, la Buscató E, Stark H, Schubert-Zsilavecz M, Steinhilber D, Hofmann B, Proschak E (2012). Bioorg Med Chem Lett.

[10] Kraft F B, Enns J, Honin I, Engelhardt J, Schöler A, Smith S T, Meiler J, Schäker-Hübner L, Weindl G, Hansen F K (2024). Bioorg Chem.

[11] Zucolotto Cocca L H, Valverde J V P, le Bescont J, Breton-Patient C, Piguel S, Silva D L, Mendonca C R, De Boni L (2024). J Mol Struct.

[12] Bedard N, Coen A G, Pekarske S, Sennett A, Davis G J, Chavez T, Lichtenberger D L, Hulme C (2023). Tetrahedron Lett.

[13] Panda J, Raiguru B P, Mishra M, Mohapatra S, Nayak S (2022). ChemistrySelect.

[14] Kurteva V (2021). ACS Omega.

[15] Tali J A, Kumar G, Sharma B K, Rasool Y, Sharma Y, Shankar R (2023). Org Biomol Chem.

[16] Bhatt K, Patel D, Rathod M, Patel A, Shah D (2022). Curr Org Chem.

[17] Konwar D, Bora U (2021). ChemistrySelect.

[18] Tashrifi Z, Mohammadi‐Khanaposhtani M, Larijani B, Mahdavi M (2020). Eur J Org Chem.

[19] Sofi F A, Gogde K, Mukherjee D, Masoodi M H (2024). J Mol Struct.

[20] Groebke K, Weber L, Mehlin F (1998). Synlett.

[21] Blackburn C, Guan B, Fleming P, Shiosaki K, Tsai S (1998). Tetrahedron Lett.

[22] Bienaymé H, Bouzid K (1998). Angew Chem, Int Ed.

[23] Martini C, Mardjan M I D, Basso A (2024). Beilstein J Org Chem.

[24] Gladysz R, Adriaenssens Y, De Winter H, Joossens J, Lambeir A-M, Augustyns K, Van der Veken P (2015). J Med Chem.

[25] Lyon M A, Kercher T S (2004). Org Lett.

[26] Nenajdenko V G, Reznichenko A L, Balenkova E S (2007). Russ Chem Bull.

[27] Lee K C, Sun E T, Wang H (2008). Imidazo[1,2-a]pyridine derivatives: preparation and pharmaceutical applications. U.S. Pat. Appl..

[28] Márquez-Flores Y K, Campos-Aldrete M E, Salgado-Zamora H, Correa-Basurto J, Meléndez-Camargo M E (2012). Med Chem Res.

[29] Changunda C R K, Venkatesh B C, Mokone W K, Rousseau A L, Brady D, Fernandes M A, Bode M L (2020). RSC Adv.

[30] Yan K, Yang D, Wei W, Li G, Sun M, Zhang Q, Tian L, Wang H (2015). RSC Adv.

[31] Xu F, Wang Y, Xun X, Huang Y, Jin Z, Song B, Wu J (2019). J Org Chem.

[32] Desbois N, Chezal J-M, Fauvelle F, Debouzy J-C, Lartigue C, Gueiffier A, Blache Y, Moreau E, Madelmont J-C, Chavignon O (2005). Heterocycles.

[33] Kornblum N, Jones W J, Anderson G J (1959). J Am Chem Soc.

