# Supplementary material for: Preparation of 3-(alkylamino)imidazo[1,2-a]pyridine-2-carbaldehydes via Kornblum oxidation and unexpected ring-opening reactions of the corresponding alcohols under oxidative conditions
Source: Beilstein J Org Chem. 2026 May 19;22:763–70. doi: 10.3762/bjoc.22.58 (PMC13202479; doi:10.3762/bjoc.22.58)
Supplement: File 1 — Experimental procedures, copies of NMR spectra and X-ray data of compound 18a. [file Beilstein_J_Org_Chem-22-763-s001.pdf]

BEILSTEIN JOURNAL OF ORGANIC CHEMISTRY

Supporting Information

for

Preparation of 3-(alkylamino)imidazo[1,2-*a*]pyridine-2-carbaldehydes via Kornblum oxidation and unexpected ring-opening reactions of the corresponding alcohols under oxidative conditions

Sandile J. Mkhize, Memory Zimuwandeyi, Manuel A. Fernandes, Amanda L. Rousseau and Moira L. Bode

Beilstein J. Org. Chem. **2026,** *22,* 763–770. doi:10.3762/bjoc.22.58

Experimental procedures, copies of NMR spectra and X-ray data of compound 18a

License and Terms: This is a supporting information file under the terms of the Creative Commons Attribution License (https://creativecommons.org/licenses/by/4.0). Please note that the reuse, redistribution and reproduction in particular requires that the author(s) and source are credited and that individual graphics may be subject to special legal provisions.
The license is subject to the *Beilstein Journal of Organic Chemistry* terms and conditions: (https://www.beilstein-journals.org/bjoc/terms)

Table of Contents

Experimental procedures for compounds **8**, **12**,**13**, **17**, **18**, **20** S2

Appendix one: ^{1}H and ^{13}C NMR spectra for all synthesised compounds S25

Single crystal X-ray structure data for compound **18a** S72

Synthesis of imidazo[1,2-*a*]pyridine-2-carboxylate derivatives 13

General procedure:

Glyoxylic acid monohydrate **11** (1.5 equiv) was reacted with excess methanol under reflux for 2 h, in the presence of 70% perchloric acid (20 mol %). The reaction was allowed to cool to room temperature and thereafter an appropriate aminopyridine **9** (1.104 mmol) was added to the mixture and the reaction was left stirring at room temperature for 45 minutes over oven dried molecular sieves. The isocyanide **10** (1.1 equiv) was then introduced slowly over a period of 10–15 minutes and the reaction was stirred at ambient temperature until completion, usually 20–24 h. The solvent was removed under reduced pressure and the concentrate was purified by column chromatography using an appropriate solvent system.

Methyl 6-chloro-3-(cyclohexylamino)imidazo[1,2-*a*]pyridine-2-carboxylate (13a)

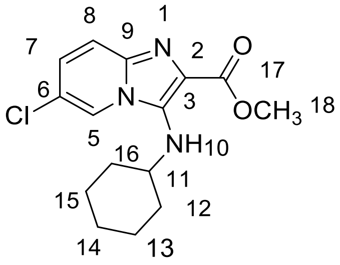

Glyoxylic acid monohydrate (**11**, 890 mg, 11.67 mmol), perchloric acid (1.16 mmol, 100 µL), 2-amino-5-chloropyridine (**9a**, 1.00 g, 7.78 mmol) and cyclohexyl isocyanide (**10**, 1.06 mL, 8.56 mmol) were reacted in methanol (12 mL) to afford compound **13a** as a light-yellow powder (983 mg, 41%). M.p.= 177-179 °C. R_f =0.30 (60% Hex/EtOAC). ^{1}H NMR (400 MHz, $CDCl_3$) δ 7.94 (s, 1H, H-5), 7.47 (d, 1H, *J* = 9.7 Hz, H-8), 7.09 (dd, 1H, *J* = 9.7, 1.9 Hz, H-7), 4.91 (d, 1H, *J* = 12 Hz, N-H), 3.97 (s, 3H, H-18), 3.18-3.04 (m, 1H, H-11), 1.98-1.85 (m, 2H, H-12a & H-16a), 1.82-1.70 (m, 2H, H-13a & H-15a), 1.66-1.54 (m, 1H, H-14a), 1.40-1.16 (m, 5H, H-12b, H-13b, H-14b, H-15b & H-16b); ^{13}C NMR (101 MHz, $CDCl_3$) δ 165.1 (C-17), 138.7 (C-3), 137.1 (C-9), 126.6 (C-7), 123.1 (C-6), 121.2 (C-2), 121.0 (C-5), 119.8 (C-8), 55.7 (C-11), 51.8 (C-18), 34.1 (C-12 & C-16), 25.5 (C-14), 24.8 (C-13 & C-15). IR ν_{max}(cm^{-1}) 3312 (N-H), 1714 (C=O), 705 (C-Cl). HRMS (ES^+) calculated for $C_{15}H_{19}N_3ClO_2$ $[M+H]^+$: 308.1161, found: 308.1176.

Methyl 6-bromo-3-(cyclohexylamino)imidazo[1,2-*a*]pyridine-2-carboxylate (13b)

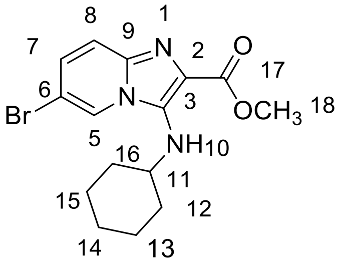

Glyoxylic acid monohydrate (**11**, 660 mg, 8.68 mmol), perchloric acid (1.16 mmol, 100 µL), 2-amino-5-bromopyridine (**9b**, 1.00 g, 5.78 mmol) and cyclohexyl isocyanide (**10**, 6.54 mmol, 790 µL) were reacted in methanol (12 mL) for 24 h to afford **13b** as a dark yellow powder (1.18 g, 58 %). M.p.= 178-180 °C. R_f =0.35 (60% Hex/EtOAC). ^{1}H NMR (400 MHz, $CDCl_3$) δ 8.04 (s, 1H, H-5), 7.41 (d, 1H, *J* = 9.6 Hz, H-8), 7.18 (dd, 1H, *J* = 9.7, 1.8 Hz, H7), 4.91 (d, 1H, N-H), 3.97 (s, 3H, H-18), 3.17-3.05 (m, 1H, H-11), 1.97-1.88 (m, 2H, H-12a & H-16a), 1.85-1.71 (m, 2H, H-13a & H-15a), 1.65-1.56 (m, 1H, H-14a), 1.40-1.18 (m, 5H, H-12b, H-13b, H-14b, H-15b, H-16b); ^{13}C NMR (101 MHz, $CDCl_3$) δ 165.0 (C-17), 138.7 (C-3), 136.9 (C-9), 128.5 (C-7), 123.3 (C-5), 122.9 (C-2), 119.9 (C-8), 107.8 (C-6), 55.7 (C-11), 51.8 (C-18), 34.1 (C-12 & C-16), 25.5 (C-14), 24.8 (C-13 & C-15). IR ν_{max}(cm^{-1}) 3305 (N-H), 1713 (C=O), 666 (C-Br). HRMS (ES$^+$) calculated for $C_{15}H_{19}N_3{}^{79}BrO_2$ $[M+H]^+$: 352.0656, found: 352.0636.

Methyl 5-chloro-3-(cyclohexylamino)imidazo[1,2-*a*]pyridine-2-carboxylate (13c)

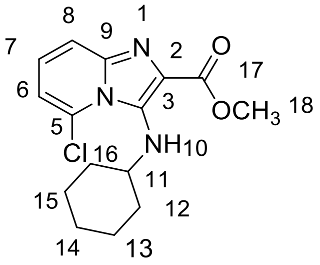

Glyoxylic acid monohydrate (**11**, 594 mg, 7.82 mmol), perchloric acid (1.04 mmol, 90 µL), 2-amino-6-chloropyridine (**9c**, 670 mg, 5.21 mmol) and cyclohexyl isocyanide (**10**, 713 µL, 5.73 mmol) were reacted in methanol (12 mL) to afford compound **13c** as a light-yellow powder (831 mg, 51%). M.p.= 127 °C. R_f =0.30 (60% Hex/EtOAC). ^{1}H NMR (400 MHz, $CDCl_3$) δ 7.46 (dd, 1H, *J* = 9.1, 1.1 Hz, H-8), 7.03 (dd, 1H, *J* = 9.1, 7.1 Hz, H-7), 6.77 (dd, 1H, *J* = 7.1, 1.1 Hz, H-6), 4.90 (d, 1H, *J* = 10.8 Hz, N-H), 3.98 (s, 3H, H-18), 3.22-3.10 (m, 1H, H-11),

2.00-1.94 (m, 2H, H-12a & H-16a), 1.80-1.73 (m, 2H, H-13a & H-15a), 1.63-1.60 (m, 1H, H-14a), 1.32-1.12 (m, 5H, H-12b, H-13b, H-14b, H-15b, H-16b); ^{13}C NMR (101 MHz, $CDCl_3$) δ 165.1 (C-17), 142.6 (C-5), 138.8 (C-3), 126.5 (C-9), 125.0 (C-7), 124.1 (C-2), 118.2 (C-8), 115.0 (C-6), 60.6 (C-11), 51.9 (C-18), 33.1 (C-12 & C-16), 25.7 (C-14), 25.4 (C-15 & C-13). IR ν_{max}(cm^{-1}) 3305 (N-H), 1691 (C=O), 788 (C-Cl). HRMS (ES$^+$) calculated for $C_{15}H_{19}N_3ClO_2$ $[M+H]^+$: 308.1161, found: 308.1174.

Methyl 5-bromo-3-(cyclohexylamino)imidazo[1,2-*a*]pyridine-2-carboxylate (13d)

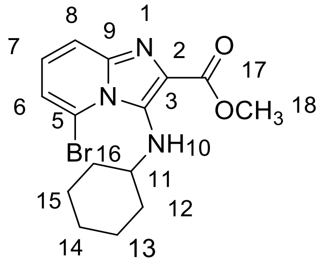

Glyoxylic acid monohydrate (**11**, 660 mg, 8.68 mmol), perchloric acid (1.16 mmol, 100 µL), 2-Amino-6-bromopyridine (**9d**, 1.00 g, 5.78 mmol) and cyclohexyl isocyanide **(10,** 6.54 mmol, 790 µL) were reacted in methanol (12 mL) for 24 h. Compound **13d** was obtained as a dark yellow powder (1.12 g, 55 %). M.p.= 133-135 °C. R_f =0.34 (60% Hex/EtOAC). ^{1}H NMR (400 MHz, $CDCl_3$) δ 7.51 (d, 1H, J = 8.7 Hz, H-8), 7.03-6.92 (m, 2H, H-6 & H-7), 4.84 (d, 1H, J = 10.7 Hz, N-H), 3.98 (s, 1H, H-18), 3.21-3.12 (m, 1H, H-11), 2.02-1.94 (m, 2H, H-12a & H-16a), 1.79-1.69 (m, 2H, H-13a & H15a), 1.32-1.12 (m, 6H, H-12b, H-13b, H-14a, H-14b H15b, H16b); ^{13}C NMR (101 MHz, $CDCl_3$) δ 165.1 (C-17), 142.6 (C-3), 138.9 (C-9), 125.3 (C-7), 124.6 (C-5), 119.8 (C-6), 118.7 (C-8), 112.6 (C-2), 60.5 (C-11), 51.9 (C-18), 32.9 (C-12 & C-16), 25.7 (C-14), 25.5 (C-13 & C-15).). IR ν_{max}(cm^{-1}) 2980 (N-H), 1692 (C=O), 619 (C-Br). HRMS (ES$^+$) calculated for $C_{15}H_{19}N_3{}^{79}BrO_2$ $[M+H]^+$: 352.0656, found: 352.0648.

Methyl 5-fluoro-3-(cyclohexylamino)imidazo[1,2-*a*]pyridine-2-carboxylate (13f)

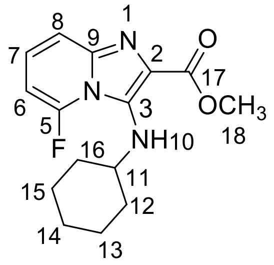

Glyoxylic acid monohydrate (**11**, 594 mg, 7.82 mmol), perchloric acid (1.04 mmol, 90 µL), 2-amino-6-fluoropyridine (**9e**, 670 mg, 5.21 mmol) and cyclohexyl isocyanide (**10**, 713 µL, 5.73 mmol) were reacted in methanol (12 mL) to afford compound **13f** as colourless crystals (831 mg, 51%). M.p.= 230-231 °C. R_f = 0.34 (80% EtOAC/Hex). ^{1}H NMR (400 MHz, DMSO-*d*$_6$) δ 7.82 – 7.72 (m, 1H, H-7), 7.53 – 7.46 (m, 1H, H-8), 7.14 (t, *J* = 7.4 Hz, 1H, H-6), 5.44 (s, 1H, N-H), 3.94 (s, 3H, H-18), 3.31 – 3.20 (m, 1H, H-11), 1.96 – 1.88 (m, 2H, H-12a & H-16a), 1.75 – 1.67 (m, 2H, H-13a & H-15a), 1.59 – 1.52 (m, 1H, H-14a), 1.38 – 1.08 (m, 5H, H-14b, H-12b, H-16b, H-13b & H-15b). ^{13}C NMR (101 MHz, DMSO-*d*$_6$) δ 161.2 (C-17), 150.9 (d, J_{CF} = 269.9 Hz, C-5), 140.1 (d, J_{CF} = 1.7 Hz, C-3) , 137.3 (d, J_{CF} = 3.3 Hz, C-2, 134.5 (d, J_{CF} = 8.8 Hz, C-7), 114.5 (C-9), 110.9 (d, J_{CF} = 4.4 Hz, C-8) , 98.0 (d, J_{CF} = 17.3 Hz, C-6), 59.0 (C-11), 52.8 (C-18), 33.6 (C-12 & C-16), 25.6 (C-14), 25.2 (C-13 & C-15). IR ν_{max}(cm^{-1}) 3305.86 (N-H), 1599.06 (C=C), 1499.33 (N-H bending), 1033.32 (C-F). HRMS (ES$^+$) calculated for $C_{15}H_{18}FN_3O_2$ [M]$^+$: 291.1383, found: 291.1374

Synthesis of imidazo[1,2-*a*]pyridine derivatives unsubstituted at position 2 (12)

General procedure:

Glyoxylic acid monohydrate **11** (1.5 equiv) and an appropriate aminopyridine **9** (1.05 mmol) were dissolved in dry methanol in a round bottom flask. Thereafter, 70% perchloric acid (0.1 equiv) was added and the mixture was allowed to stir at room temperature for 45 minutes. Cyclohexyl isocyanide **10** (1.1 equiv) was then introduced slowly over a period of 10–15 minutes and the reaction was stirred at ambient temperature until completion, usually 20–24 h. The precipitate that formed was filtered and rinsed three times with small amount of hexane/ethyl acetate (50:50) to afford the desired product. Additional product was obtained by concentrating the filtrate and purifying the residue using column chromatography.

6-Chloro-*N*-cyclohexylimidazo[1,2-*a*]pyridin-3-amine (12a)

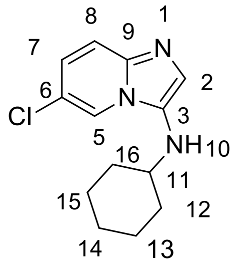

2-Amino-5-chloropyridine (**9a**, 2.00 g, 15.6 mmol), glyoxylic acid monohydrate (**11**, 1.78 g, 23.3 mmol), perchloric acid 70% (134 µL, 1.56 mmol) and cyclohexyl isocyanide (**10**, 2.1 mL, 17.1 mmol) were reacted in methanol (15 mL) at room temperature to afford the product. The yellow crystals were further recrystallized in hot 50% ethyl acetate/hexane to afford **12a** as light-yellow crystals (1.5 g, 37%). M.p.= 150 °C. R_f =0.34 (50% Hex/EtOAC). ^{1}H NMR (400 MHz, $CDCl_3$) δ 8.10-8.08 (m, 1H, H-5), 7.47 (d, 1H, *J* = 9.5 Hz, H-8), 7.22 (s, 1H, H-2), 7.06 (dd, 1H, *J* = 9.5, 2.0 Hz, H-7), 3.09 (br s, 1H, N-H), 3.03-2.94 (m, 1H, H-11), 2.01-1.92 (m, 2H, H-12a & H-16a), 1.82-1.71 (m, 2H, H-13a & H-14a), 1.68-1.58 (m, 1H, H-14a), 1.31-1.21 (m, 5H, H-12b, H-13b, H-14b, H-15b, H-16b); ^{13}C NMR (101 MHz, $CDCl_3$) δ 139.9 (C-9), 130.1 (C-3), 124.6 (C-7), 123.6 (C-2), 120.3 (C-6), 120.3 (C-5), 117.8 (C-8), 56.2 (C-11), 33.8 (C-12 & C-16), 25.8 (C-14), 24.8 (C-13 & C-15). IR ν_{max}(cm^{-1}) 3312 (N-H), 1551 (C=C), 848 (C-Cl). HRMS (ES$^+$) calculated for $C_{13}H_{17}N_3Cl$ [M+H]$^+$: 250.1106, found: 250.1120.

6-Bromo-*N*-cyclohexylimidazo[1,2-*a*]pyridin-3-amine (12b)

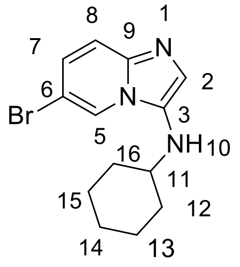

2-Amino-5-bromopyridine (**9b**, 2.00 g, 11.6 mmol), glyoxylic acid monohydrate (**11**, 1.32 g, 17.3 mmol), perchloric acid 70% (100 µL, 1.16 mmol) and cyclohexyl isocyanide (**10**, 1.58 mL, 12,7 mmol) were stirred for 24 h in methanol (15 mL) to afford **12b** as golden-brown crystals (1.7 g, 50%). M.p.= 154-155 °C. R_f =0.35 (50% Hex/EtOAC). ^{1}H NMR (400 MHz, $CDCl_3$) δ 8.15 (d, 1H, *J* = 4 Hz, H-5), 7.40 (d, 1H, *J* = 9.4 Hz, H-8), 7.21 (s, 1H, H-2), 7.13 (dd, 1H, *J* = 9.5, 2.0 Hz, H-7), 3.06-2.95 (m, 1H, N-H), 2.94-2.84 (m, 1H, H-11), 2.04-1.93 (m, 2H, H-12a & H-16a), 1.82-1.78 (m, 2H, H-13a, H-15a), 1.69-1.58 (m, 1H, H-14a), 1.37-1.14 (m, 5H, H-12b, H-13b, H-14b, H15b & H-16b); ^{13}C NMR (101 MHz, $CDCl_3$) δ 140.3 (C-9), 129.7 (C-3), 126.2 (C-7), 124.5 (C-2), 122.4 (C-5), 118.4 (C-8), 106.6 (C-6), 56.3 (C-11), 33.8 (C-12 & C-16), 25.8 (C-14), 24.8 (C13 & C-15). IR ν_{max}(cm^{-1}) 3208 (N-H), 1630 (C=C). 588 (C-Br). HRMS (ES$^+$) calculated for $C_{13}H_{17}N_3{}^{79}Br$ [M+H]$^+$: 294.0601, found: 294.0592.

Alternative method:

6-Bromo-*N*-cyclohexylimidazo[1,2-*a*]pyridin-3-amine (12b)

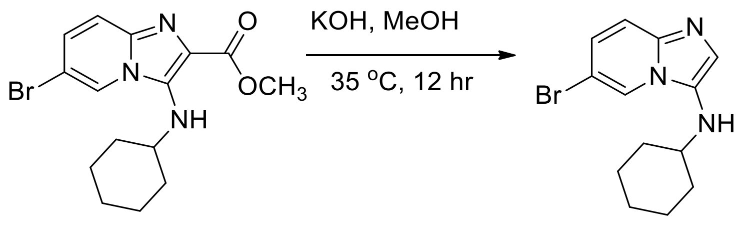

Methyl 6-bromo-3-(cyclohexylamino)imidazo[1,2-*a*]pyridine-2-carboxylate (**13b**, 100 mg, 0.284 mmol) was dissolved in MeOH (2 mL) and 1.4-dioxane (1 mL) at 35 °C with stirring. KOH (32 mg, 0.568 mmol) was added, and the mixture was allowed to stir. After 2 hours, the mixture was quenched with water (4 mL) and stirred for an additional 10 h. After the reaction was completed, the solvent was removed in vacuo and the mixture was acidified with 4 M HCl until the pH was about 1–2. The mixture was extracted with EtOAC, dried over anhydrous $MgSO_4$ and the residue was purified by flash column chromatography using 100 % EtOAc as eluent to afford the product. Compound **12b** was obtained as golden-brown crystals (50 mg, 60%).

5-Chloro-*N*-cyclohexylimidazo[1,2-*a*]pyridin-3-amine (12c)

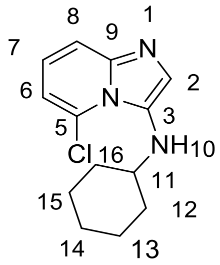

2-Amino-6-chloropyridine (**9c**, 2.00 g, 15.6 mmol), glyoxylic acid monohydrate (**11**, 1.78 g, 23.3 mmol), perchloric acid 70% (134 µL, 1.56 mmol) and cyclohexyl isocyanide (**10**, 2.1 mL, 17.1 mmol) were stirred at room temperature for 24 h. Compound **12c** was obtained as a golden yellow viscous oil (1.2 g, 31%). R_f =0.32 (60% Hex/EtOAC). ^{1}H NMR (400 MHz, $CDCl_3$) δ 7.40 (d, 1H, *J* = 9.0 Hz, H-6), 7.10 (s, 1H, H-2), 6.90 (dd, 1H, *J* = 9.0, 7.2 Hz, H-7), 6.66 (d, 1H, *J* = 7.1 Hz, H-8), 3.83 (s, 1H, N-H), 3.09-2.99 (m, 1H, H-11), 2.02-1.94 (m, 2H, H-12a &

H-16a), 1.82-1.73 (m, 2H, H-13a & H-15a), 1.67-1.59 (m, 1H, H-14a), 1.32-1.22 (m, 5H, H-12b, H-13b, H-14b, H-15b & H-16b); ^{13}C NMR (101 MHz, $CDCl_3$) δ 143.8 (C-5), 132.4 (C-9), 125.1 (C-3), 123.7 (C-2), 122.1 (C-7), 117.2 (C-6), 113.4 (C-8), 56.7 (C-11), 33.1 (C-12 & C-16), 25.9 (C-14), 24.6 (C-13 & C-15). IR ν_{max}(cm^{-1}) 2960 (N-H), 1626 (C=C), 770 (C-Cl). HRMS (ES$^+$) calculated for $C_{13}H_{17}N_3Cl$ [M+H]$^+$: 250.1106, found: 250.1119.

5-Bromo-*N*-cyclohexylimidazo[1,2-*a*]pyridin-3-amine (12d)

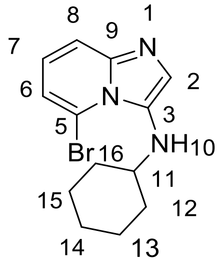

2-Amino-6-bromopyridine (**9d**, 2.00 g, 11.6 mmol), glyoxylic acid monohydrate **(11,** 1.32 g, 17.3 mmol), perchloric acid 70% (100 µL, 1.16 mmol) and cyclohexyl isocyanide (**10**, 1.58 mL, 12,7 mmol) were reacted in methanol (15 mL) for 24 h. Compound **12d** was obtained as tan crystals (1.96 g, 57%). M.p.= 109-111 °C. R_f =0.35 (50% Hex/EtOAC). ^{1}H NMR (400 MHz, $CDCl_3$) δ 7.45 (dd, 1H, *J* = 8.6, 1.5 Hz, H-6), 7.15 (s, 1H, H-2), 6.89-6.80 (m, 2H, H-8 & H-7), 3.80 (s, 1H, N-H), 3.12-2.93 (m, 1H, H-11), 2.03-1.91 (m, 2H, H-12a & H-16a), 1.82-1.67 (m, 2H, H-13a & H-15a), 1.67-1.54 (m, 1H, H-14a), 1.34-1.22 (m, 5H, H-12b, H-13b, H-14b, H-15b & H-16b); ^{13}C NMR (101 MHz, $CDCl_3$) δ 143.9 (C-9), 132.4 (C-5), 124.7 (C-2), 122.5 (C-8), 118.0 (C-7), 117.7 (C-6), 111.3 (C-3), 56.6 (C-11), 33.0 (C-12 & C-16), 26.0 (C-14), 24.6 (C-13 & C-15. IR ν_{max}(cm^{-1}) 3354 (N-H), 1620 (C=C), 676 (C-Br). HRMS (ES$^+$) calculated for $C_{13}H_{17}N_3{}^{79}Br$ [M+H]$^+$: 294.0601, found: 294.0593.

Synthesis of (3-(cyclohexylamino)imidazo[1,2-*a*]pyridin-2-yl)methanol analogues 17

General procedure:

An appropriate amount of the ester was dissolved in dry tetrahydrofuran in an oven dried two-neck flask fitted with a rubber septum. The flask was submerged in an ice bath and allowed to cool to 0 °C. $LiAlH_4$ (1.5 equiv) was added portion-wise with stirring. The flask was completely

evacuated and backfilled with nitrogen. The reaction was allowed to run at room temperature until completion (8–24 h). Thereafter, the mixture was quenched with water until there was no visible fizzing. The mixture was allowed to settle for 30 minutes, and the precipitate formed was filtered through a cotton wool plug and the filtrate was extracted with dichloromethane (3 × 30 mL). The organic layer was dried over anhydrous $MgSO_4$, and the solvent removed in vacuo. The resulting brown solid was recrystallized from boiling EtOAc to give a pure product. Additional product was obtained by purifying the mother liquor using silica gel column chromatography and eluting with $CHCl_3$/EtOAc/MeOH (75:25:5).

(6-Chloro-3-(cyclohexylamino)imidazo[1,2-*a*] pyridin-2-yl)methanol (17a)

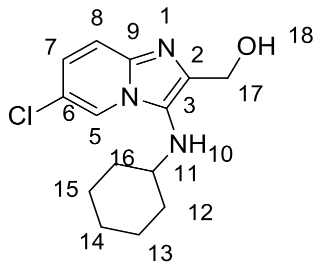

Methyl 6-chloro-3-(cyclohexylamino)imidazo[1,2-*a*]pyridine-2-carboxylate (**13a**, 511 mg, 1.66 mmol) and $LiAlH_4$ (100 mg, 2.64 mmol) were reacted in THF (10 mL) for 8 h. Compound **17a** was obtained as a brown powder (405.9 mg, 95 %). M.p.= 195-197 °C. R_f =0.32 (75:25:5 $CHCl_3$/EtOAc/MeOH). ^{1}H NMR (400 MHz, $CDCl_3$) δ 8.06 (dd, 1H, *J* = 2.1, 0.9 Hz, H-5), 7.41 (dd, 1H, *J* = 9.4, 0.9 Hz, H-8), 7.08 (dd, 1H, *J* = 9.5, 2.0 Hz, H-7), 4.81 (s, 2H, H-17), 3.97 (br s, 1H, OH), 3.07 (d, 1H, *J* = 6.4 Hz, NH), 2.91-2.82 (m, 1H, H-11), 1.93-1.85 (m, 2H, H-12a & H-16a), 1.82-1.75 (m, 2H, H-13a & H-16a), 1.67-1.58 (m, 1H, H-14a), 1.29-1.17 (m, 5H, H-12b, H-13b, H-14b, H-15b & H-16b); ^{13}C NMR (101 MHz, $CDCl_3$) δ 139.8 (C-9), 138.8 (C-3), 125.9 (C-2), 125.2 (C-7), 120.7 (C-5), 120.2 (C-6), 117.7 (C-8), 57.7 (C-17), 57.2 (C-11), 34.2 (C-12 & C-16), 25.7 (C-14), 24.9 (C-13 & C-15).). IR ν_{max}(cm^{-1}) 1567 (C=C), 1321 (OH), 1055 (C-O), 804 (C-Cl). HRMS (ES$^+$) calculated for $C_{14}H_{19}N_3OCl$ [M+H]$^+$: 280.1212, found: 280.1226.

(6-Bromo-3-(cyclohexylamino)imidazo[1,2-*a*]pyridin-2-yl)methanol (17b)

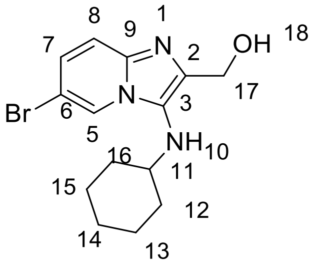

Methyl 6-bromo-3-(cyclohexylamino)imidazo[1,2-*a*]pyridine-2-carboxylate (**13b**, 700 mg, 1.99 mmol) and $LiAlH_4$ (113 mg, 2.98 mmol) were reacted in THF (10 mL) for 12 h. Compound **17b** was obtained as a light brown powder (637.8 mg, 99%). M.p.= 143-144 °C. R_f =0.46 (75:25:5 $CHCl_3$/EtOAc/MeOH). ^{1}H NMR (400 MHz, $CDCl_3$) δ 8.16 (dd, 1H, *J* = 1.9, 0.8 Hz, H-5), 7.36 (dd, 1H, *J* = 9.5, 0.9 Hz, H-8), 7.18 (dd, 1H, *J* = 9.4, 1.9 Hz, H-7), 4.81 (s, 2H, H-17), 3.05 (d, 1H, *J* = 6.2 Hz, N-H), 2.92-2.81 (m, 1H, H-11), 2.18 (s, 1H, OH), 1.96-1.86 (m, 2H, H-12a & H-16a), 1.78-1.72 (m, 2H, H-13a & H-15a), 1.66-1.59 (m, 1H, H-14a), 1.28-1.21 (m, 5H, H-12b, H-13b, H-14b, H-15b, H-16b); ^{13}C NMR (101 MHz, $CDCl_3$) δ 139.9 (C-9), 138.5 (C-3), 127.2 (C-7), 125.7 (C-2), 123.0 (C-5), 118.0 (C-8), 106.7 (C-6), 57.8 (C-17), 57.2 (C-11), 34.2 (C-12 & C-16), 25.7 (C-14), 24.9 (C-13 & C-15). IR ν_{max}(cm^{-1}) 3305 (N-H), 1566 (C=C), 1408 (OH), 1032 (C-O), 530 (C-Br). HRMS (ES^+): calculated for $C_{14}H_{19}N_3O^{79}Br$ $[M+H]^+$: 324.0706, found: 324.0694.

(5-Chloro-3-(cyclohexylamino)imidazo[1,2-*a*]pyridin-2-yl)methanol (17c)

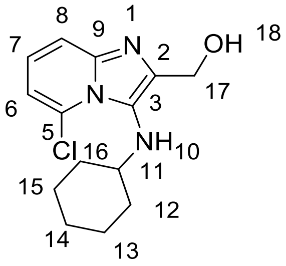

Methyl 5-chloro-3-(cyclohexylamino)imidazo[1,2-*a*]pyridine-2-carboxylate (**13c**, 511 mg, 1.66 mmol) and $LiAlH_4$ (99.7 mg, 2.64 mmol) were reacted in THF (10 mL) for 8 h. Compound **17c** was obtained as a brown powder (427.3 mg, 92%). M.p.= 143-144 °C. R_f =0.32 (75:25:5 $CHCl_3$/EtOAc/MeOH). ^{1}H NMR (400 MHz, $CDCl_3$) δ 7.45 (dd, 1H, *J* = 8.9 Hz, H-8), 7.05 (dd, 1H, *J* = 8.9, 7.2 Hz, H-7), 6.75 (dd, 2H, *J* = 7.4, 0.8 Hz, H-6), 4.86 (s, 2H, H-17), 3.85 (br s, 1H, OH), 3.35 (s, 1H, NH), 3.02-2.91 (m, 1H, H-11), 1.92-1.79 (m, 2H, H-12 a, H-16a), 1.79-

1.72 (m, 2H, H-13a & H-15a), 1.66-1.51 (m, 1H, H-14a), 1.30-1.17 (m, 5H, H-12b, H-13b, H-14b, H-15b & H-16b); ^{13}C NMR (101 MHz, $CDCl_3$) δ 144.3 (C-5), 141.5 (C-9), 125.8 (C-2), 125.7 (C-3), 124.1 (C-7), 116.6 (C-8), 113.8 (C-6), 59.8 (C-17), 58.5 (C-11), 33.0 (C-12 & C-16), 25.8 (C-14), 24.8 (C-13 & C-15). IR ν_{max}(cm^{-1}) 1567 (C=C), 1321 (OH), 1055 (C-O), 804 (C-Cl). HRMS (ES$^+$) calculated for $C_{14}H_{19}N_3OCl$ [M+H]$^+$: 280.1212, found: 280.1197.

(3-(Cyclohexylamino)imidazo[1,2-*a*]pyridin-2-yl)methanol (17d)

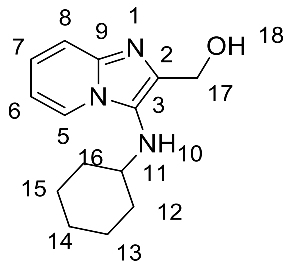

Methyl 5-bromo-3-(cyclohexylamino)imidazo[1,2-*a*]pyridine-2-carboxylate (**13d**, 700 mg, 1.99 mmol) and $LiAlH_4$ (113 mg, 2.98 mmol) were reacted in THF (10 mL) for 12 h. Compound **17d** was obtained as a brown powder (467.6 mg, 96%).

Alternatively:

Methyl 5-fluoro-3-(cyclohexylamino)imidazo[1,2-*a*]pyridine-2-carboxylate (**13f**, 650 mg, 2.23 mmol) and $LiAlH_4$ (127.01 mg, 3.35 mmol) were reacted in THF (10 mL) for 12 h. Compound **17d** was obtained as a brown powder (279.2 mg, 51%). M.p.= 142-144 °C. R_f =0.36 (75:25:5 $CHCl_3$/EtOAc/MeOH). ^{1}H NMR (400 MHz, $CDCl_3$) δ 8.03 (dt, 1H, *J* = 6.8, 1.2 Hz, H-5), 7.47 (dt, 1H, *J* = 9.1, 1.2 Hz, H-8), 7.10 (ddd, 1H, *J* = 9.0, 6.7, 1.3 Hz, H-7), 6.76 (td, 1H, *J* = 6.8, 1.2 Hz, H-6), 5.86 (br s, 1H, OH), 4.83 (s, 2H, H-17), 3.14 (d, 1H, *J* = 6.3 Hz, NH), 2.92-2.80 (m, 1H, H-11), 1.95-1.84 (m, 2H, H-12a & H-16a), 1.77-1.69 (m, 2H, H-13a & H-15a), 1.64-1.56 (m, 1H, H-14a), 1.31-1.12 (m, 5H, H-12b, H-13b, H-14b, H-15b & H-16b). ^{13}C NMR (101 MHz, $CDCl_3$) δ 141.5 (C-9), 137.9 (C-3), 125.5 (C-2), 123.8 (C-5), 122.8 (C-7), 117.1 (C-8), 111.6 (C-6), 57.2 (C-11), 57.1 (C-17), 34.2 (C-12 & C-16), 25.7 (C-14), 24.9 (C-13 & C-15). IR ν_{max}(cm^{-1}) 3247 (N-H), 1345 (OH), 1634 (C=C), 1054 (C-O). HRMS (ES$^+$) calculated for $C_{14}H_{20}N_3O$ [M+H]$^+$: 246.1601, found: 246.1604.

Synthesis of 2-bromomethyl substituted imidazo[1,2-*a*]pyridin-3-amines 21

General procedure:

The appropriate alcohol **17** was dissolved in dry DCM or chloroform in a two-neck flask fitted with a threaded rubber septum at one end. The flask was evacuated and backfilled with nitrogen. Phosphorus tribromide (1.5 equiv) was introduced dropwise with constant stirring. The reaction mixture was allowed to stir at room temperature for 6–8 h and thereafter the solvent was completely removed using a rotary evaporator. The product was used directly in the next step without further purification.

6-Chloro-2-(bromomethyl)-*N*-cyclohexylimidazo[1,2-*a*]pyridin-3-amine (21a)

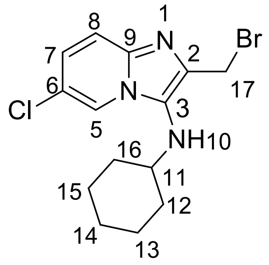

To a solution of (6-choro-3-(cyclohexylamino)imidazo[1,2-*a*] pyridin-2-yl)methanol (**17a**, 150 mg, 0.536 mmol) in dry DCM (7 mL) was introduced phosphorus tribromide (217.7 mg, 0.804 mmol) dropwise with constant stirring. The reaction mixture was stirred at room temperature for 8 h and then concentrated under reduced pressure to afford a thick brown mass.

6-Bromo-2-(bromomethyl)-*N*-cyclohexylimidazo[1,2-*a*]pyridin-3-amine (21b)

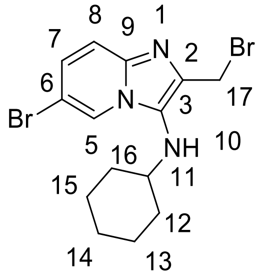

To a solution of (6-bromo-3-(cyclohexylamino)imidazo[1,2-*a*] pyridin-2-yl)methanol (**17b**, 232 mg, 0.716 mmol) in dry DCM (10 mL), was introduced phosphorus tribromide (113 mg, 0.787 mmol) dropwise with constant stirring. The reaction mixture was stirred at room temperature for 8 h and the solvent was completely removed to afford a brown residue.

5-Chloro-2-(bromomethyl)-*N*-cyclohexylimidazo[1,2-*a*]pyridin-3-amine (21c)

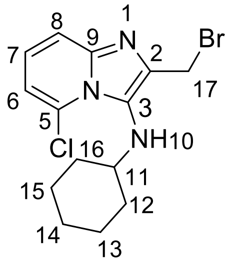

To a solution of (5-chloro-3-(cyclohexylamino)imidazo[1,2-*a*] pyridin-2-yl)methanol (**17c**, 150 mg, 0.536 mmol) in dry DCM (7 mL) was introduced phosphorus tribromide (217.7 mg, 0.804 mmol) dropwise with constant stirring. The reaction mixture was stirred at room temperature for 8 h and the solvent removed under reduced pressure to afford a brown residue.

2-(Bromomethyl)-*N*-cyclohexylimidazo[1,2-*a*]pyridin-3-amine (21d)

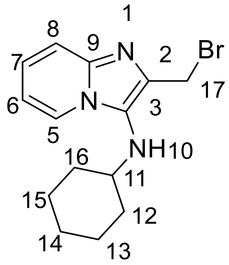

To a solution of (3-(cyclohexylamino)imidazo[1,2-*a*] pyridin-2-yl)methanol (**17d**, 116 mg, 0.358 mmol) in dry DCM (5 mL) was introduced phosphorus tribromide (56.5 mg, 0.394 mmol) dropwise with constant stirring. The reaction mixture was stirred at room temperature for 8 h and the solvent removed using a rotary evaporator to leave behind a thick brown residue.

Synthesis of imidazo[1,2-*a*]pyridine-2-carbaldehydes (20)

Microwave assisted preparation of 3-cyclohexylaminoimidazo[1,2-*a*]pyridine-2-carbaldehydes 20

General procedure:

An appropriate amount of the crude 2-(bromomethyl)-*N*-cyclohexylimidazo[1,2-*a*]pyridin-3-amine (**21**) was dissolved in dry DMSO in a 50 mL long-neck flask fitted with a magnetic stirrer bar. The mixture was stirred at room temperature for 5 minutes, and thereafter KI (1.5 equiv) and $NaHCO_3$ (4 equiv) were added. The mixture was heated to 110 °C at 150 W in an open vessel microwave reactor and allowed to react for 15 minutes. Thereafter the reaction

mixture was cooled to room temperature, transferred into a separating funnel and 20 mL of water was added. The mixture was extracted with EtOAC (3 × 20 mL). The combined organic fractions were washed with brine (30 mL) and dried over anhydrous $MgSO_4$. The solvent was removed in vacuo and the crude product was purified by silica gel column chromatography using 50:50 EtOAC/hexane to afford the desired aldehyde product.

6-Chloro-3-(cyclohexylamino)imidazo[1,2-*a*]pyridine-2-carbaldehyde (20a)

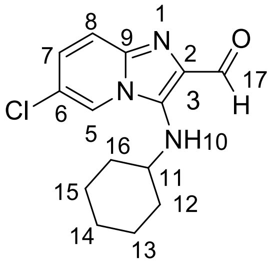

6-Chloro-2-(bromomethyl)-*N*-cyclohexylimidazo[1,2-*a*]pyridin-3-amine (**21a**, 120 mg, 0.350 mmol), KI (107.6 mg, 0.648 mmol) and $NaHCO_3$ (145.2 mg, 1.73 mmol) were dissolved in DMSO (5 mL). Compound **20a** was obtained as a yellowish-brown solid (50.4 mg, 35%). M.p. = 147.5-148.2 °C. R_f = 0.66 (70% $CHCl_3$/EtOAC). ^{1}H NMR (400 MHz, $CDCl_3$) δ 10.08 (s, 1H, H-17), 7.94 (d, *J* = 2.0 Hz, 1H, H-5), 7.45 (d, *J* = 9.7 Hz, 1H, H-8), 7.09 (dd, *J* = 9.7, 2.0 Hz, 1H, H-7), 5.50 (d, *J* = 10.0 Hz, 1H, N-H), 3.34 – 3.26 (m, 1H, H-11), 1.96 – 1.90 (m, 2H, H-12a & H-16a), 1.79 – 1.73 (m, 2H, H-13a & H-15a), 1.65 – 1.57 (m, 1H, H-14a), 1.39 – 1.25 (m, 5H, H-14b, H-12b, H-16b, H-13b & H-15b). ^{13}C NMR (101 MHz, $CDCl_3$) δ 189.0 (C-17), 139.3 (C-3), 136.7 (C-9), 129.8 (C-2), 126.8 (C-7), 121.6 (C-6), 121.3 (C-5), 120.0 (C-8), 55.1 (C-11), 34.1 (C-12 & C-16), 25.4 (C-14), 24.6 (C-13 & C-15). IR ν_{max}(cm^{-1}) 3313.31-3680.27 (N-H), 1693.81 (C=O), 1656.40 (C=C). HRMS (ES^+) calculated for $C_{14}H_{16}ClN_3O$ $[M]^+$: 277.0982, found: 277.0976.

Preparation by conventional heating

General procedure:

An appropriate amount of the crude 2-(bromomethyl)-*N*-cyclohexylimidazo[1,2-*a*]pyridin-3-amine (**21**) was dissolved in dry DMSO in a 50 mL two-neck flask fitted with a magnetic stirrer bar. The mixture was stirred at room temperature for 5 minutes, and thereafter KI (1.5 equiv) and $NaHCO_3$ (4 equiv) were added. The mixture was heated to 96 °C and the reaction was allowed to run for 20 h. The reaction was then cooled and allowed to stir at room temperature for a further 4 h. The solvent was removed in vacuo and the residue was taken up in EtOAC. Water was added, and the mixture was separated using a separatory funnel. The organic layers were combined, dried over anhydrous $MgSO_4$ and concentrated in vacuo. The crude product was purified by silica gel column chromatography using $CHCl_3$/EtOAC/MeOH 70:25:5 to afford the product.

6-Bromo-3-(cyclohexylamino)imidazo[1,2-*a*]pyridine-2-carbaldehyde (20b)

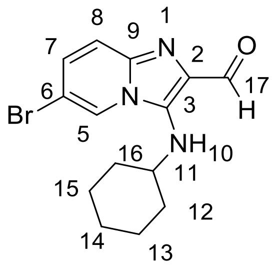

6-Bromo-2-(bromomethyl)-*N*-cyclohexylimidazo[1,2-*a*]pyridin-3-amine (**21b**, 277 mg, 0.716 mmol), $NaHCO_3$ (72 mg, 0.859 mmol) and KI (1.07 mmol, 178.28 mg) were dissolved in DMSO (7 mL). Compound **20b** was obtained as a yellow solid (129 mg, 56%). M.p.= 161-163 °C. R_f =0.52 (70% Hex/EtOAc). ^{1}H NMR (400 MHz, $CDCl_3$) δ 10.09 (s, 1H, H-17), 8.02 (s, 1H, H-5), 7.38 (d, 1H, J = 9.7 Hz, H-8), 7.16 (dd, 1H, J = 9.7, 1.7 Hz, H-7), 5.47 (d, 1H, N-H), 3.33-3.25 (m, 1H, H-11), 1.96-1.89 (m, 2H, H-12a & H-16a), 1.80-1.73 (m, 2H, H-13a & H-15a), 1.64-1.59 (m, 1H, H-14a), 1.45-1.17 (m, 5H, H-12b, H-13b, H-14b, H-15b & H-16b); ^{13}C NMR (101 MHz, $CDCl_3$) δ 189.1 (C-17), 139.3 (C-3), 136.5 (C-9), 129.7 (C-2), 128.6 (C-7), 123.6 (C-5), 120.3 (C-8), 108.1 (C-6), 55.1 (C-11), 34.1 (C-12 & C-16), 25.4 (C-14), 24.6 (C-13 & C-15). IR ν_{max}(cm^{-1}) 3290 (N-H), 1633(C=C), 1734 (C=O), 517 (C-Br). HRMS (ES$^+$) calculated for $C_{14}H_{17}{}^{79}BrN_3O$ [M+H]$^+$: 322.0550, found: 322.0565.

5-Chloro-3-(cyclohexylamino)imidazo[1,2-*a*]pyridine-2-carbaldehyde (20c)

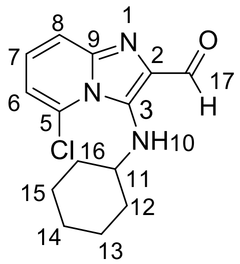

5-Chloro-2-(bromomethyl)-*N*-cyclohexylimidazo[1,2-*a*]pyridin-3-amine (**21c**, 300 mg, 1.08 mmol), KI (268.9 mg, 1.62 mmol) and $NaHCO_3$ (362.9 mg, 4.32 mmol) were dissolved in DMSO (5 mL). Compound **20c** was obtained as a yellowish-brown solid (135 mg, 45%). M.p. = 146-148 °C. R_f = 0.59 (70% $CHCl_3$/EtOAC). ^{1}H NMR (400 MHz, $CDCl_3$) δ 10.12 (s, 1H, H-17), 7.43 (d, *J* = 9.2 Hz, 1H, H-8), 7.03 (dd, *J* = 9.2, 7.1 Hz, 1H, H-7), 6.75 (d, *J* = 7.1 Hz, 1H, H-6), 5.13 (d, *J* = 10.0 Hz, 1H, NH), 3.41 – 3.29 (m, 1H, H-11), 2.01 – 1.92 (m, 2H, H-12a & H-16a), 1.79 – 1.70 (m, 2H, H-13a & H-15a), 1.64 – 1.60 (m, 1H, H-14a), 1.29 – 1.21 (m, 5H, H-12b, H-16b, H-13b, H-15b & H14b). ^{13}C NMR (101 MHz, $CDCl_3$) δ 188.6 (C-17), 143.3 (C-5), 138.1 (C-3), 131.1 (C-9), 126.4 (C-2), 125.1 (C-7), 118.5 (C-8), 115.3 (C-6), 60.2 (C-11), 33.3 (C-12 & C-16), 25.6 (C-14), 25.2 (C-13 & C-15). IR ν_{max}(cm^{-1}) 3312.21 (N-H), 1714.24 (C=O), 2851.80 (C-H, aldehyde), 810.68 (C-Cl). HRMS (ES^+) calculated for $C_{14}H_{16}ClN_3O$ $[M]^+$: 277.0982, found: 277.0966.

3-(Cyclohexylamino)imidazo[1,2-*a*]pyridine-2-carbaldehyde (20d)

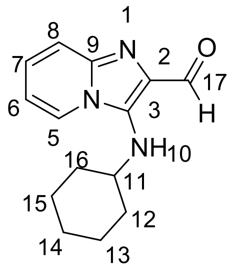

2-(Bromomethyl)-*N*-cyclohexylimidazo[1,2-*a*]pyridin-3-amine (**21d**, 52 mg, 0.161 mmol) was dissolved in 50/50 dioxane-chloroform in a two-neck flask. To it was added DMSO (3 mL) and the mixture was stirred at room temperature for 30 minutes. Thereafter, $NaHCO_3$ (32 mg, 0.387 mmol) dissolved in water (2 mL), was added to the mixture. Reaction proceeded as per

the procedure for **20b**. Compound **20d** was obtained as a yellow powder (23 mg, 57%). ^{1}H NMR (400 MHz, $CDCl_3$) δ 10.10 (s, 1H, H-17), 7.91 (d, *J* = 7.0 Hz, 1H, H-5), 7.48 (d, *J* = 9.3 Hz, 1H, H-8), 7.12 (ddd, *J* = 9.3, 6.5, 1.2 Hz, 1H, H-7), 6.77 (td, *J* = 6.6, 1.1 Hz, 1H, H-6), 5.57 (d, *J* = 7.9 Hz, 1H, NH), 3.39-3.27 (m, 1H, H-11), 1.98-1.89 (m, 2H, H-12a & H-16a), 1.81-1.71 (m, 2H, H-13a & H-15a), 1.65-1.55 (m, 1H, H-14a), 1.42-1.18 (m, 5H, H-12b, H-13b, H-14b, H-15b & H-16b). Compound **20d** was used crude in the subsequent reaction.

Oxidative ring-opening of imidazopyridine alcohols to oxalamides

General procedure using pyridinium chlorochromate (PCC):
An appropriate amount of the imidazopyridine alcohol was dissolved in dry chloroform (12 mL) in an oven dried two-neck flask fitted with a rubber septum. To this was added an equivalent amount of flash silica gel, followed by PCC (1.1 equiv). The mixture was degassed, flushed with nitrogen and allowed to react for 4 h or until a new spot was clearly visible by TLC analysis. After the reaction was completed, the solid was removed by filtration and rinsed three times with chloroform. The filtrate was then concentrated in vacuo and the residue was purified by silica gel column chromatography (70% $CHCl_3$/EtOAc). The colourless fluffy solid material was subsequently recrystallized from hot hexane to afford the pure product.

N¹-(5-Chloropyridin-2-yl)-*N²*-cyclohexyloxalamide (18a)

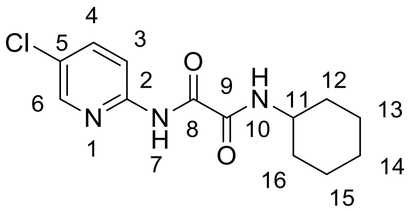

(6-Chloro-3-(cyclohexylamino)imidazo[1,2-*a*]pyridin-2-yl)methanol (**17a**, 209 mg, 0.747 mmol) and PCC (338 mg, 1.57 mmol) were reacted in DCM (10 mL) for 4 h. Compound **18a** was obtained as a colourless fluffy solid. Upon recrystallization from cyclohexane/ethyl acetate, pure colourless and lustrous crystals were obtained (80 mg, 26%). M.p.= 182-184 °C. R_f =0.59 (70% $CHCl_3$/EtOAc). ^{1}H NMR (400 MHz, $CDCl_3$) δ 9.79 (s, 1H, H-7), 8.32 (d, 1H, *J* = 2.7, H-6), 8.18 (d, 1H, *J* = 8.8, H-3), 7.72 (dd, 1H, *J* = 8.8, 2.6 Hz, H-4), 7.35 (d, 1H, *J* = 8.8 Hz, H-10), 3.88-3.74 (m, 1H, H-11), 2.00-1.91 (m, 2H, H-12a & H-16a), 1.83-1.71 (m, 2H, H-13a & H-15a), 1.70-1.61 (m, 1H, H-14a), 1.43-1.20 (m, 5H, H-12b, H-13b, H-14b, H15b,

H16b); ^{13}C NMR (101 MHz, $CDCl_3$) δ 158.3 (C-8), 158.0 (C-9), 148.3 (C-2), 147.2 (C-6), 138.0 (C-4), 127.9 (C-5), 114.5 (C-3), 49.2 (C-11), 32.6 (C-12 & C-16), 25.3 (C-14), 24.7 (C-13 & C-15). IR ν_{max}(cm^{-1}) 3318 (N-H), 1656 (C=O), 743 (C-Cl). HRMS (ES^+) calculated for $C_{13}H_{17}N_3O_2Cl$ [M+H] $^+$: 282.1004, found: 282.1002.

N¹-(5-Bromopyridin-2-yl)-*N²*-cyclohexyloxalamide (18b)

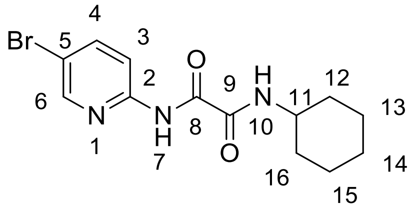

(6-Bromo-3-(cyclohexylamino)imidazo[1,2-*a*]pyridin-2-yl)methanol (**17b**, 555 mg, 1.58 mmol) and PCC (7.14 mg, 3.31 mmol) were reacted in dry chloroform for 4 h to afford **18b** as a colourless fluffy solid (186 mg, 36%). M.p.= 188-190 ˚C. R_f =0.64 (70% $CHCl_3$/EtOAc). ^1H NMR (400 MHz, $CDCl_3$) δ 9.77 (s, 1H, H-7), 8.42 (dd, *J* = 2.5, 0.7 Hz, 1H, H-6), 8.14 (dd, 1H, *J* = 8.8, 0.7 Hz, H-3), 7.86 (dd, 1H, *J* = 8.8, 2.4 Hz, H-4), 7.34 (d, 1H, *J* = 8.7 Hz, H-10), 3.88-3.73 (m, 1H, H-11), 2.01-1.91 (m, 2H, H-12a & H-16a) 1.83-1.71 (m, 2H, H-13a & H-15a), 1.70-1.61 (m, 1H, H-14a), 1.43-1.19 (m, 5H, H-12b, H-13b, H-14b, H-15b, H-16b); ^{13}C NMR (101 MHz, $CDCl_3$) δ 158.3 (C-8), 157.9 (C-9), 149.4 (C-6), 148.6 (C-2), 140.8 (C-4), 115.9 (C-5), 115.0 (C-3), 49.2 (C-11), 32.6 (C-12 & C-16), 25.3 (C-14), 24.7 (C-13 & C-15). IR ν_{max}(cm^{-1}) 3312 (N-H), 1657 (C=O), 690 (C-Br). HRMS (ES^+) calculated for $C_{13}H_{17}N_3O_2{}^{79}Br$ $[M+H]^+$: 326.0499, found: 326.0482.

N¹-(6-Chloropyridin-2-yl)-*N²*-cyclohexyloxalamide (18c)

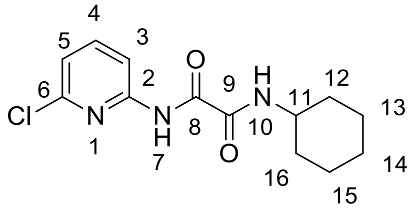

(5-Chloro-3-(cyclohexylamino)imidazo[1,2-*a*]pyridin-2-yl)methanol (**17c**, 209 mg, 0.7470 mmol) and PCC (338 mg, 1.57 mmol) were reacted in DCM (10 mL) for 4 h. Compound **18c** was obtained as a colourless fluffy solid (75.8 mg, 36%). M.p.= 157-159 °C. R_f =0.59 (70% $CHCl_3$/EtOAc). ^1H NMR (400 MHz, $CDCl_3$) δ 9.79 (s, 1H, H-7), 8.14 (d, 1H, *J* = 8.1 Hz, H-

3), 7.72 (t, 1H, *J* = 8.0 Hz, H-4), 7.32 (d, 1H, *J* = 8.8 Hz, H-10), 7.15 (d, 1H, *J* =7.7 Hz, H-5), 3.88-3.74 (m, 1H, H-11), 2.00-1.91 (m, 2H, H-12a & H-16a), 1.83-1.71 (m, 2H, H-13a & H-15a), 1.70-1.61 (m, 1H, H-14a), 1.43-1.13 (m, 5H, H-12b, H-13b, H-14b, H-15b & H-16b); ^{13}C NMR (101 MHz, $CDCl_3$) δ 158.4 (C-8), 157.7 (C-9), 149.8 (C-2), 149.6 (C-6), 140.9 (C-4), 120.9 (C-5), 112.0 (C-3), 49.1 (C-11), 32.6 (C-12 & C-16), 25.3 (C-14), 24.7 (C-13 & C-15). IR ν_{max}(cm^{-1}) 3261 (N-H), 1665 (C=O), 792 (C-Cl). HRMS (ES$^+$) calculated for $C_{13}H_{17}N_3O_2Cl$ [M+H]$^+$: 282.1004, found: 282.1013.

N^1-Cyclohexyl-*N*^{2}-(pyridin-2-yl)oxalamide (18d)

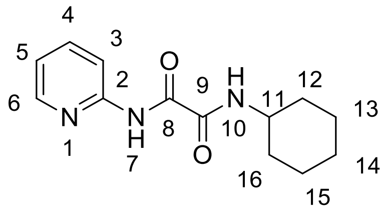

3-(Cyclohexylamino)imidazo[1,2-*a*] pyridin-2-yl)methanol (**17d**, 428 mg, 1.74 mmol) and PCC (413 mg, 1.91 mmol) were reacted in chloroform (12 mL) for 5 h to afford **18d** as a colourless fluffy solid (142 mg, 33%). M.p.= 142-144 °C. R_f =0.63 (70% $CHCl_3$/EtOAc). ^{1}H NMR (400 MHz, $CDCl_3$) δ 9.77 (s, 1H, H-7), 8.37 (dd, 1H, *J* = 5.0, 1.8 Hz, H-6), 8.20 (d, 1H, *J* = 8.3 Hz, H-3), 7.76 (td, 1H, *J* = 7.9, 1.9 Hz, H-4), 7.39 (d, 1H, *J* = 8.7 Hz, H-10), 7.12 (qd, 1H, *J* = 7.4, 4.9, 0.9 Hz, H-5), 3.88-3.75 (m, 1H, H11), 2.02-1.91 (m, 2H, H-12a & H-16a), 1.84-1.71 (m, 2H, H-13a & H-15a), 1.70-1.64 (1H, m, H-14a), 1.48-1.14 (m, 5H, H-12b, H-13b, H-14b, H-15b, H-16b); ^{13}C NMR (101 MHz, $CDCl_3$) δ 158.3 (C-8), 158.2 (C-9), 150.0 (C-2), 148.5 (C-6), 138.4 (C-4), 120.7 (C-5), 113.9 (C-3), 49.1 (C-11), 32.7 (C-12 & C-16), 25.3 (C-14), 24.7 (C-13 & C-15). IR ν_{max}(cm^{-1}) 3317 (N-H), 1658 (C=O). HRMS (ES$^+$) calculated for $C_{13}H_{18}N_3O_2$ [M+H]$^+$: 248.1394, found: 248.1425.

Alternative methods:

Oxidative ring-opening of imidazopyridine alcohols with 2-iodoxybenzoic acid (IBX)

(6-Chloro-3-(cyclohexylamino)imidazo[1,2-*a*]pyridin-2-yl)methanol (**17a**, 1.00g, 3.57 mmol) was dissolved in dry $CHCl_3$ (15 mL) in a two-neck flask fitted with a threaded sleeve septum. To this was added glacial acetic acid (0.3 mL, 1.2 equiv) and IBX (1.06 g, 1.2 equiv), which

was dissolved in dry acetonitrile. The mixture was heated at 80 °C and the reaction was allowed to proceed overnight. After reaction completion, the solvent was removed *in vacuo* to afford a dark syrup. This was taken up in 70% $CHCl_3$/EtOAc and purified by silica gel column chromatography using 70% $CHCl_3$/EtOAc as eluent. Fractions containing the product were combined and solvent removed *in vacuo* to afford a colourless to yellowish fluffy material (285 mg). This was recrystallized in cyclohexane to afford the product (**18a**) as a clear colourless fluffy solid (260 mg, 26 %).

Oxidative ring-opening of imidazopyridine alcohols using laccase from *T. versicolor*

3-(Cyclohexylamino)imidazo[1,2-*a*] pyridin-2-yl)methanol (**17d,** 297 mg, 1.21 mmol) was dissolved in THF (7 mL) in a round bottom flask. In a separate flask, laccase enzyme from *T. versicolor* (297 mg), TEMPO (59 mg, 20%) and Tris buffer pH 7.0 were mixed together. The contents of the two flasks were combined and allowed to stir in open air at room temperature. After 10 minutes, water (15 mL) was added, and the mixture was extracted with DCM (3 × 15 mL). The combined organic layer was dried over anhydrous $MgSO_4$, and the solvent was removed in vacuo. The residue was taken up in 70% $CHCl_3$/EtOAc and purified by silica gel column chromatography. Fractions containing the product were collected and solvent removed in vacuo to afford **18d** as a colourless fluffy solid (80 mg, 27%).

Reductive amination reactions

General procedure:

In a 50 mL round bottom flask charged with a magnetic stirrer bar and dried molecular sieves 4 Å, was added the aldehyde **20** and MeOH (10 mL). Acetic acid was added to the solution dropwise until the pH was approximately 5. The mixture was subsequently stirred at room temperature for 30 minutes. Thereafter, 2-chloroaniline (1.5 equiv) was added and the reaction mixture was stirred further for 45 minutes. Sodium triacetoxyborohydride or sodium cyanoborohydride (1.5 equiv) was introduced and the mixture was allowed to stir for a further 12–24 h. The thick creamy precipitate together with the molecular sieves was removed by filtration and rinsed several times with MeOH. The filtrate was concentrated in vacuo and the residue was taken up in EtOAc. To this was added a saturated solution of $NaHCO_3$ portion-wise until no fizzing was observed. The mixture was extracted with EtOAC (3 × 15 mL) and the combined organic layer was washed with water. The organic layer was dried over

anhydrous $MgSO_4$, concentrated in vacuo, and purified by silica gel column chromatography (60% Hex/EtOAc) to afford the product.

6-Chloro-2-((2-chlorophenyl)amino)methyl)-*N*-cyclohexylimidazo[1,2-*a*]pyridin-3-amine (8a)

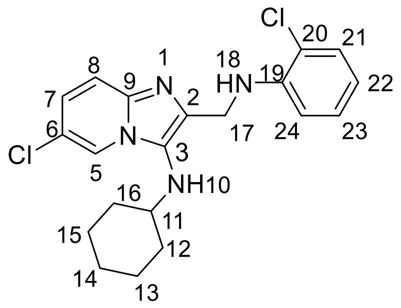

6-Chloro-3-(cyclohexylamino)imidazo[1,2-*a*]pyridine-2-carbaldehyde (**20a**, 80.4 mg, 0,289 mmol), acetic acid (sufficient to adjust the pH to 5), 2-chloroaniline (55.4 mg, 0.434 mmol) and sodium triacetoxyborohydride (91.9 mg, 0.434 mmol) were allowed to stir at room temperature for 24 h. The mixture was filtered, concentrated in vacuo and the residue taken up in methanol (1 mL) and transferred into a mortar. Flash silica gel (200 mg), aluminium oxide (80 mg), boric acid (20 mg) and sodium borohydride (80 mg, 2.11 mmol) were added and the mixture was ground for approximately 3 h after which TLC analysis showed no visible traces of the intermediate imine. The mixture was filtered and purified by column chromatography to afford compound **8a** as a light brown solid (77 mg, 69%). M.p. = 196-198 °C R_f = 0.50 (70% Hex/EtOAC). ^{1}H NMR (400 MHz, $CDCl_3$) δ 8.07 (d, *J* = 2.3 Hz, 1H, H-5), 7.45 (d, *J* = 9.5 Hz, 1H, H-8), 7.29 – 7.25 (m, 1H, H-23), 7.17 – 7.12 (m, 1H, H-21), 7.09 (dd, *J* = 9.4, 2.0 Hz, 1H, H-7), 6.78 (dd, J = 8.1, 1.5 Hz, 1H, H-24), 6.65 (td, *J* = 7.6, 1.5 Hz, 1H, H-22), 4.98 (s, 1H, NH), 4.51 (s, 2H, H-17), 3.02 – 2.83 (m, 2H, NH & H-11), 1.89 – 1.83 (m, 2H, H-12a & H-16a), 1.76 – 1.70 (m, 2H, H-13a & H-15a), 1.66 – 1.59 (1H, m, H-14a), 1.28 – 1.16 (m, 5H, H-14b, H-12b, H-16b, H-13a and H15b). ^{13}C NMR (101 MHz, $CDCl_3$) δ 143.9 (C-9), 139.8 (C-19), 136.5 (C-3), 129.2 (C-7), 127.8 (C-21), 126.0 (C-2), 125.1 (C-23), 120.6 (C-22), 120.2 (C-6), 119.6 (C-5), 117.8 (C-20), 117.7 (C-8), 111.6 (C-24), 57.3 (C-11), 41.8 (C-17), 34.2 (C-12 & C-16), 25.7 (C-14), 24.8 (C-15 & C-13). IR ν_{max}(cm^{-1}) 3019.80 (N-H), 1710.43 (C=C), 1362.79 (N-H bending), 744.29-667.90 (C-Cl). HRMS (ES$^+$) calculated for $C_{20}H_{23}Cl_2N_4$ [M+H]$^+$: 389.1294, found: 389.1265.

6-Bromo-2-((2-chlorophenyl)amino)methyl)-*N*-cyclohexylimidazo[1,2-*a*]pyridin-3-amine (8b)

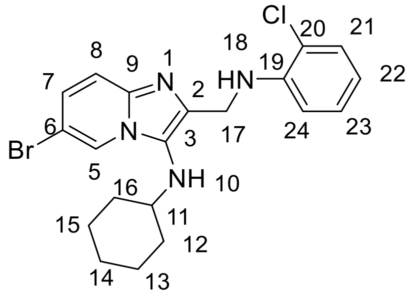

6-Bromo-3-(cyclohexylamino)imidazo[1,2-*a*]pyridine-2-carbaldehyde (**20b**, 129 mg, 0.400 mmol) acetic acid (sufficient to adjust the pH to 5), 2-chloroaniline (77 mg, 0.600 mmol) and sodium cyanoborohydride (34 mg, 0.600 mmol) were allowed to stir at room temperature for 12 h to afford compound **8b** as a light brown lustrous solid (96 mg, 56%). R_f =0.64 (60% EtOAc/Hex). ^{1}H NMR (400 MHz, $CDCl_3$): δ_H = 8.16 (s, 1H, H-5), 7.39 (d, 1H, *J* = 9.4 Hz, H-8), 7.29-7.25 (m, 1H, H-23), 7.20-7.11 (m, 2H, H-7 & H-21), 6.78 (d, 1H, *J* = 8.2 Hz, H-24), 6.66 (t, 1H, *J* = 7.7 Hz, H-22), 4.98 (s, 1H, N-H)), 4.50 (s, 2H, H-17), 2.98-2.86 (m, 2H, N-H & H-11), 1.90-1.79 (m, 2H, H-12a & H-16a), 1.78-1.67 (m, 2H, H-13a & H-15a), 1.65-1.58 (m, 1H, H-14a), 1.28-1.12 (m, 5H, H-12b, H-13b, H-14b, H-15b & H-16b); ^{13}C NMR (101 MHz, $CDCl_3$): δ_C =143.9 (C-9), 139.9 (C-19), 136.4 (C-3), 129.2 (C-23), 127.8 (C-21), 127.1 (C-7), 125.8 (C-2), 122.8 (C-5), 119.5 (C-20), 118.0 (C-8), 117.7 (C-22), 111.6 (C-4), 106.7 (C-6), 57.3 (C-11), 41.8 (C-17), 34.2 (C-12 & C-16), 25.7 (C-14), 24.9 (C-13 & C-15). IR ν_{max}(cm^{-1}) 3305 (N-H), 1599 (C=C), 796 (C-Br). HRMS (ES$^+$) calculated for $C_{20}H_{23}{}^{79}BrClN_4$ [M+H]$^+$: 435.0769, found: 435.0748.

5-Chloro-2-((2-chlorophenyl)amino)methyl)-*N*-cyclohexylimidazo[1,2-*a*]pyridin-3-amine (8c)

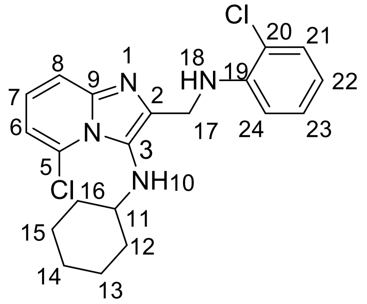

5-Chloro-3-(cyclohexylamino)imidazo[1,2-*a*]pyridine-2-carbaldehyde (**20c**, 100 mg, 0,360 mmol), acetic acid (sufficient to adjust the pH to 5), 2-chloroaniline (68.9 mg, 0.540 mmol) and sodium triacetoxyborohydride (114.5 mg, 0.540 mmol) were reacted at room temperature for 24 h. Following the general procedure, compound **8c** was obtained as a light brown solid (91.1 mg, 65%). M.p. = 193-196 °C R_f = 0.66 (60% Hex/EtOAC). ^{1}H NMR (400 MHz, $CDCl_3$) δ 8.04 (d, *J* = 6.8 Hz, 1H), 7.51 (d, *J* = 9.1 Hz, 1H), 7.29 – 7.26 (m, 1H), 7.19 – 7.09 (m, 2H), 6.83 – 6.78 (m, 1H), 6.65 (t, *J* = 7.6 Hz, 1H), 5.02 (s, 1H, NH), 4.52 (d, *J* = 4.0 Hz, 2H, H-17), 2.99 – 2.84 (m, 2H, NH & H-11), 1.89 – 1.83 (m, 2H, H-12a & H-16a), 1.76 – 1.70 (m, 2H, H-13a & H-15a), 1.64 – 1.57 (m, 1H, H-14a), 1.28 – 1.16 (m, 5H, H-12b, H-13b, H-14b, H-15b & H-16b). ^{13}C NMR (101 MHz, $CDCl_3$) δ 144.0 (C-5), 141.6 (C-9), 135.3 (C-19), 129.1 (C-7), 127.8 (C-6), 125.4 (C-3), 123.7 (C-24), 122.6 (C-8), 119.5 (C-2), 117.5 (C-22), 117.4 (C-21), 111.6 (C-20), 111.5 (C-23), 57.2 (C-11), 41.8 (C-17), 34.2 (C-12 & C-16), 25.7 (C14), 24.9 (C-15 & C-13). IR ν_{max}(cm^{-1}) 3295.98 (N-H), 2927.45-2853.18 (sp^3 C-H), 890.63 (C-Cl). HRMS (ES$^+$) calculated for $C_{20}H_{23}Cl_2N_4$ [M+H]$^+$: 389.1294, found: 389.1255.

2-((2-Chlorophenyl)amino)methyl)-*N*-cyclohexylimidazo[1,2-*a*]pyridin-3-amine (8d)

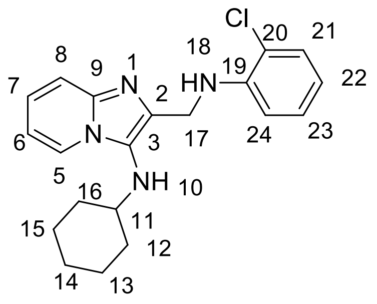

3-(Cyclohexylamino)imidazo[1,2-*a*]pyridine-2-carbaldehyde (**20d**, 129 mg, 0,400 mmol), acetic acid (sufficient to adjust the pH to 5), 2-chloroaniline (155 mg, 1.22 mmol) and sodium cyanoborohydride (76 mg, 1.21 mmol) were allowed to stir at room temperature for 12 h to afford compound **8d** as a brown oil (71 mg, 36%). R_f =0.64 (60% EtOAc/Hex). ^{1}H NMR (400 MHz, $CDCl_3$) δ 8.04 (d, 1H, *J* = 6.9 Hz, H-5), 7.50 (d, 1H, *J* = 9.1 Hz, H-8), 7.25 (s, 1H, H-21), 7.19-7.08 (m, 2H, H-7 & H-23), 6.85-6.74 (m, 2H, H-24 & H-6), 6.65 (t, 1H, *J* = 7.7 Hz, H-22), 5.03 (s, 1H, NH), 4.52 (s, 2H, H-17), 3.03-2.83 (m, 2H, NH & H-11), 1.90-1.82 (m, 2H, H-12a & H-16a), 1.76-1.70 (m, 2H, H-13a & H-15a), 1.65-1.55 (m, 1H, H-14a), 1.30-1.12 (m, 5H, H-12b, H-13b, H-14b, H-15b & H-16b); ^{13}C NMR (101 MHz, $CDCl_3$) δ 144.0 (C-9), 141.6 (C-19), 135.2 (C-3), 129.1 (C-21), 127.8 (C-23), 125.4 (C-2), 123.7 (C-7), 122.6 (C-5), 119.5

(C-20), 117.5 (C-22), 117.3 (C-8), 111.6 (C-6), 111.5 (C-24), 57.2 (C-11), 41.8 (C-17), 34.2 (C-12 & C-16), 25.7 (C-14), 24.9 (C-13 & C-15). IR ν_{max}(cm^{-1}) 2929 (N-H), 1598 (C=C), 741 (C-Cl). HRMS (ES$^+$) calculated for $C_{20}H_{24}N_4Cl$ [M+H]$^+$: 355.1684, found:355.1660.

Appendix 1: ^{1}H NMR and ^{13}C NMR spectral data for selected compounds

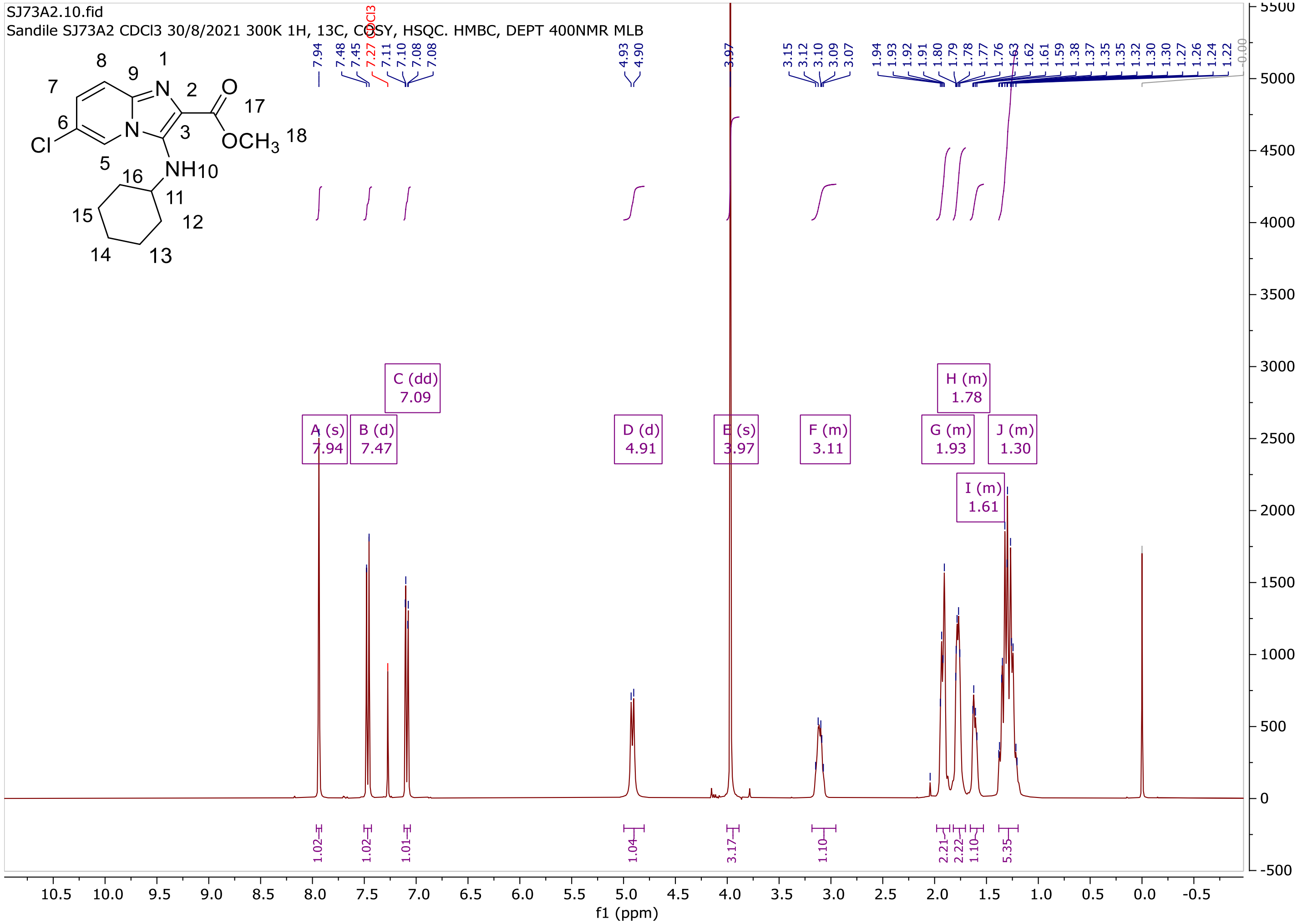

Fig. S1 ^{1}H NMR spectrum for compound **13a**

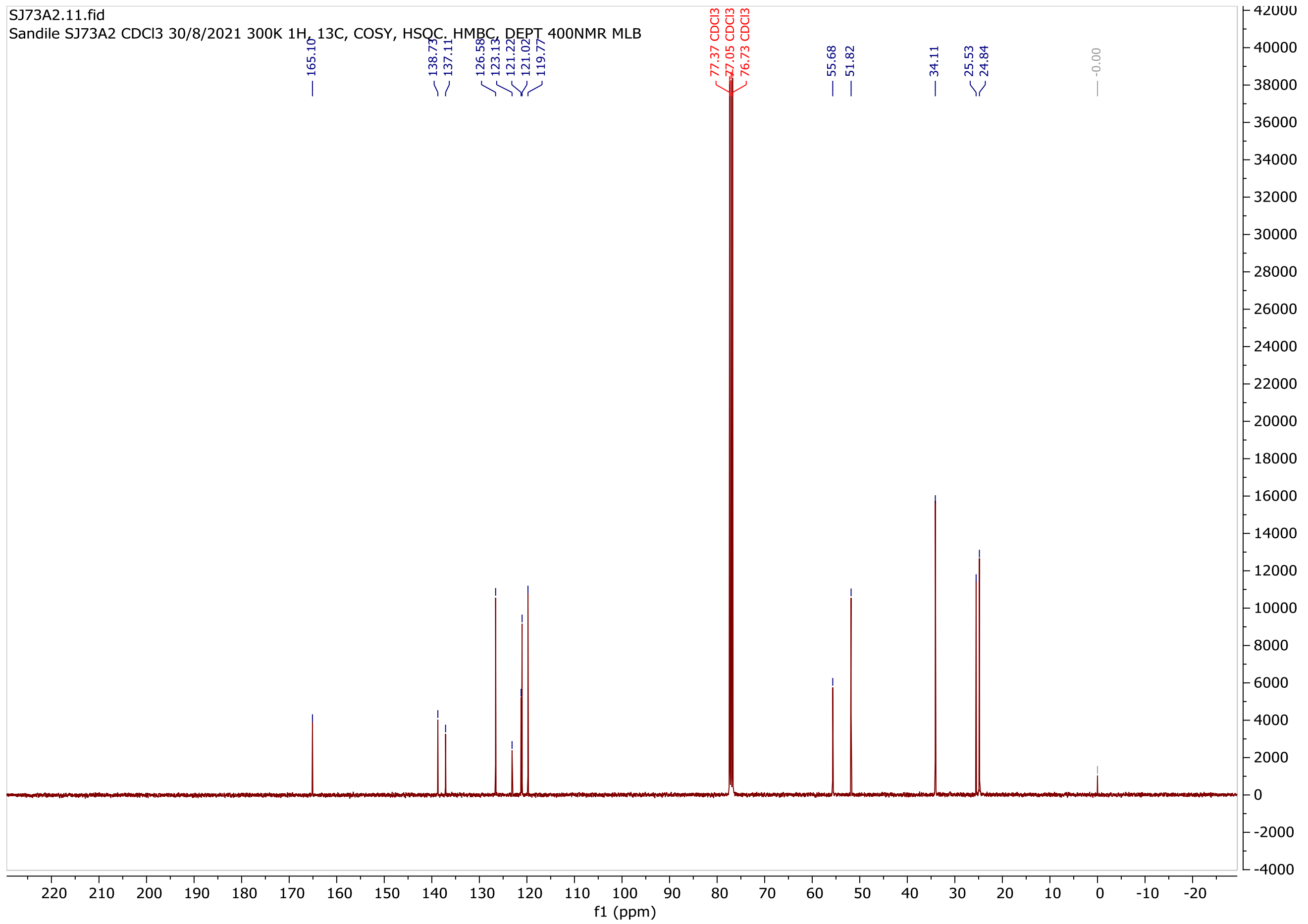

Fig. S2 ^{13}C NMR spectrum for compound **13a**

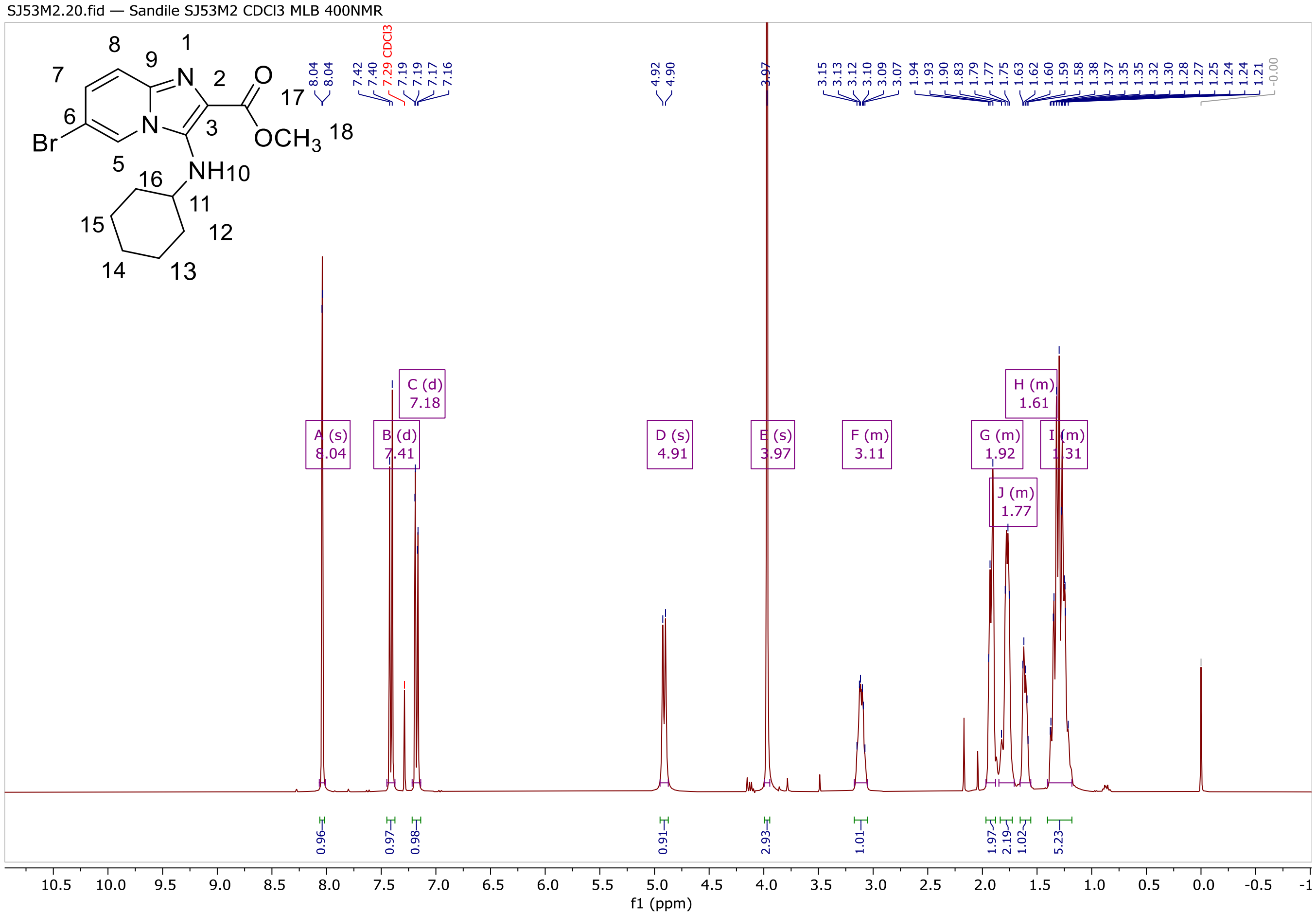

Fig. S3 ^{1}H NMR spectrum for compound **13b**

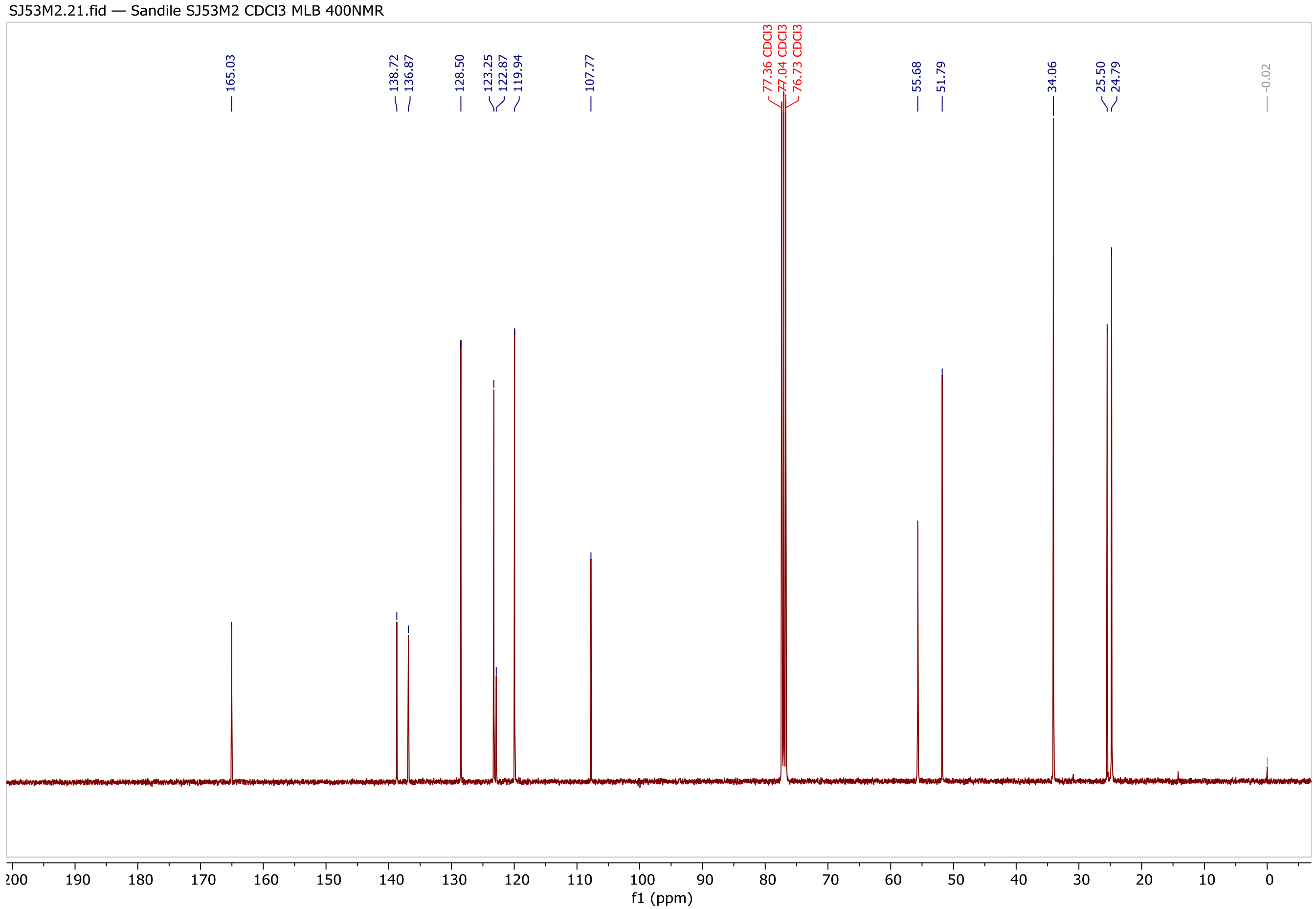

Fig S4. ^{13}C NMR spectrum for compound **13b**

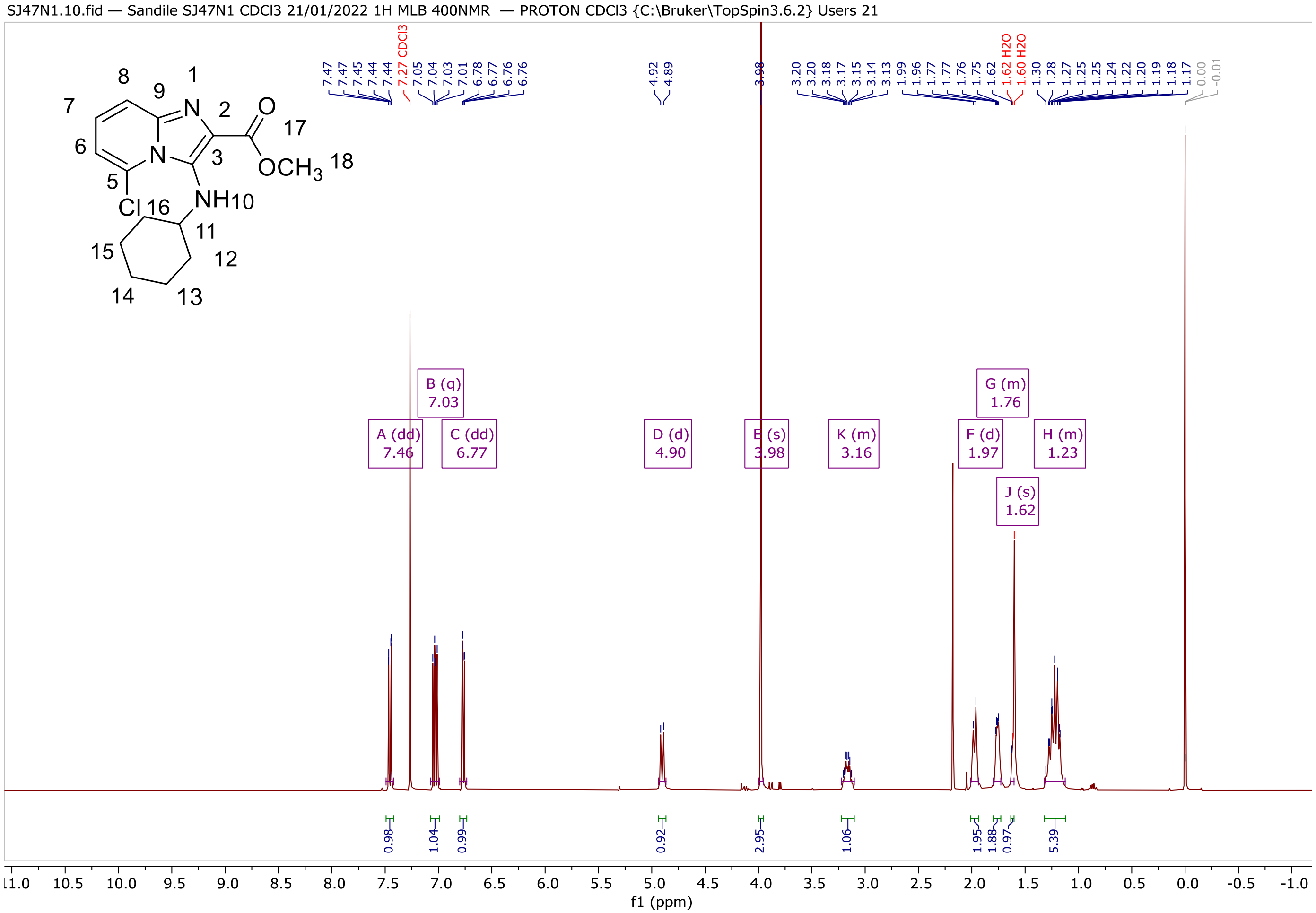

Fig. S5 ^{1}H NMR spectrum for compound **13c**

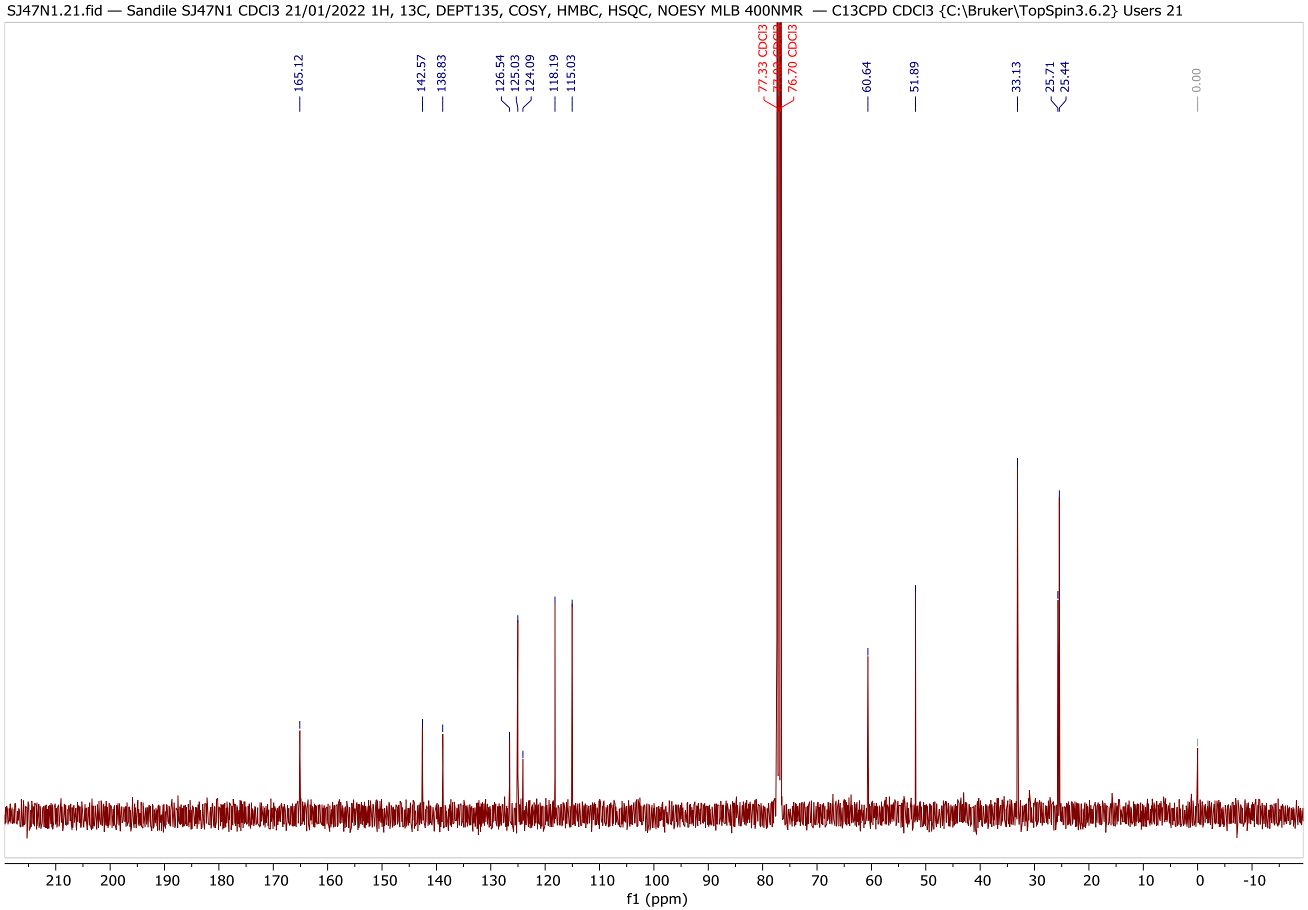

Fig. S5 ^{13}C NMR spectrum for compound **13c**

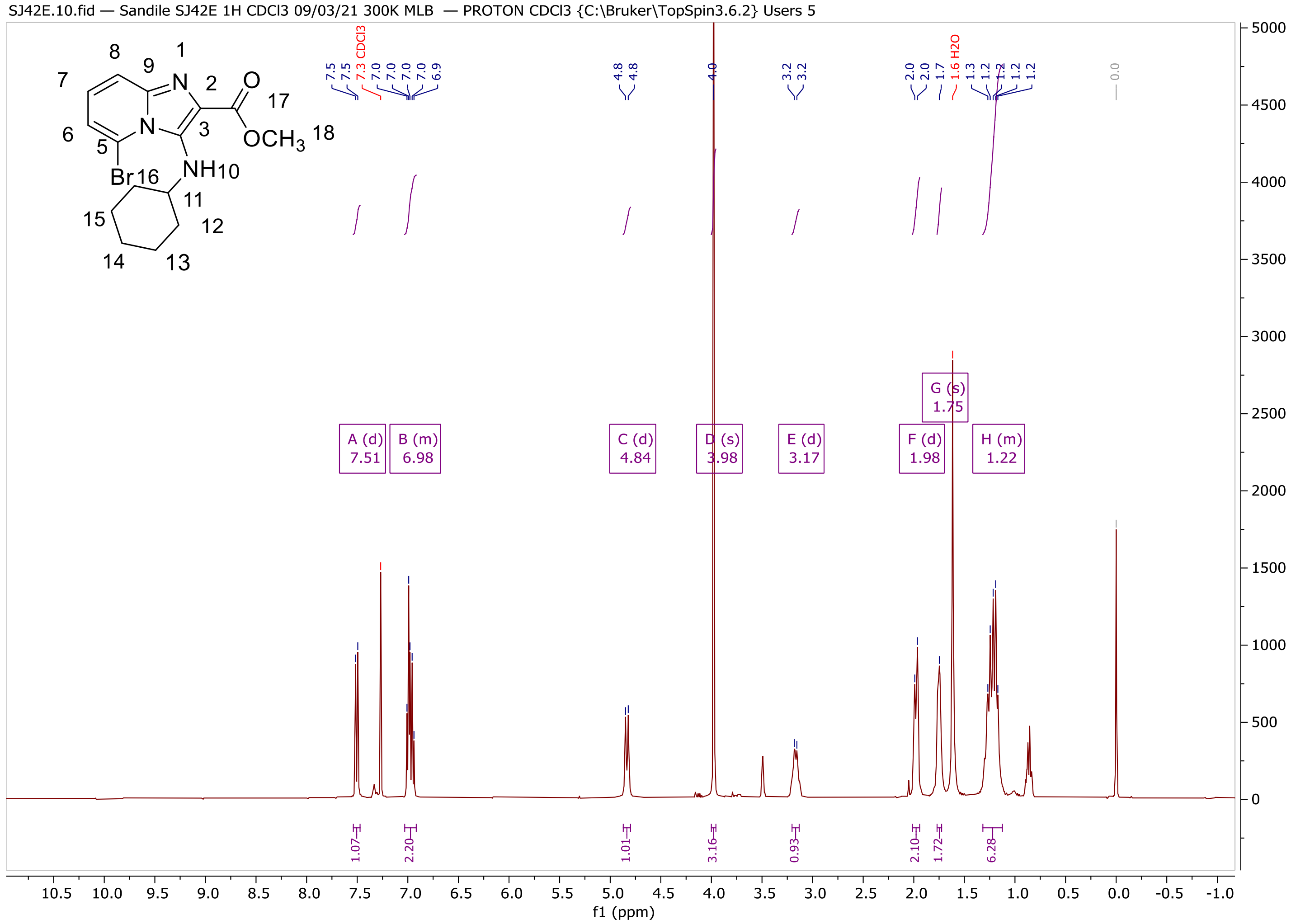

Fig. S6 ^{1}H NMR spectrum for compound **13d**

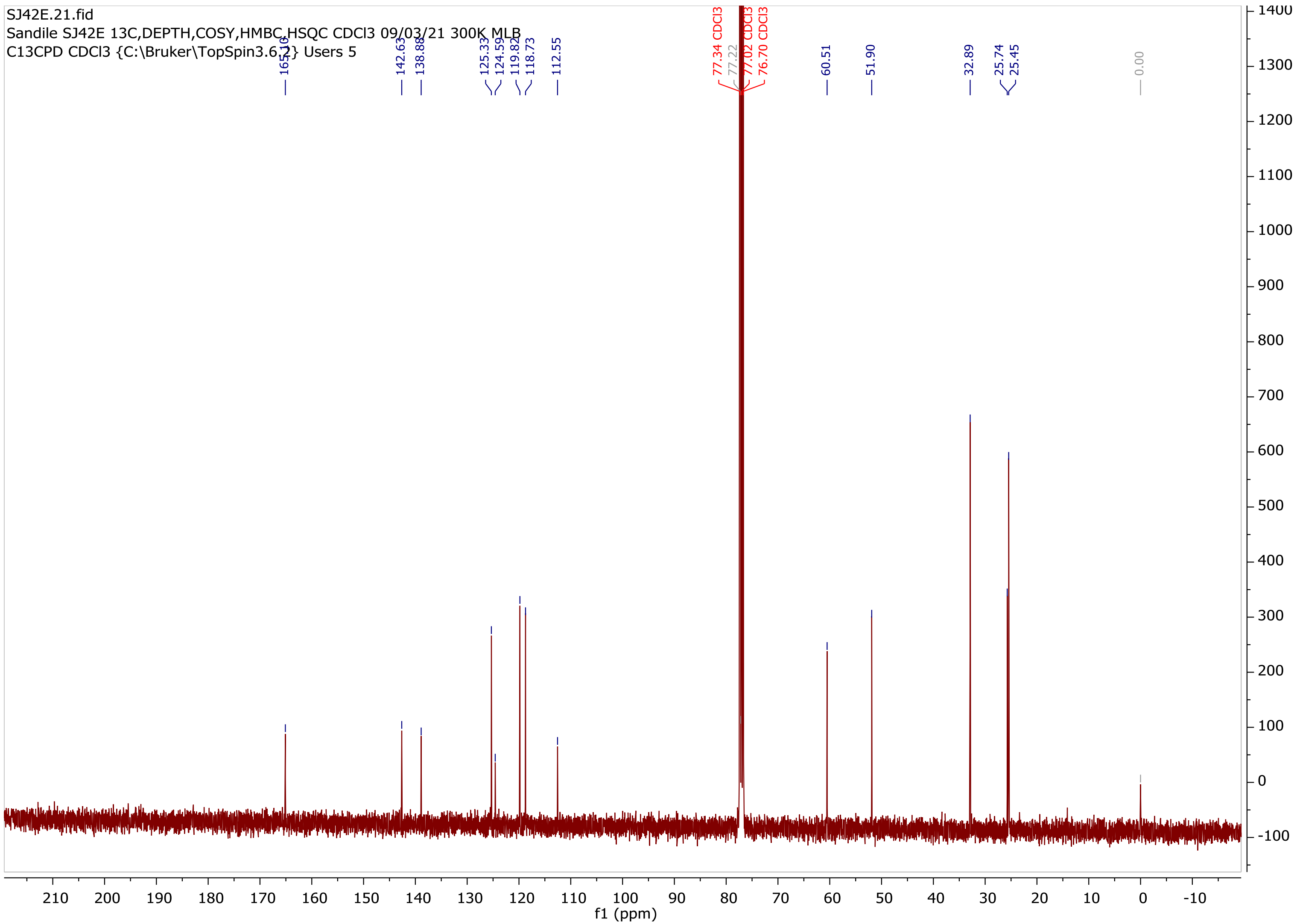

Fig. S7 ^{13}C NMR spectrum for compound **13d**

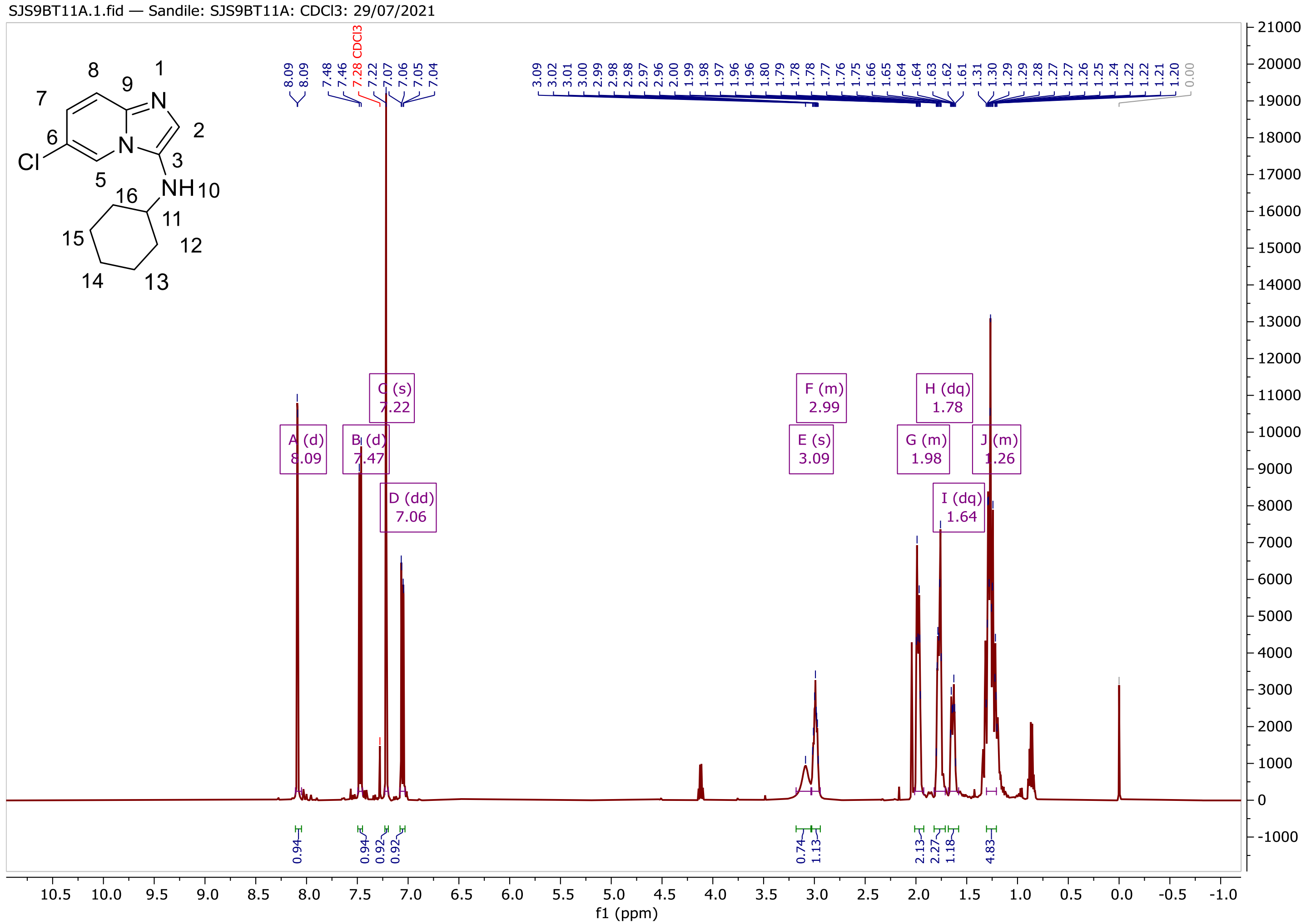

Fig. S8 ^{1}H NMR spectrum for compound **12a**

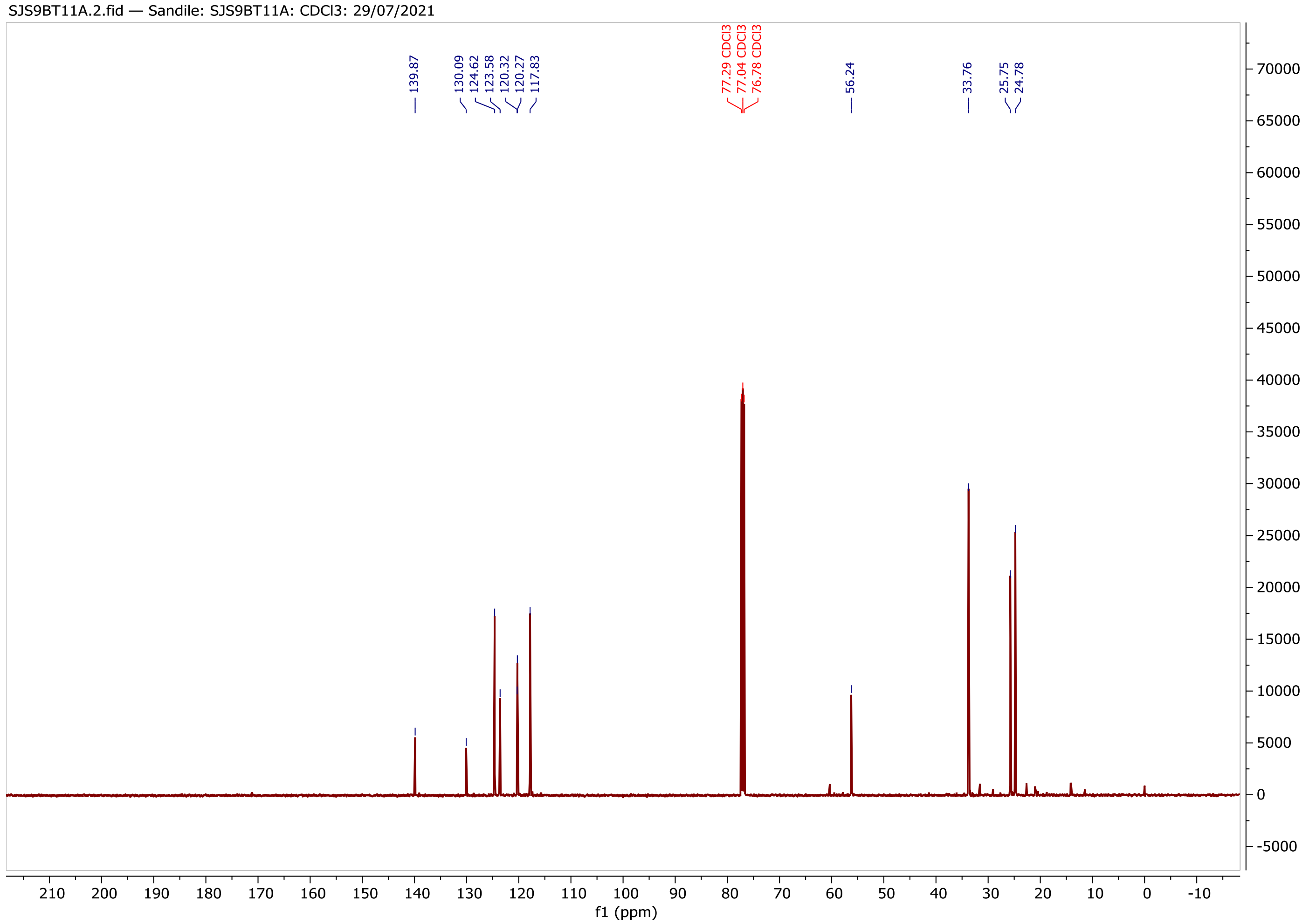

Fig. S9 ^{13}C NMR spectrum for compound **12a**

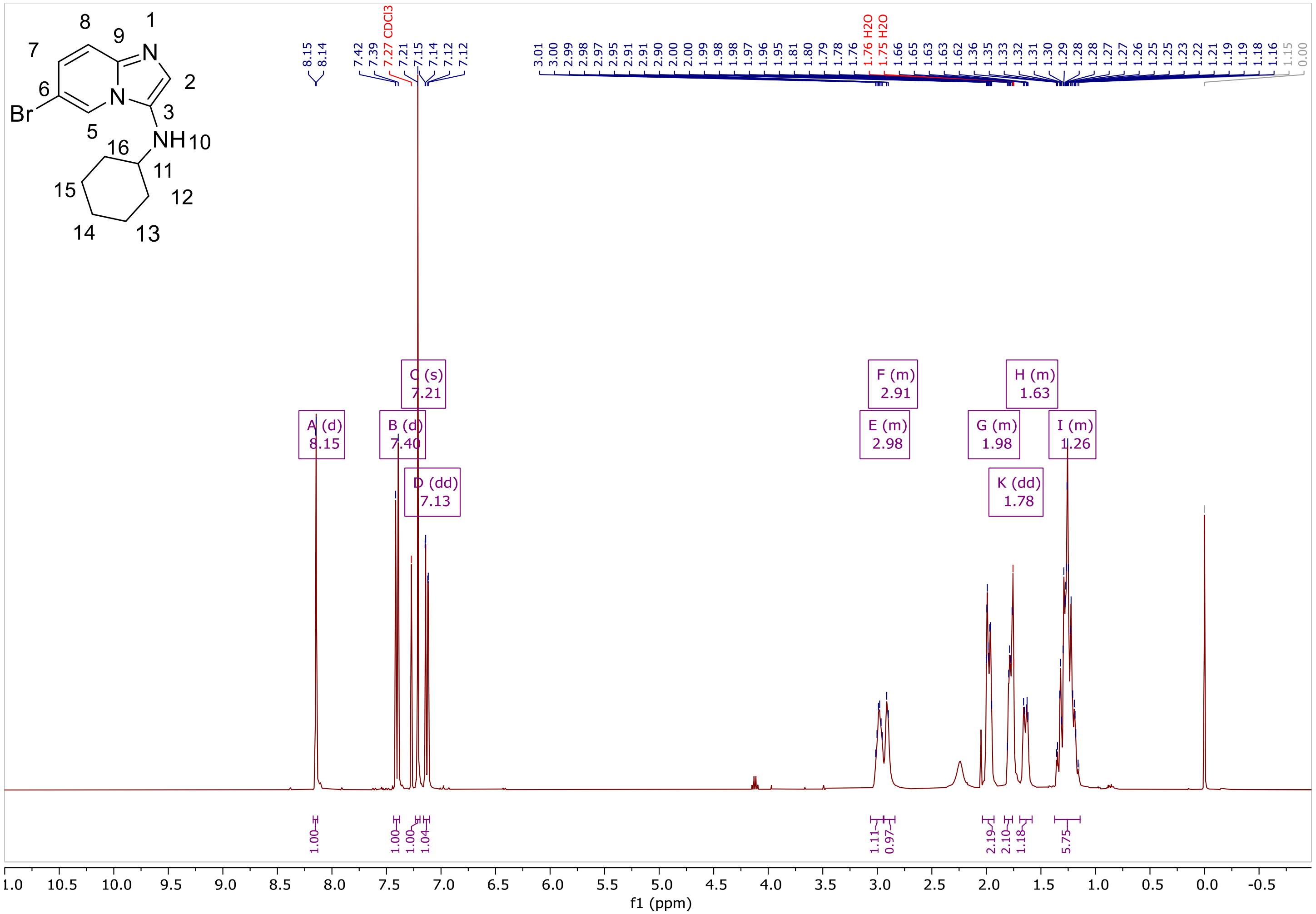

Fig. S10 ¹H NMR spectrum for compound **12b**

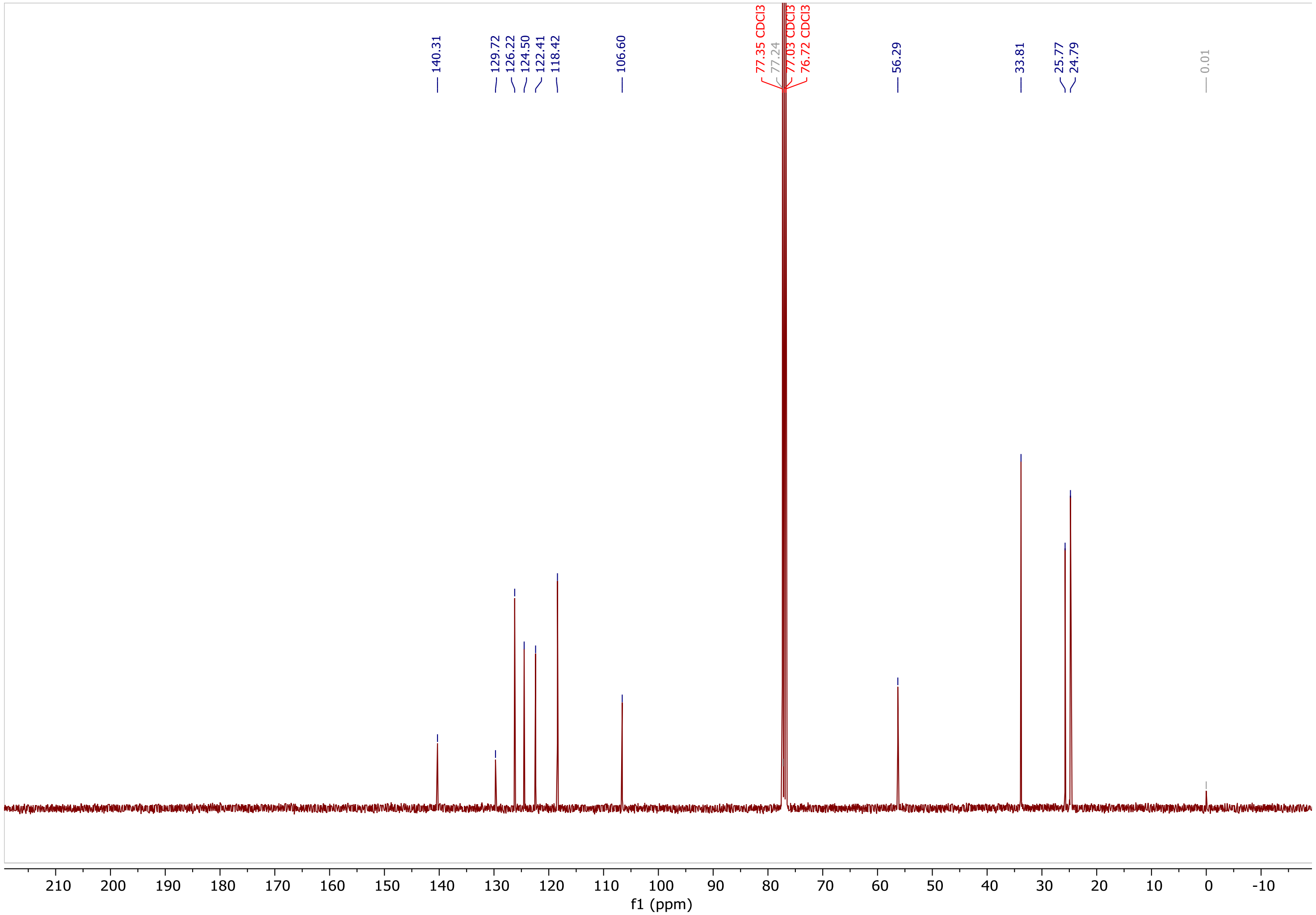

Fig. S11 ^{13}C NMR spectrum for compound **12b**

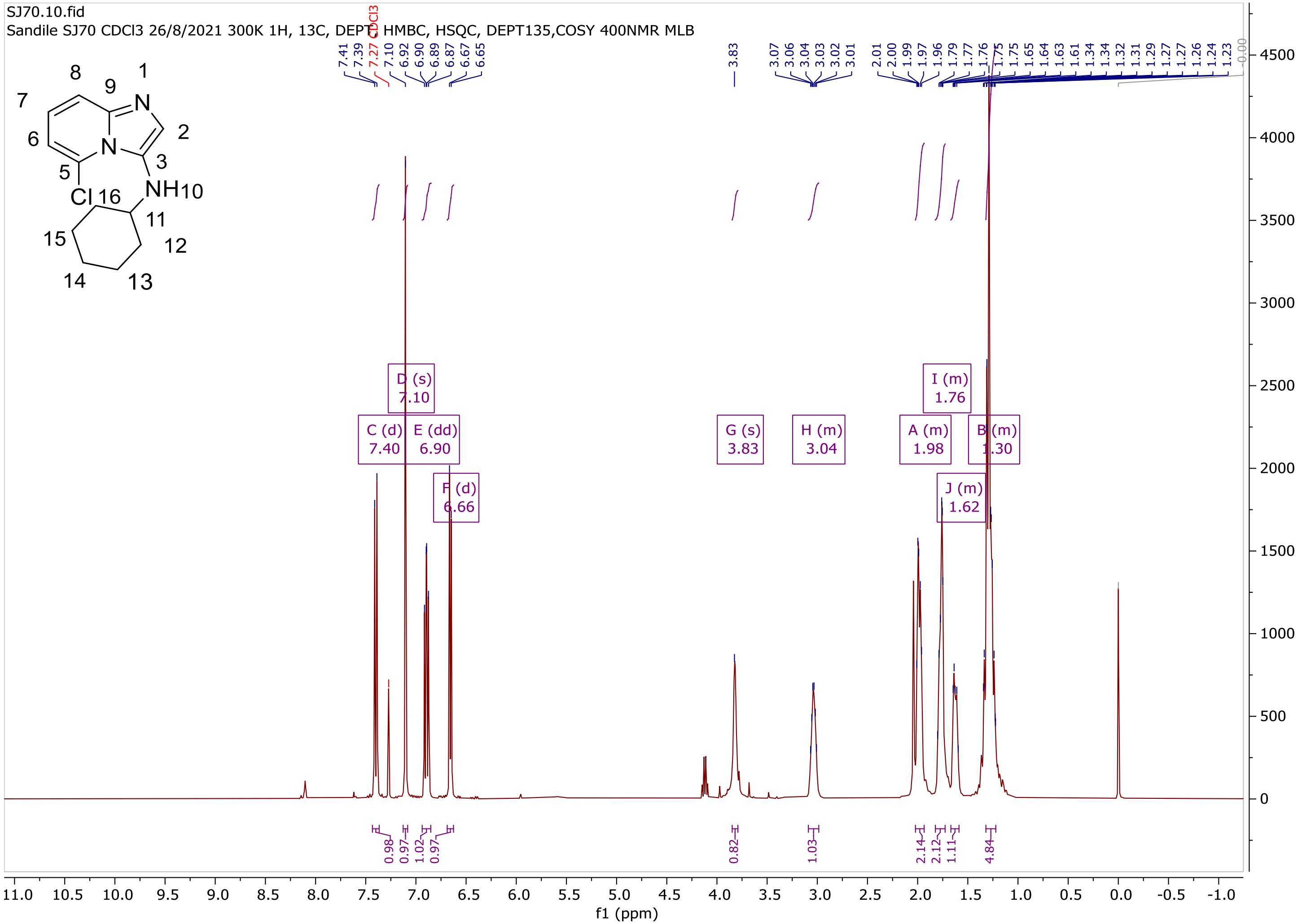

Fig. S12 ^{1}H NMR spectrum for compound **12c**

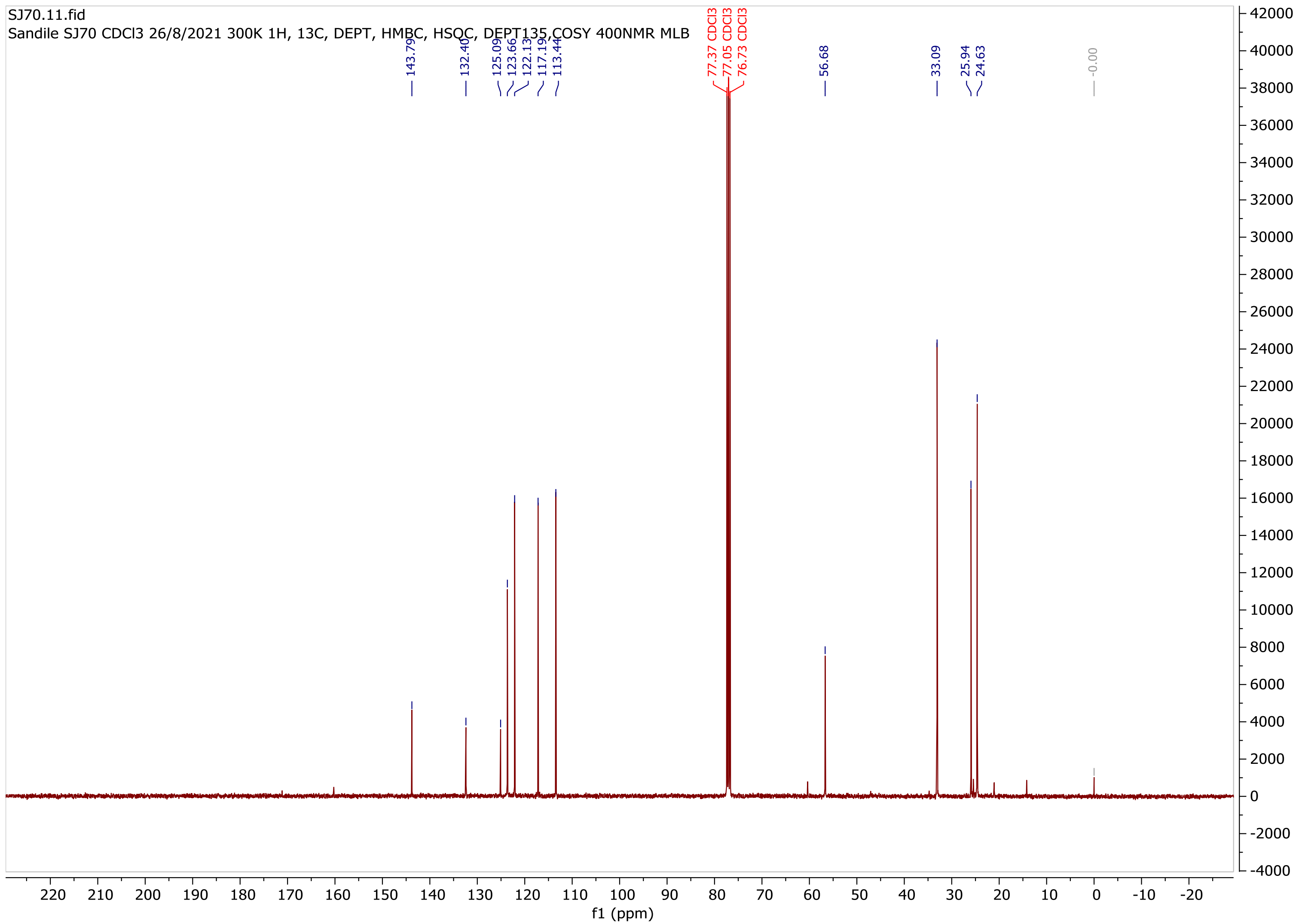

Fig. S13 ^{13}C NMR spectrum for compound **12c**

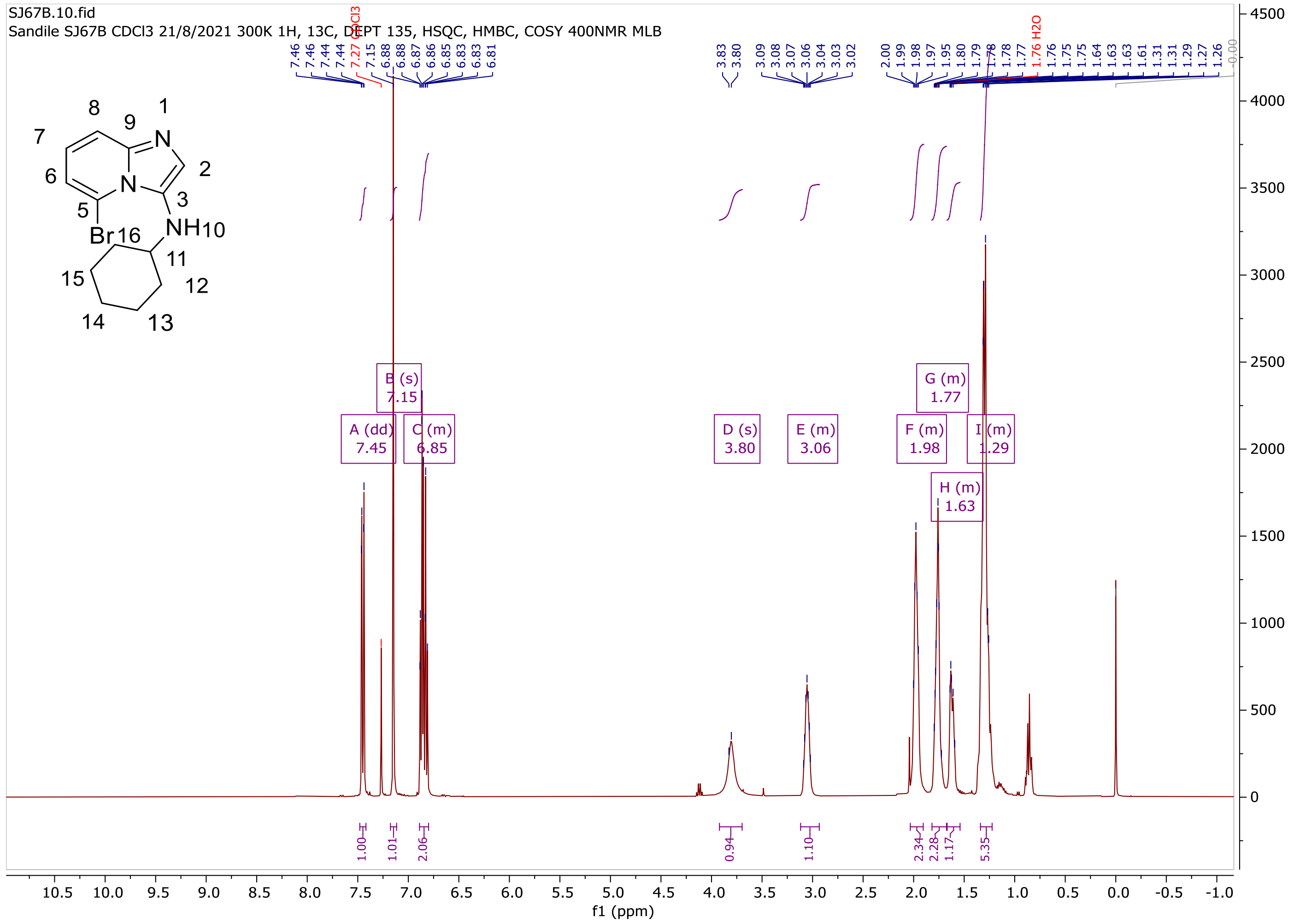

Fig. S14 ^{1}H NMR spectrum for compound **12d**

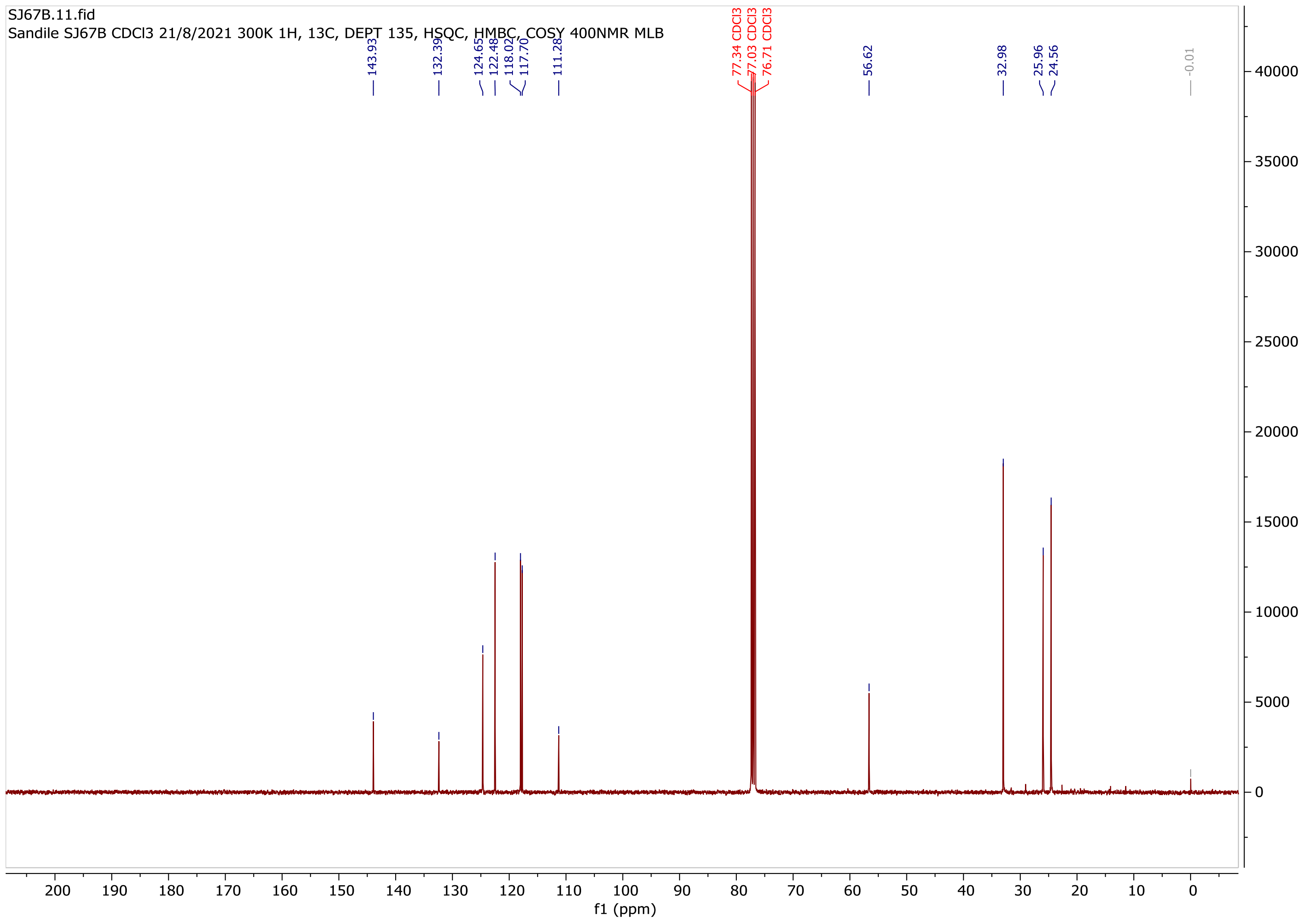

Fig. S15 ^{13}C NMR spectrum for compound **12d**

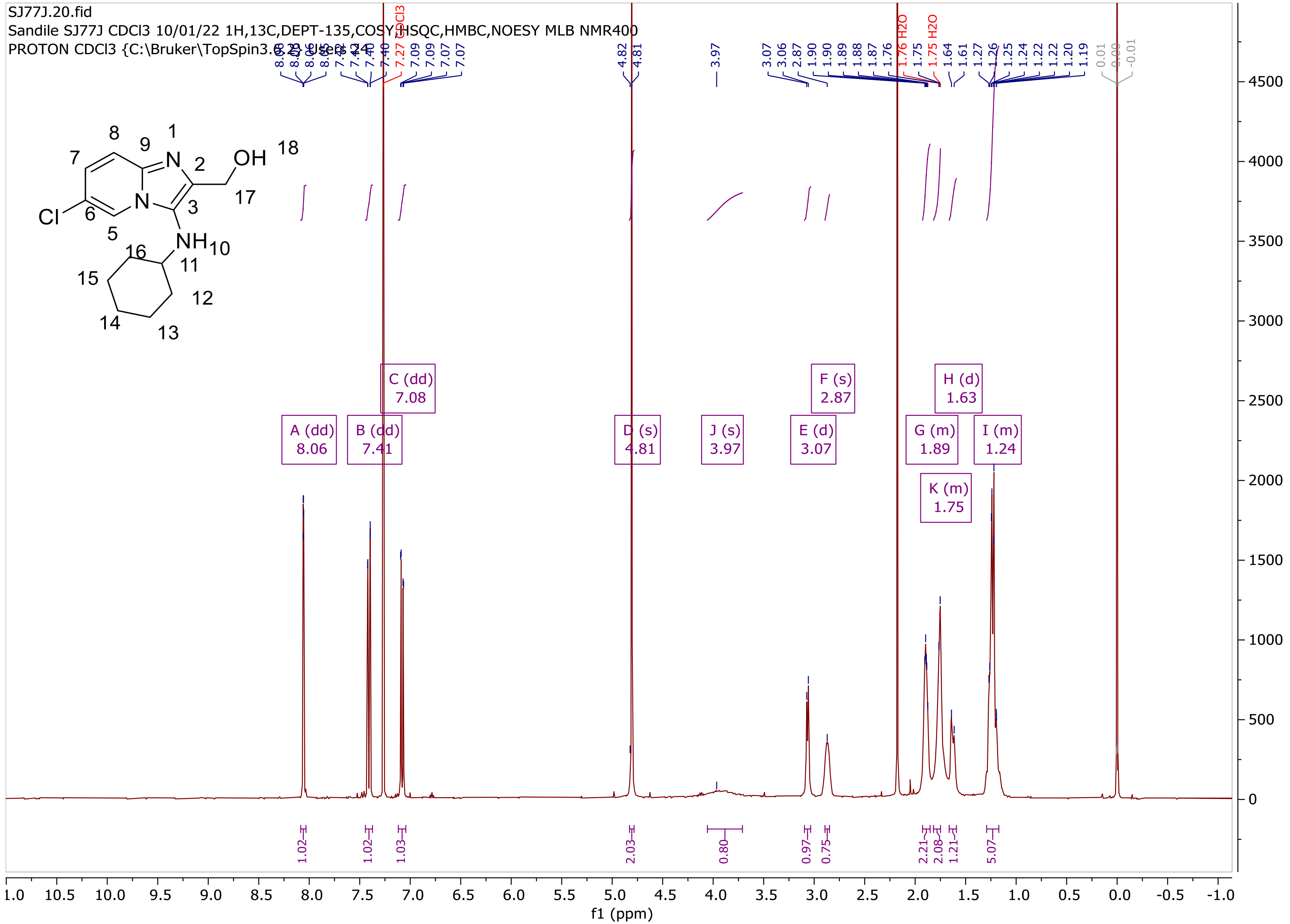

Fig. S16 ^{1}H NMR spectrum for compound **17a**

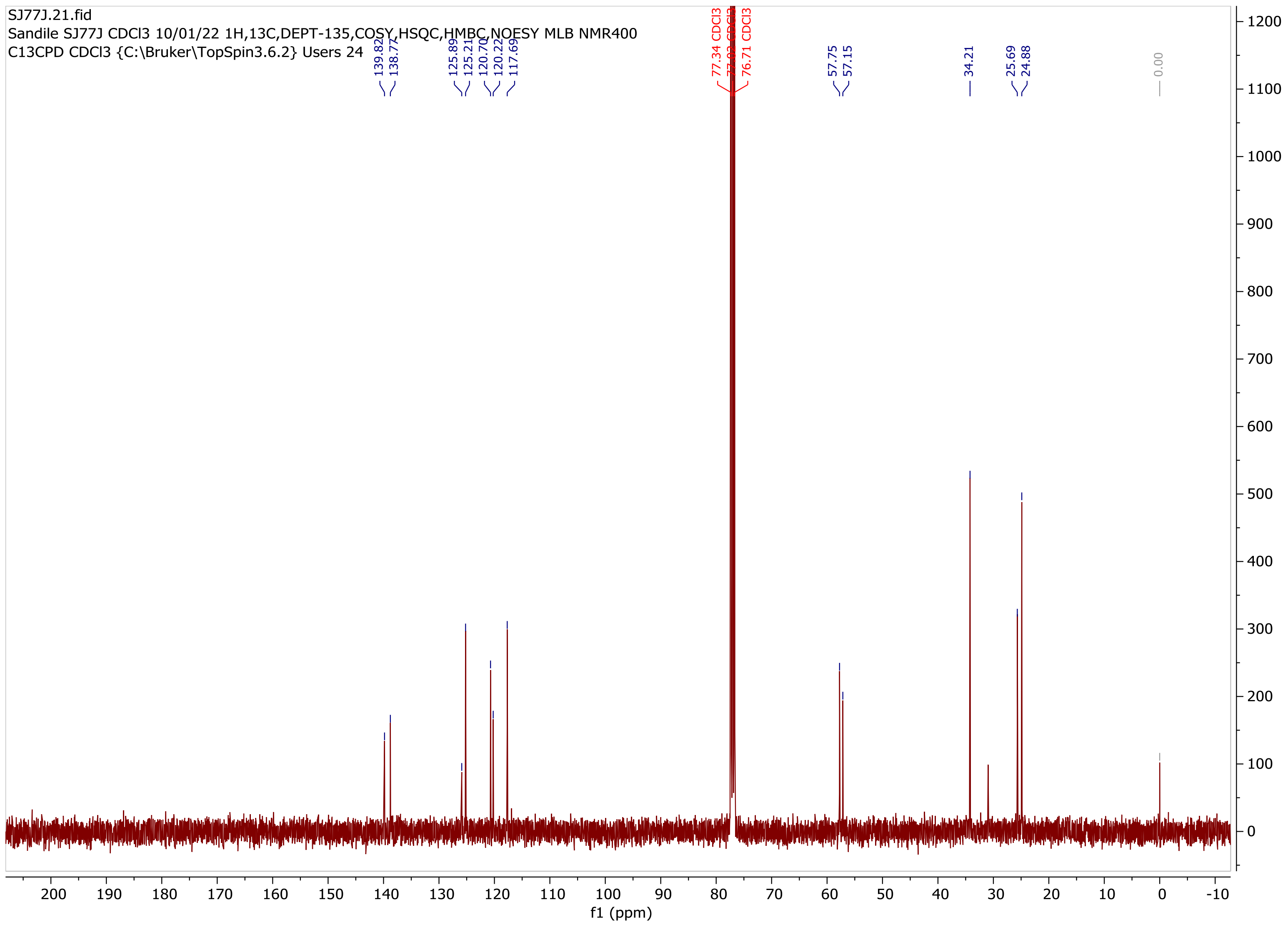

Fig. S17 ^{13}C NMR spectrum for compound **17a**

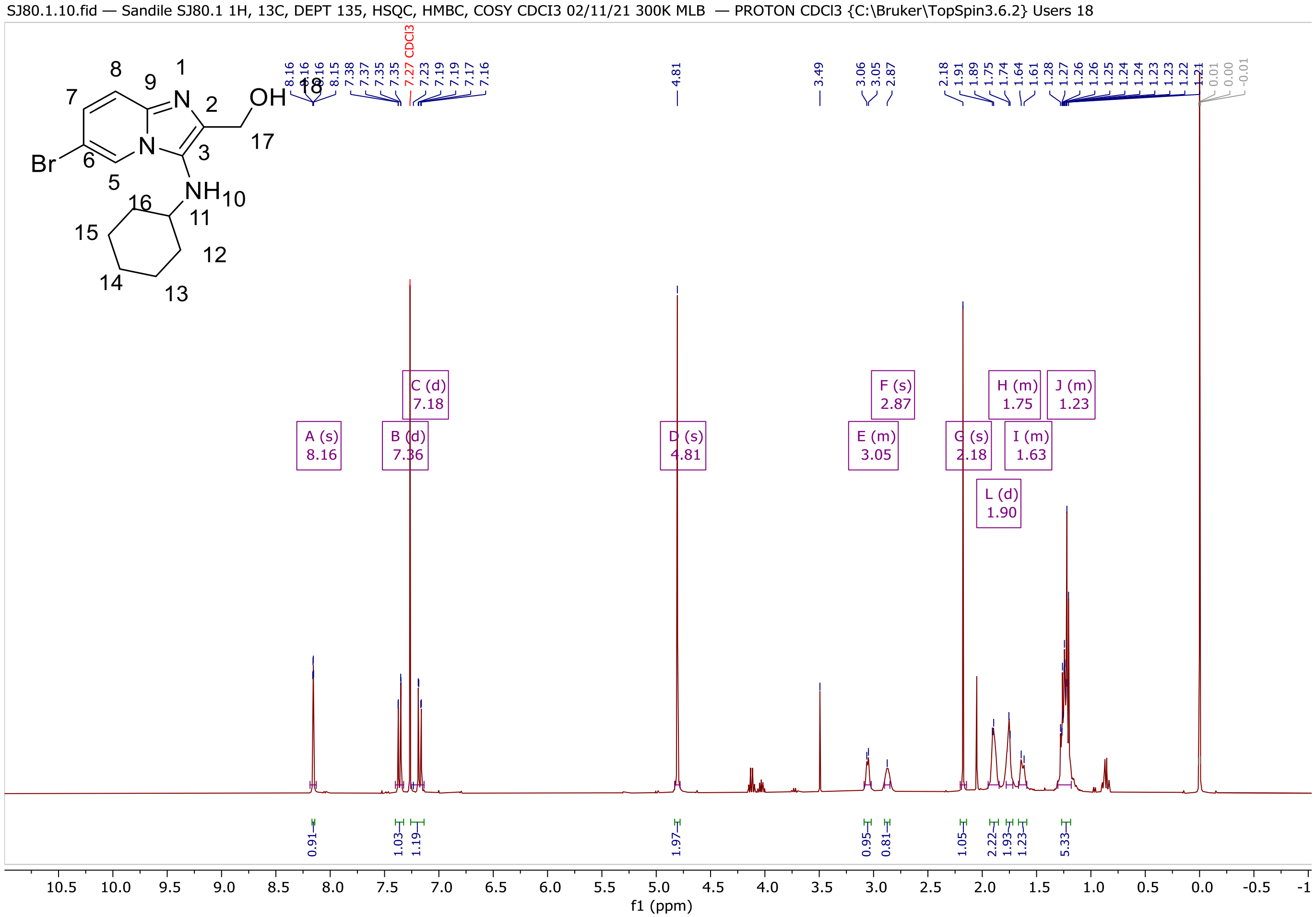

Fig. S18 ^{1}H NMR spectrum for compound **17b**

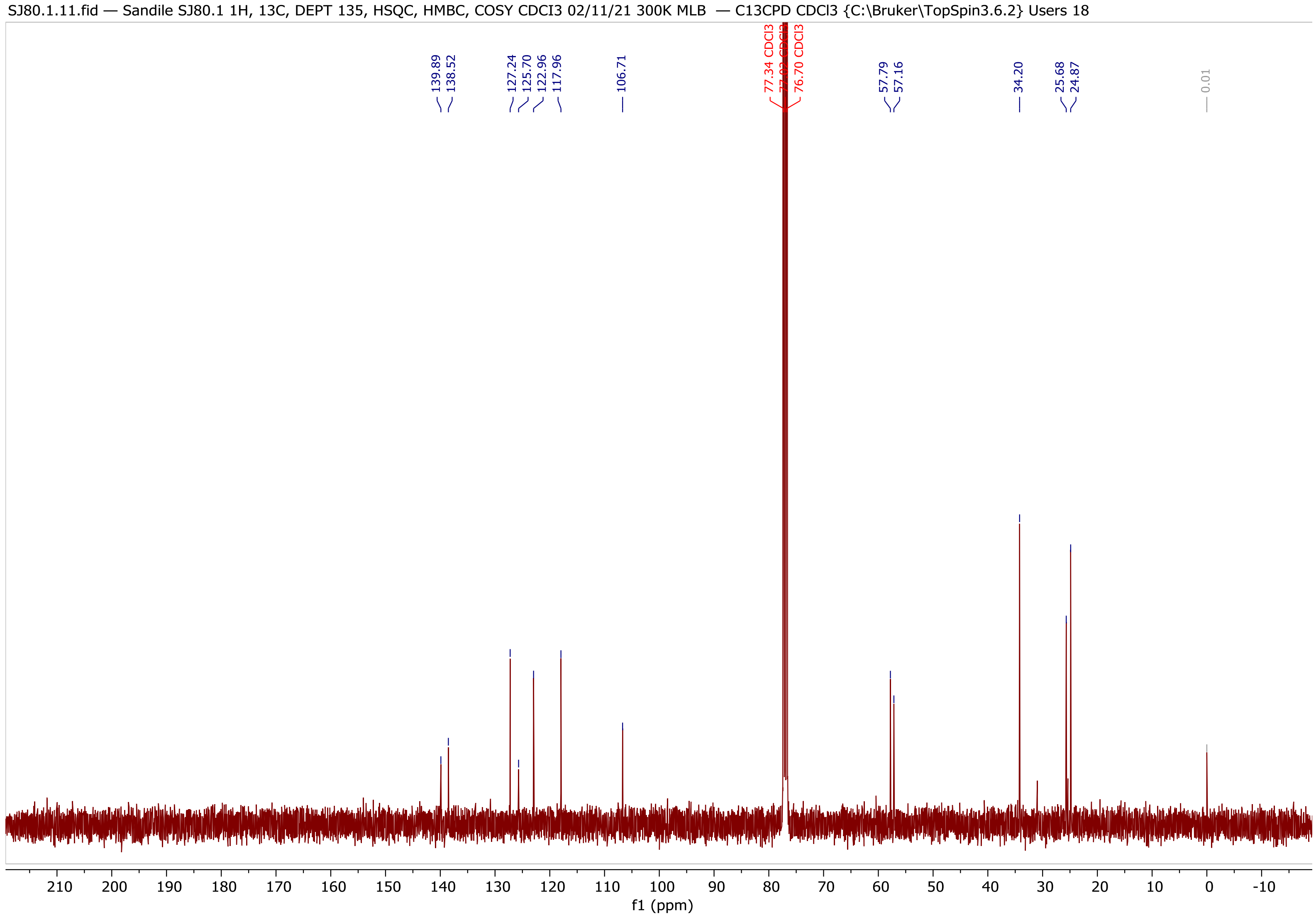

Fig. S19 ^{13}C NMR spectrum for compound **17b**

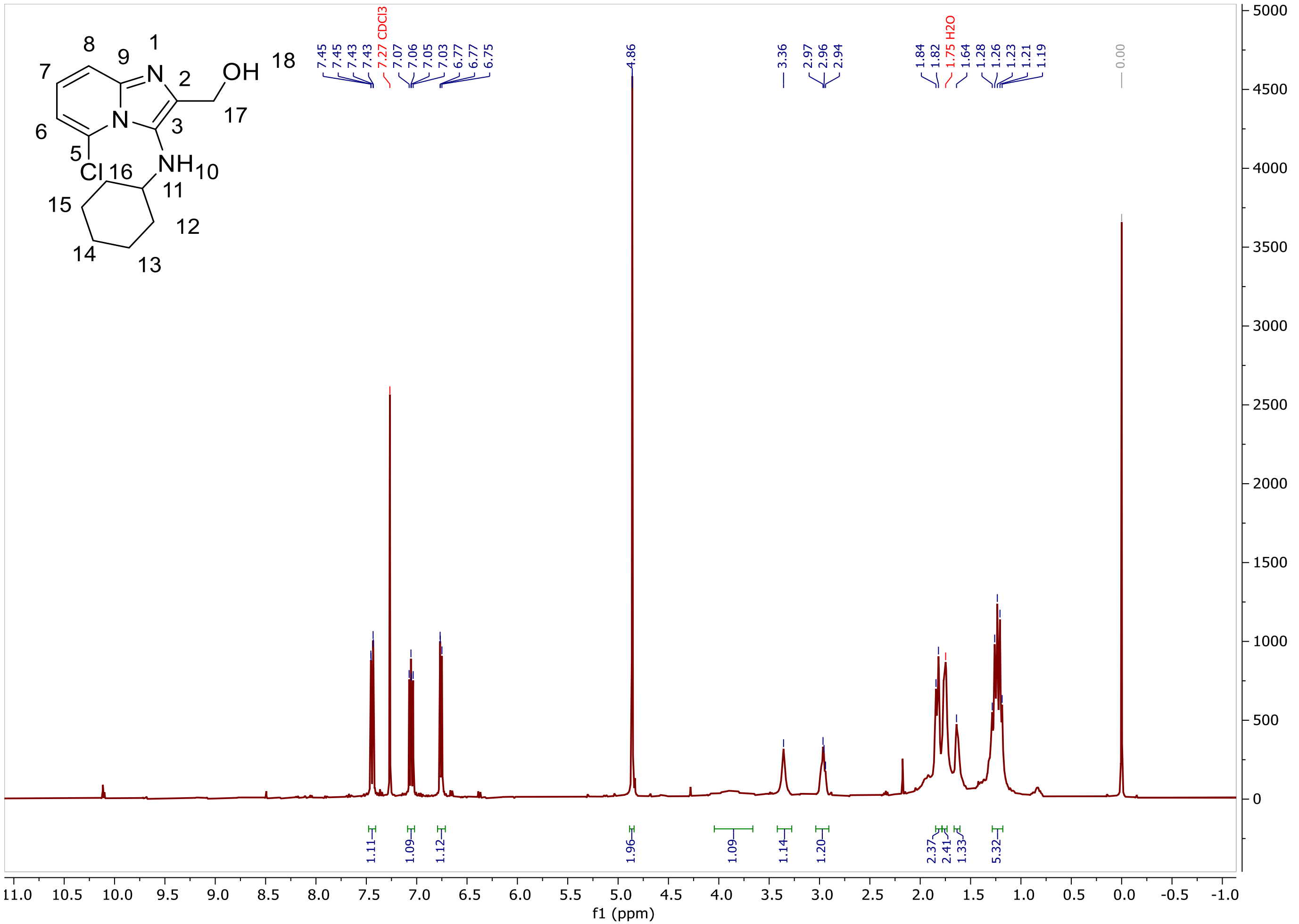

Fig. S20 ^{1}H NMR spectrum for compound **17c**

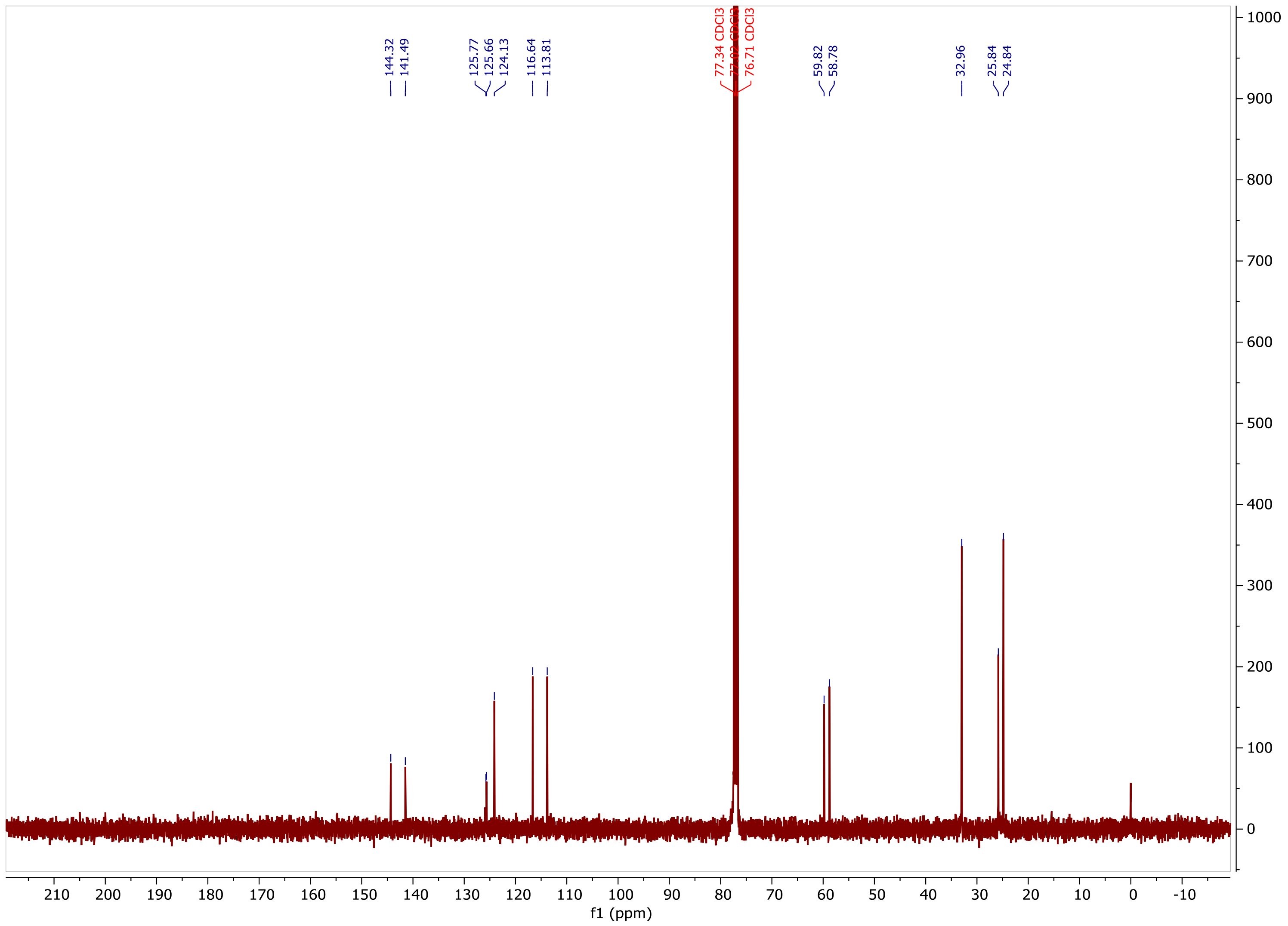

Fig. S21 ^{13}C NMR spectrum for compound **17c**

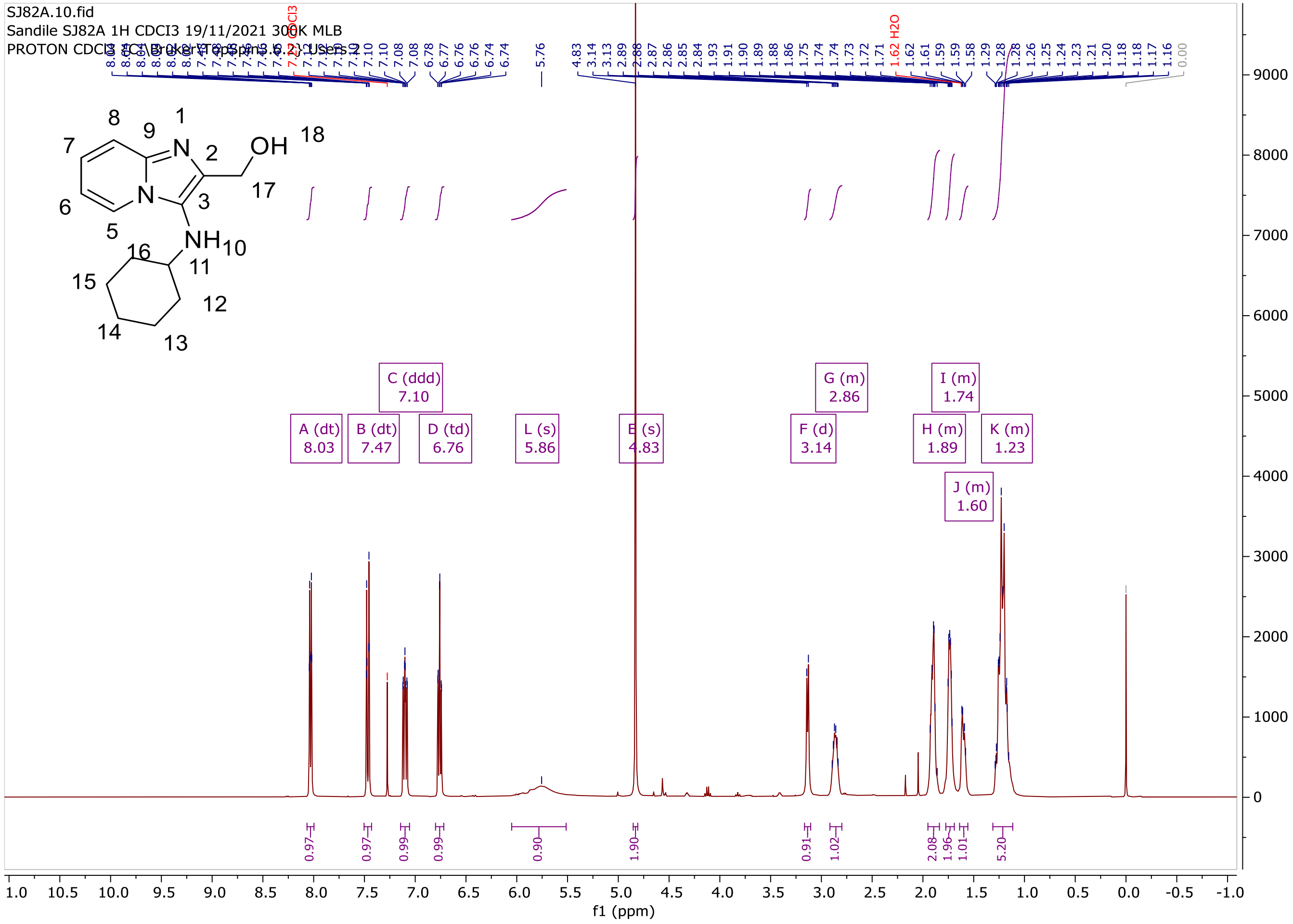

Fig. S22 ^{1}H NMR spectrum for compound **17d**

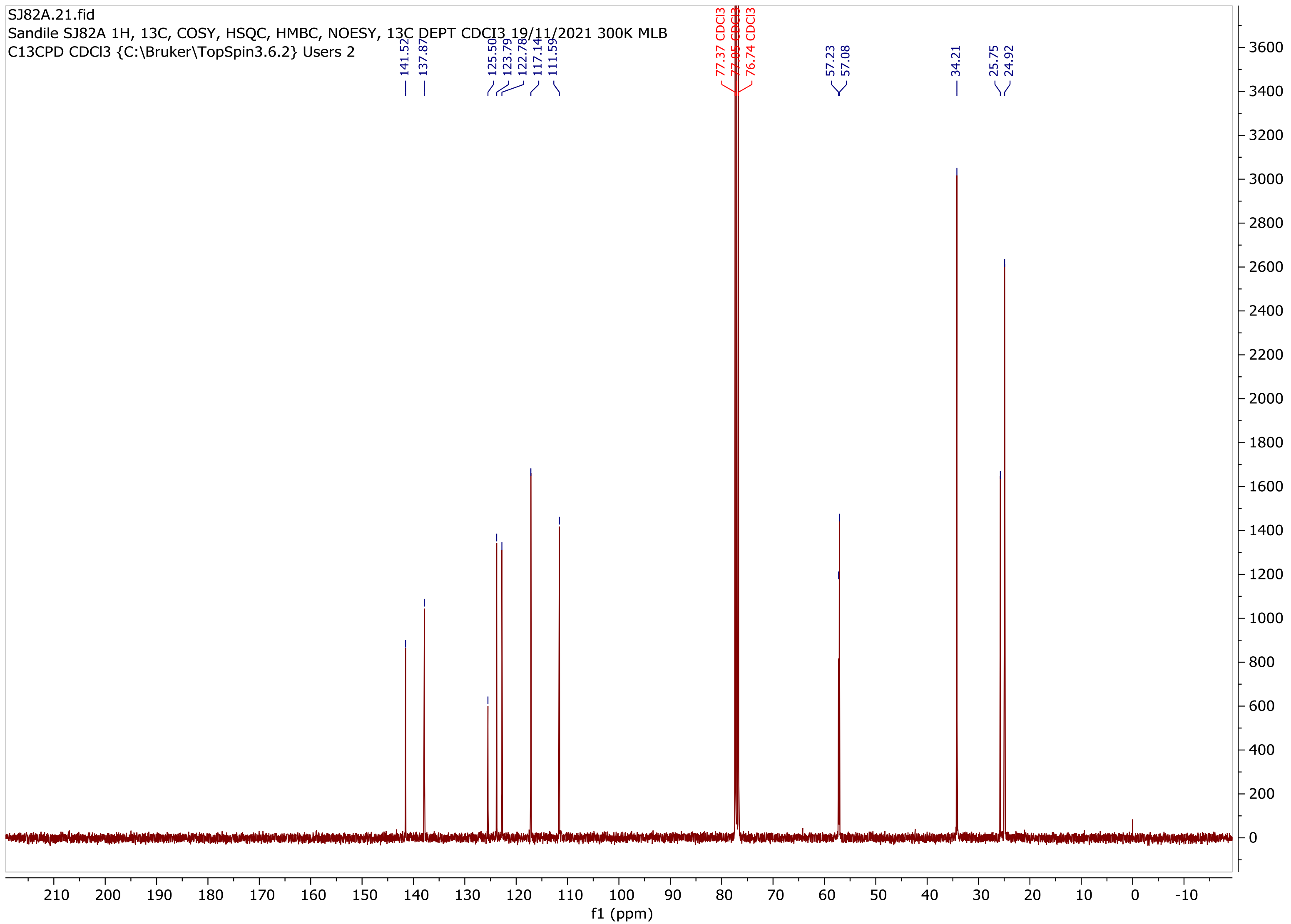

Fig. S23 ^{13}C NMR spectrum for compound **17d**

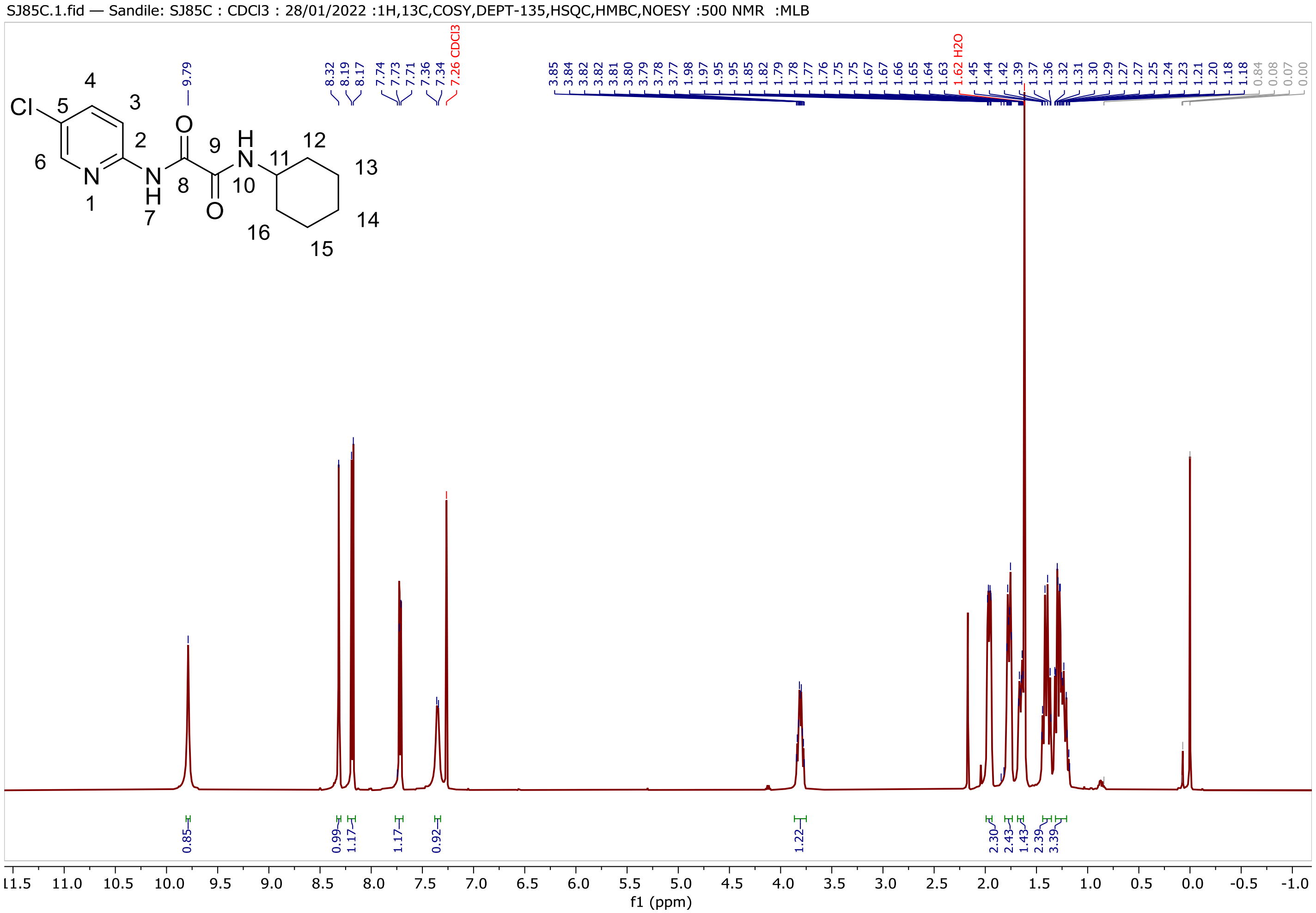

Fig. S24 ^{1}H NMR spectrum for compound **18a**

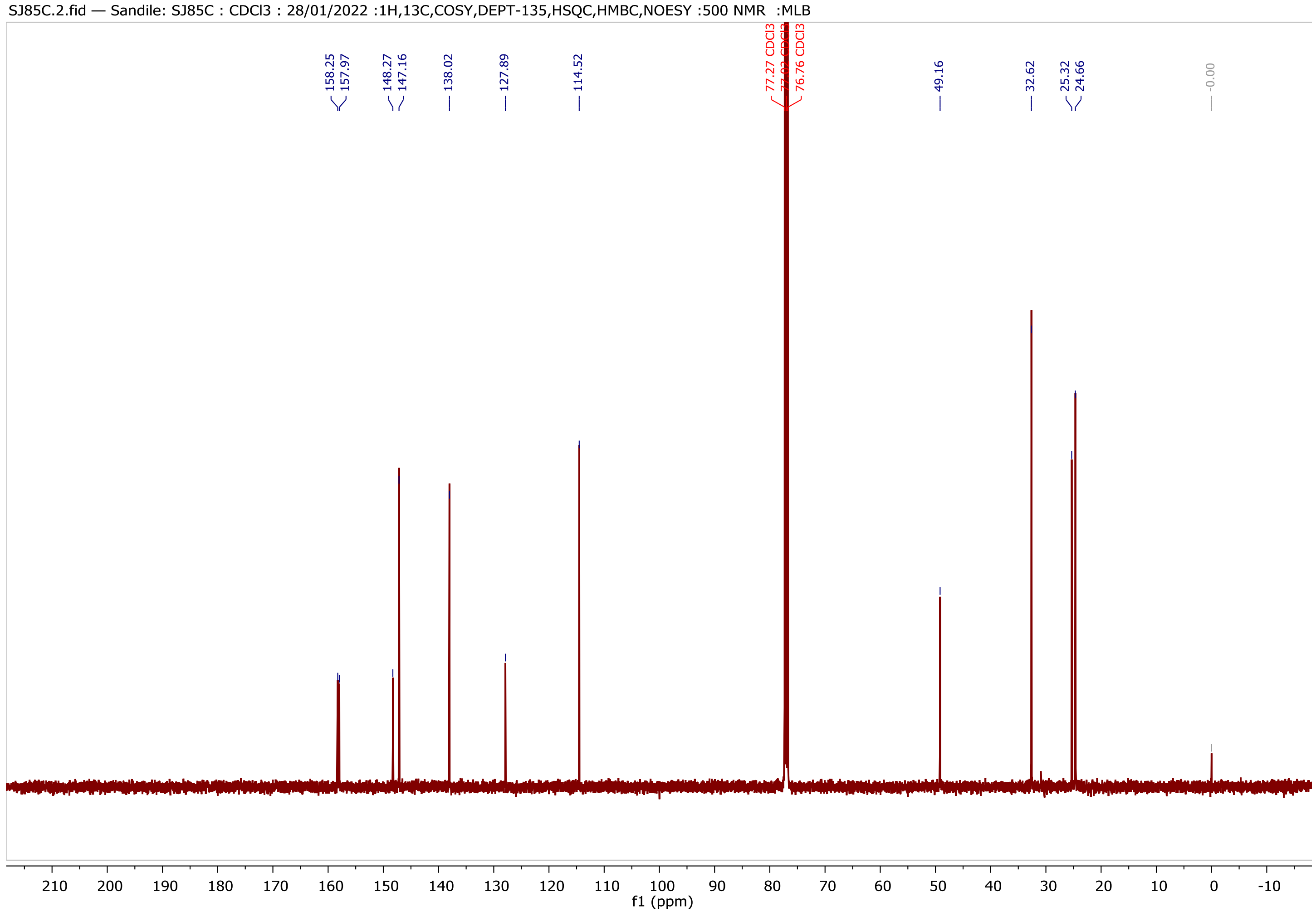

Fig. S25 ^{13}C NMR spectrum for compound **18a**

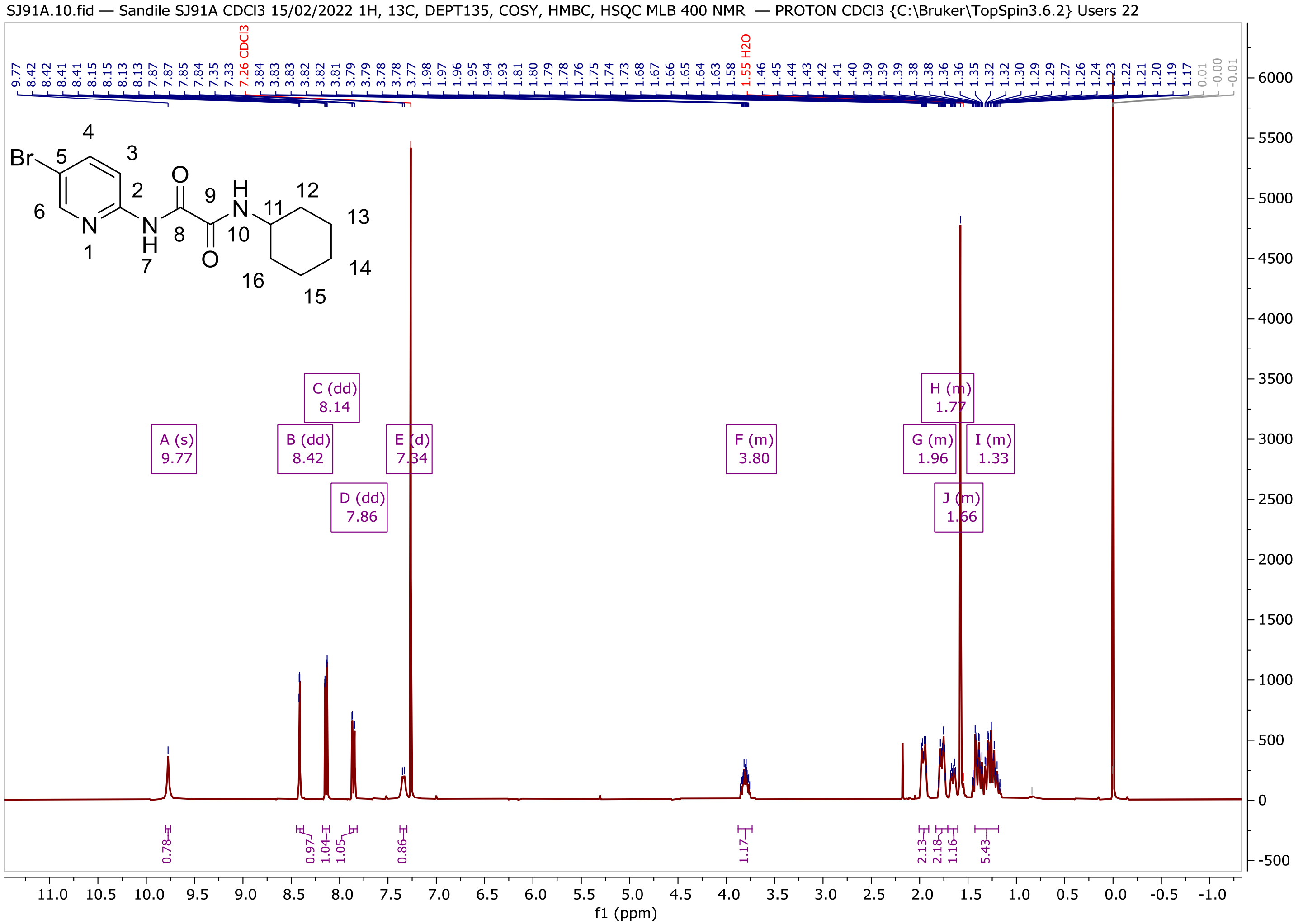

Fig. S26 ^{1}H NMR spectrum for compound **18b**

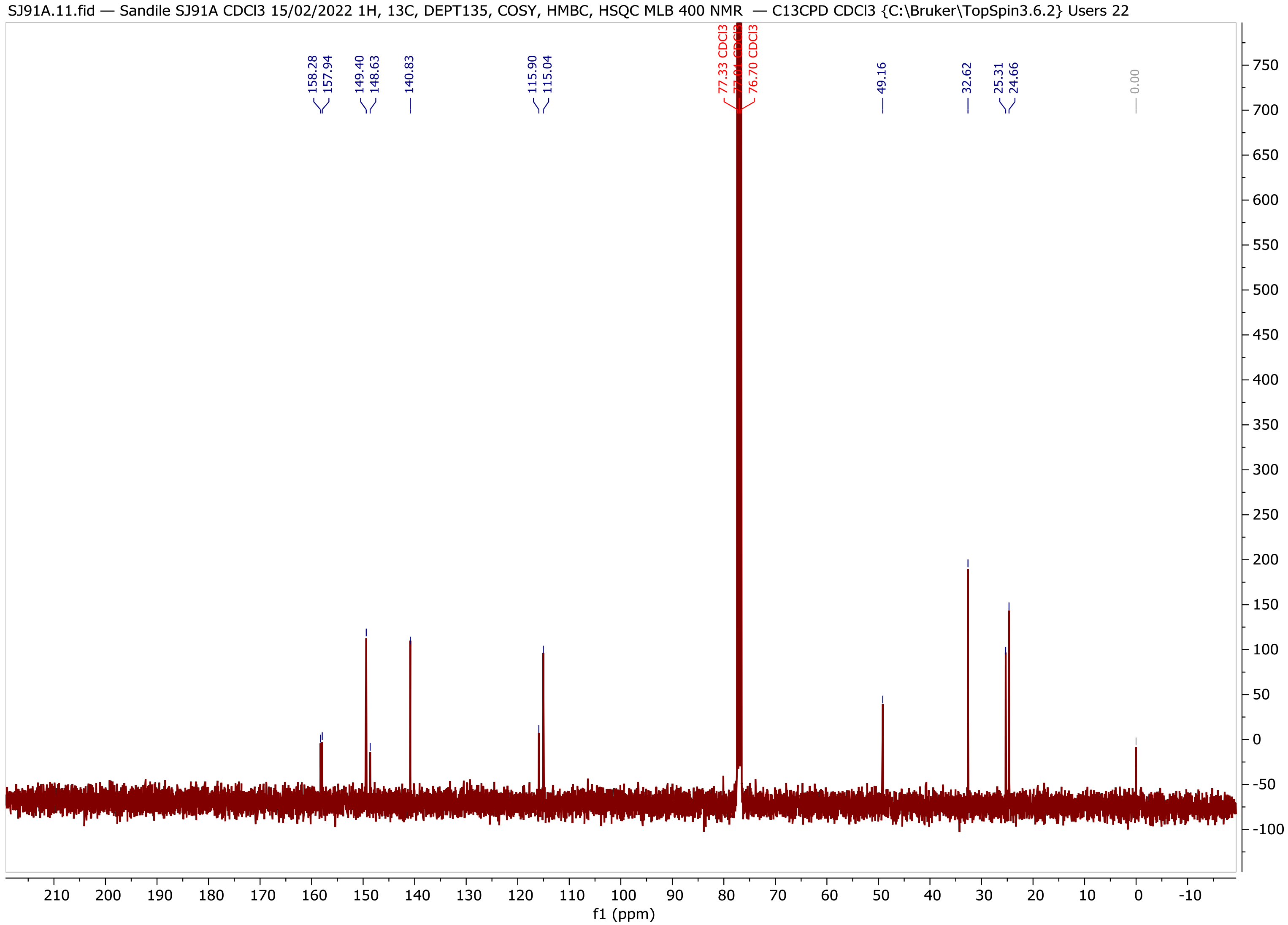

Fig. S27 ^{13}C NMR spectrum for compound **18b**

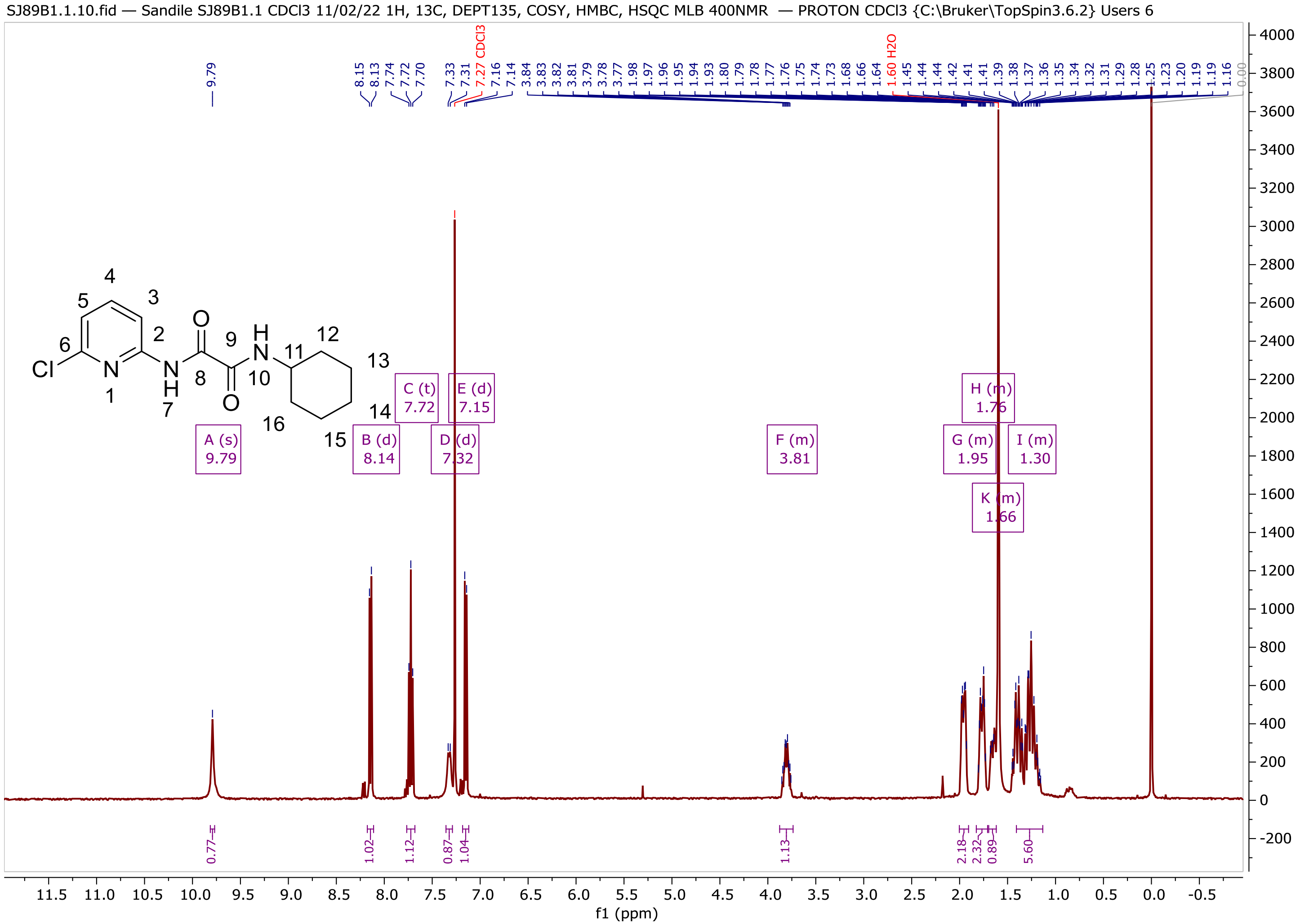

Fig. S28 ^{1}H NMR spectrum for compound **18c**

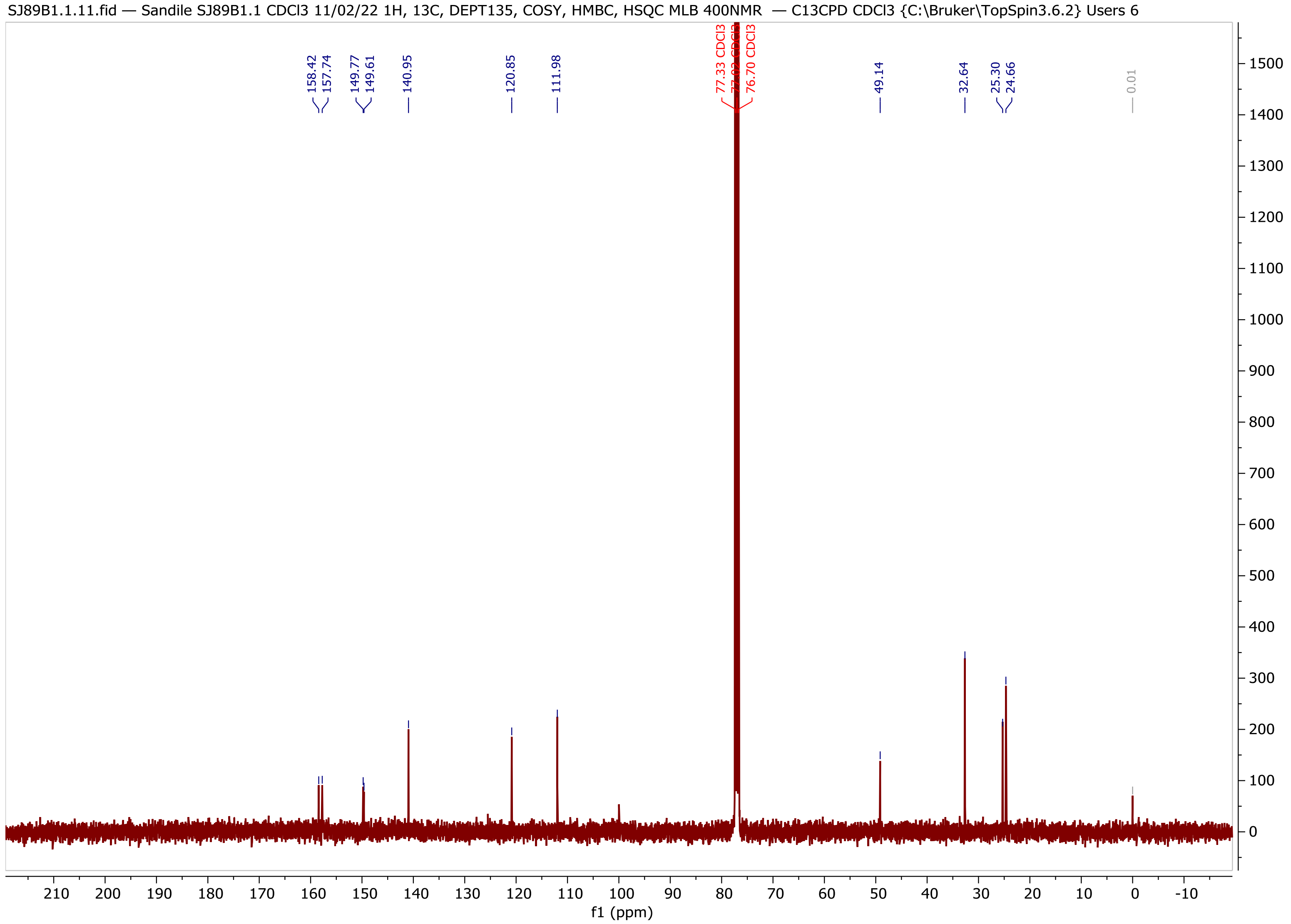

Fig. S29 ^{13}C NMR spectrum for compound **18c**

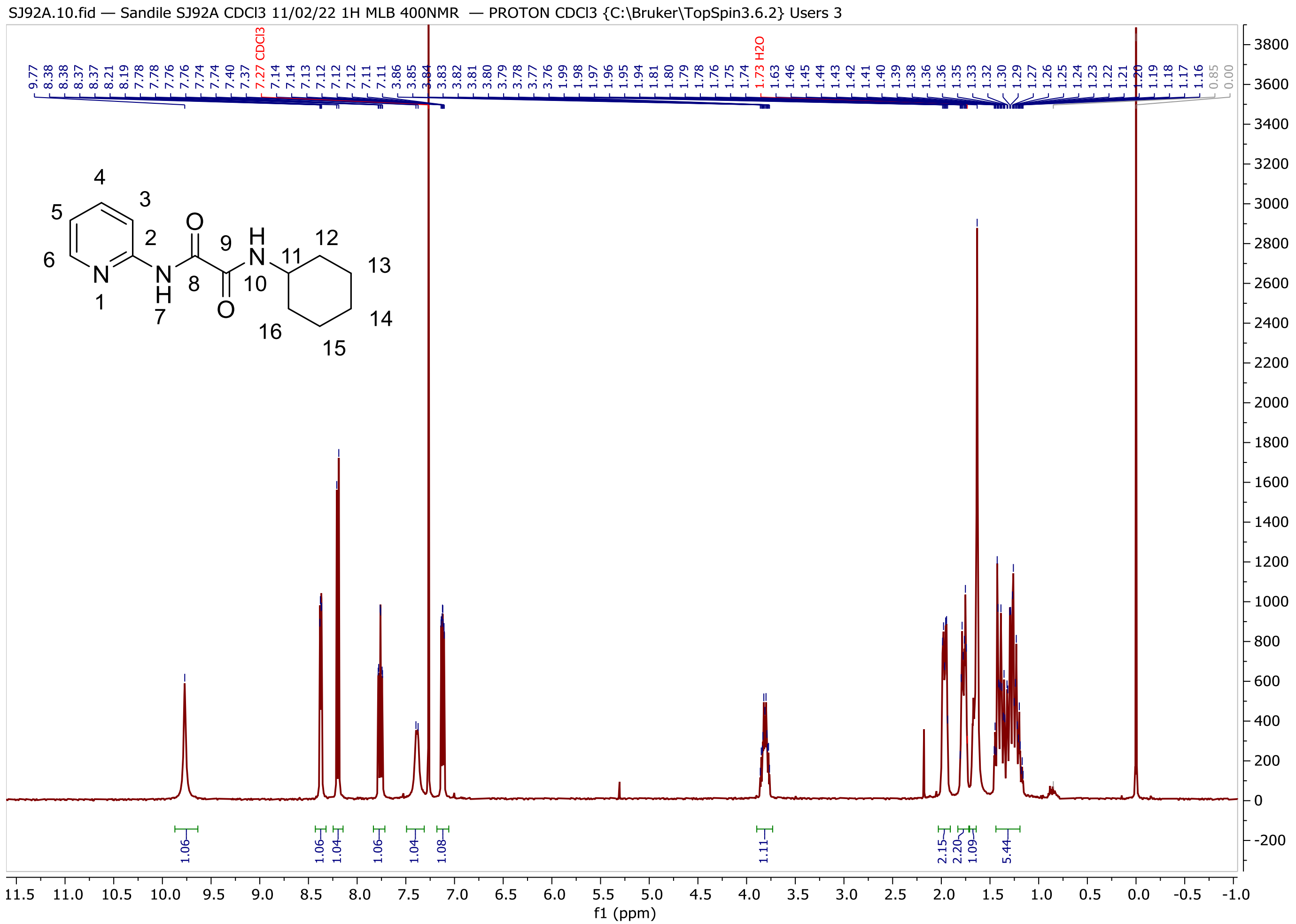

Fig. S30 ^{1}H NMR spectrum for compound **18d**

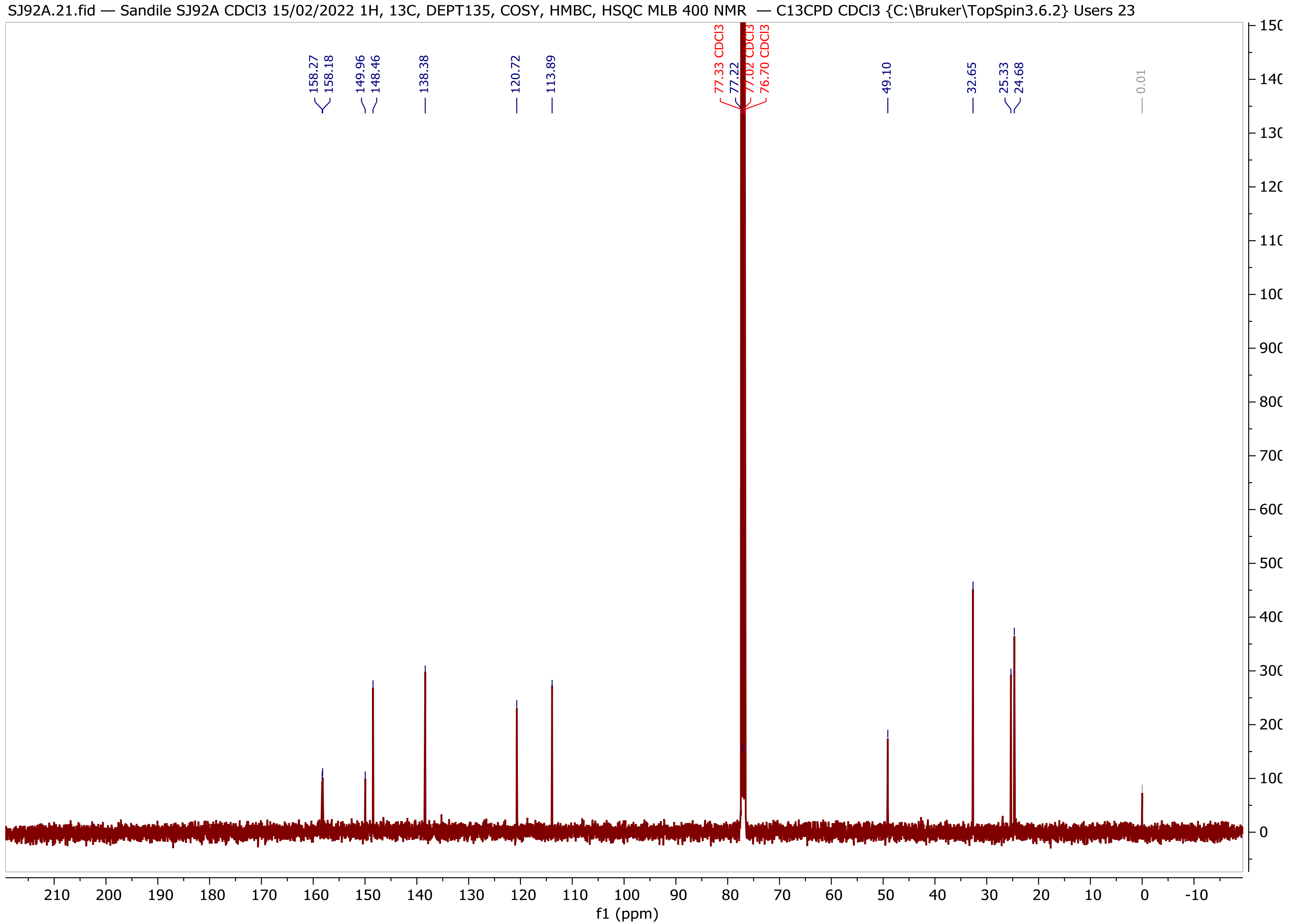

Fig. S31 ^{13}C NMR spectrum for compound **18d**

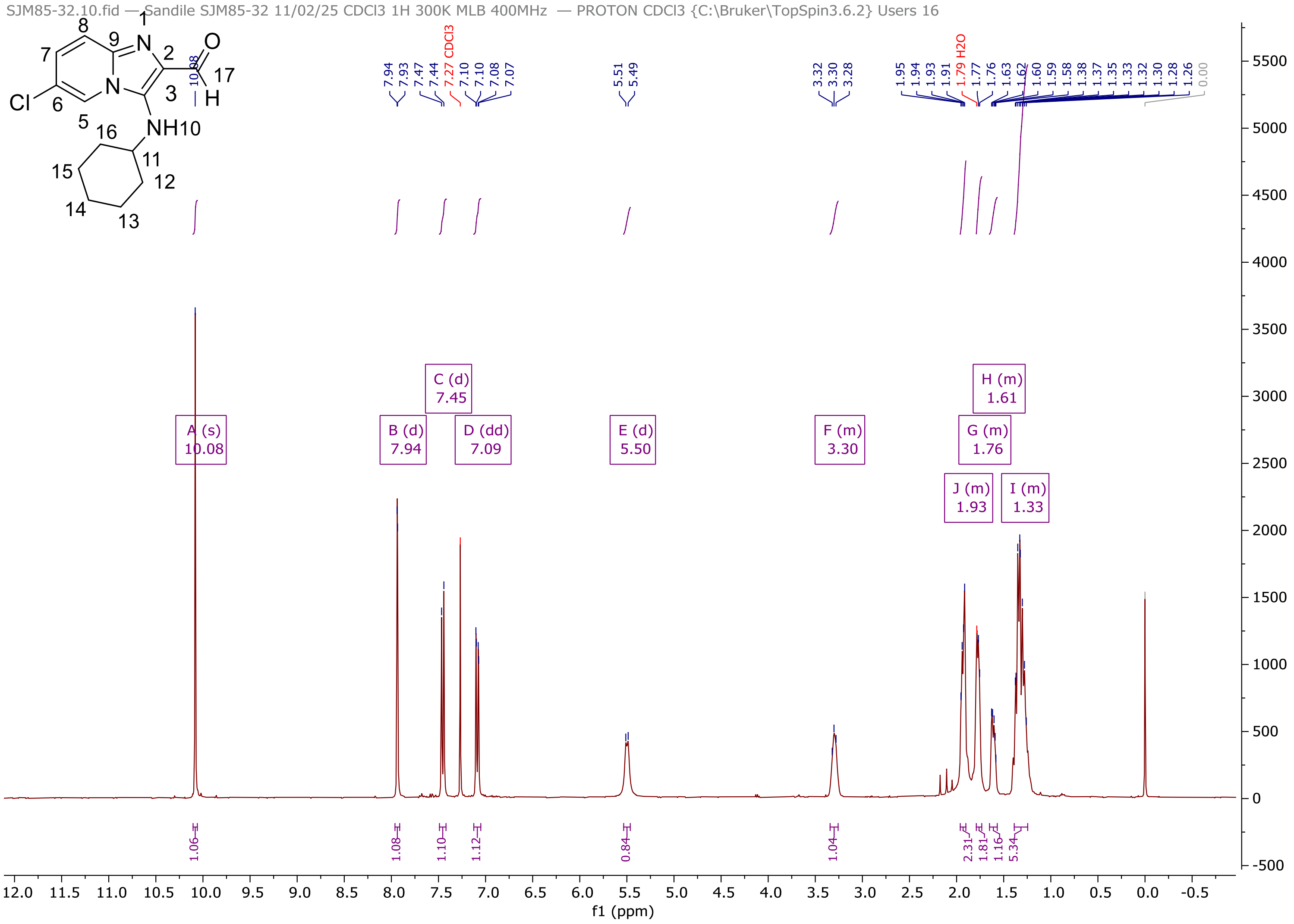

Fig. S32 ^{1}H NMR spectrum for compound **20a**

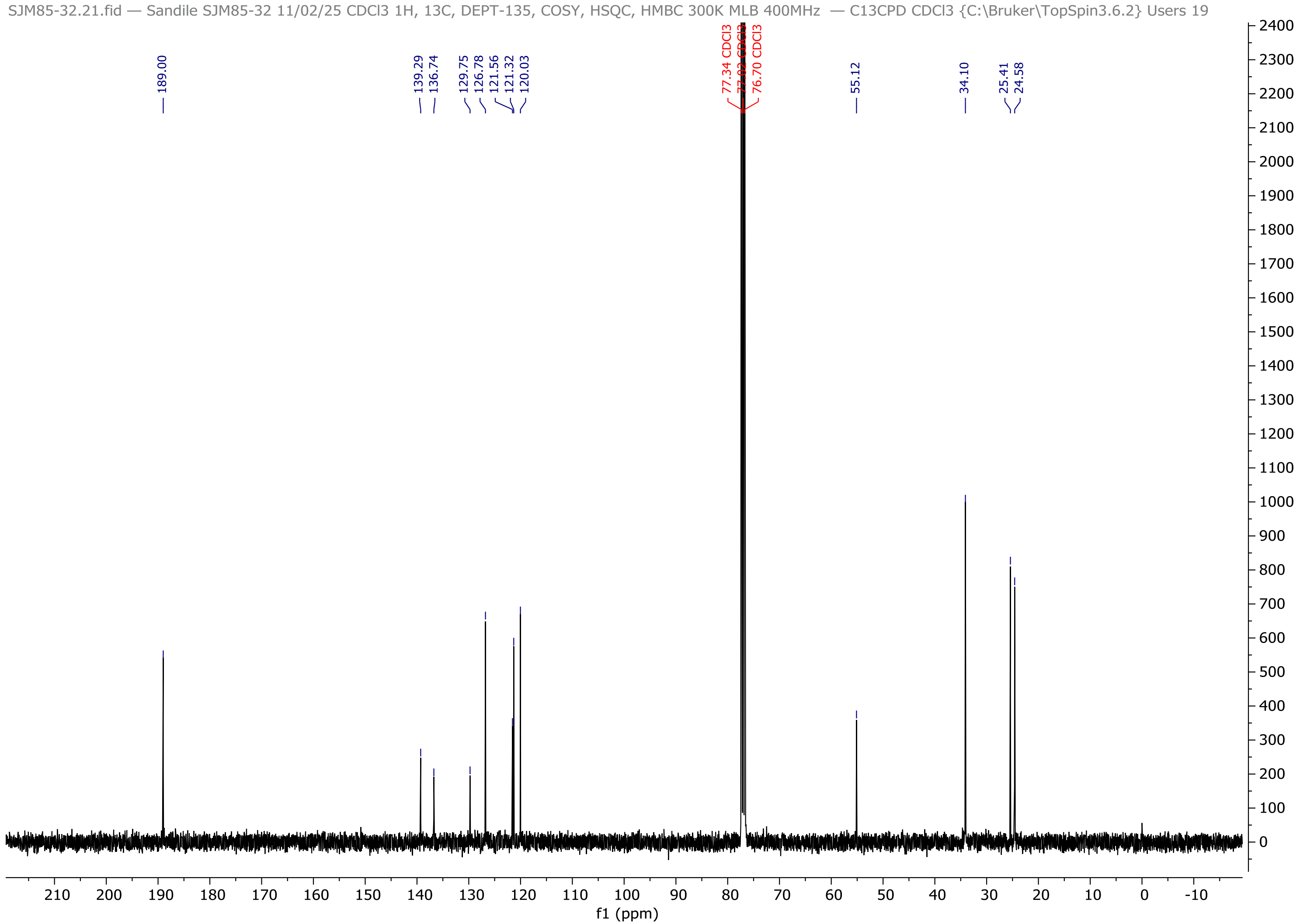

Fig. S33 ^{13}C NMR spectrum for compound **20a**

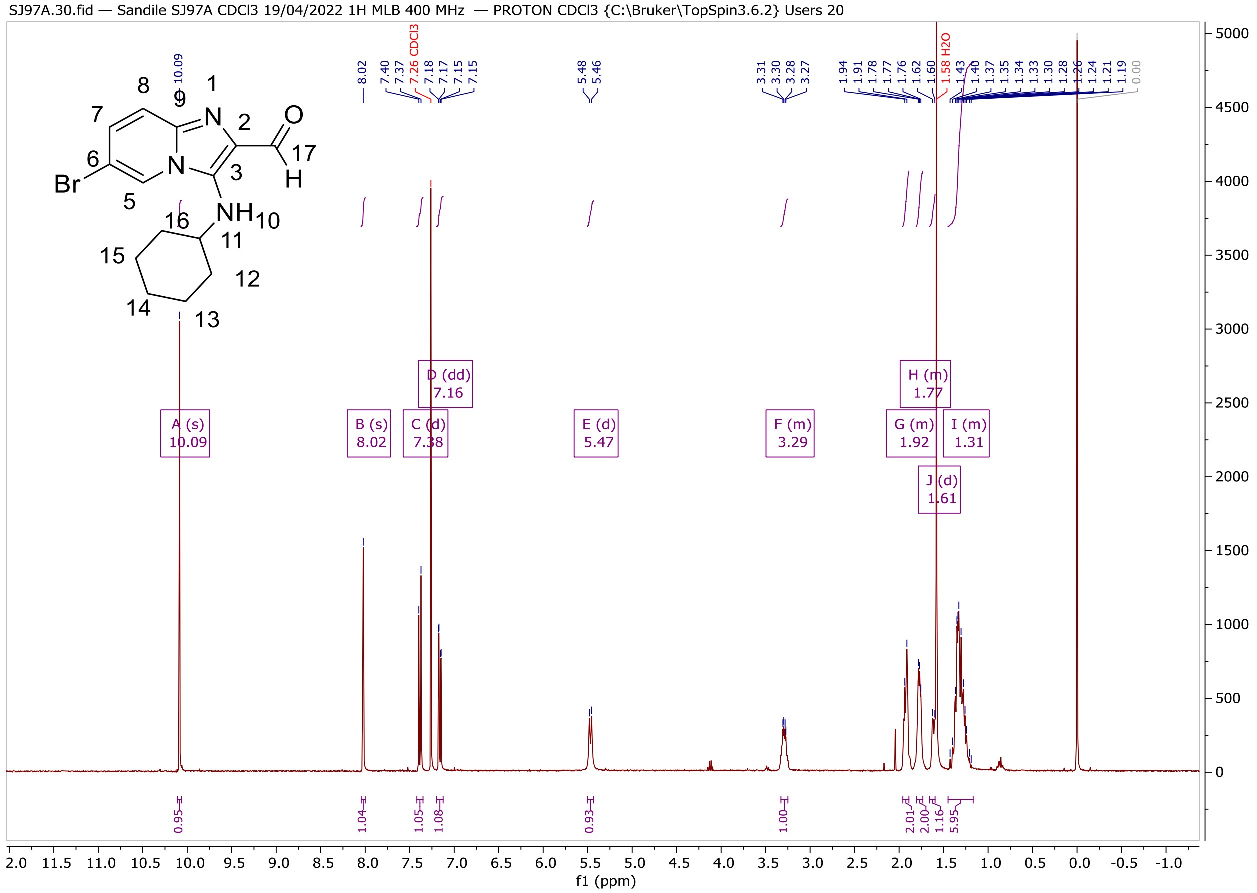

Fig. S34 ^{1}H NMR spectrum for compound **20b**

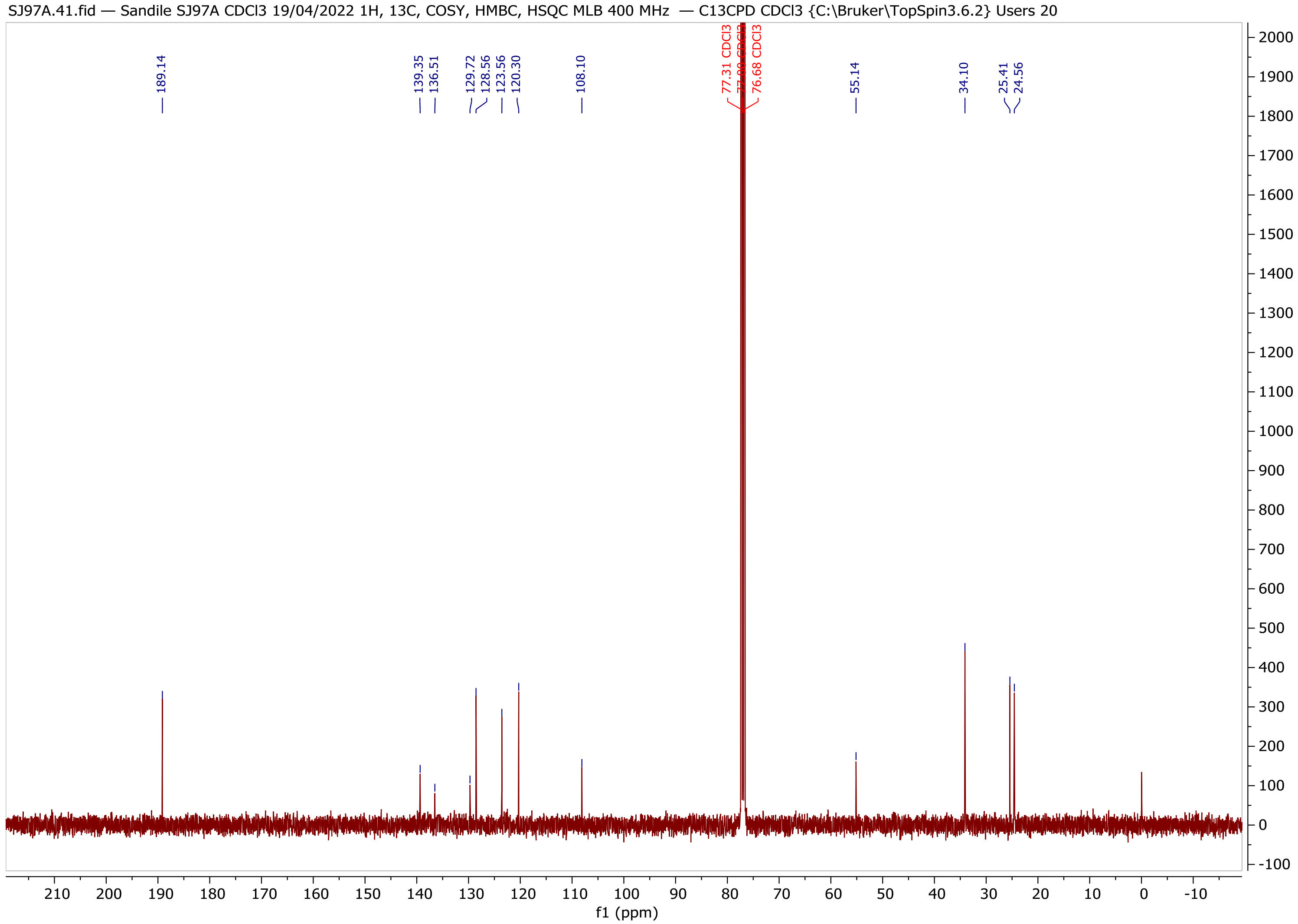

Fig. S35 ^{13}C NMR spectrum for compound **20b**

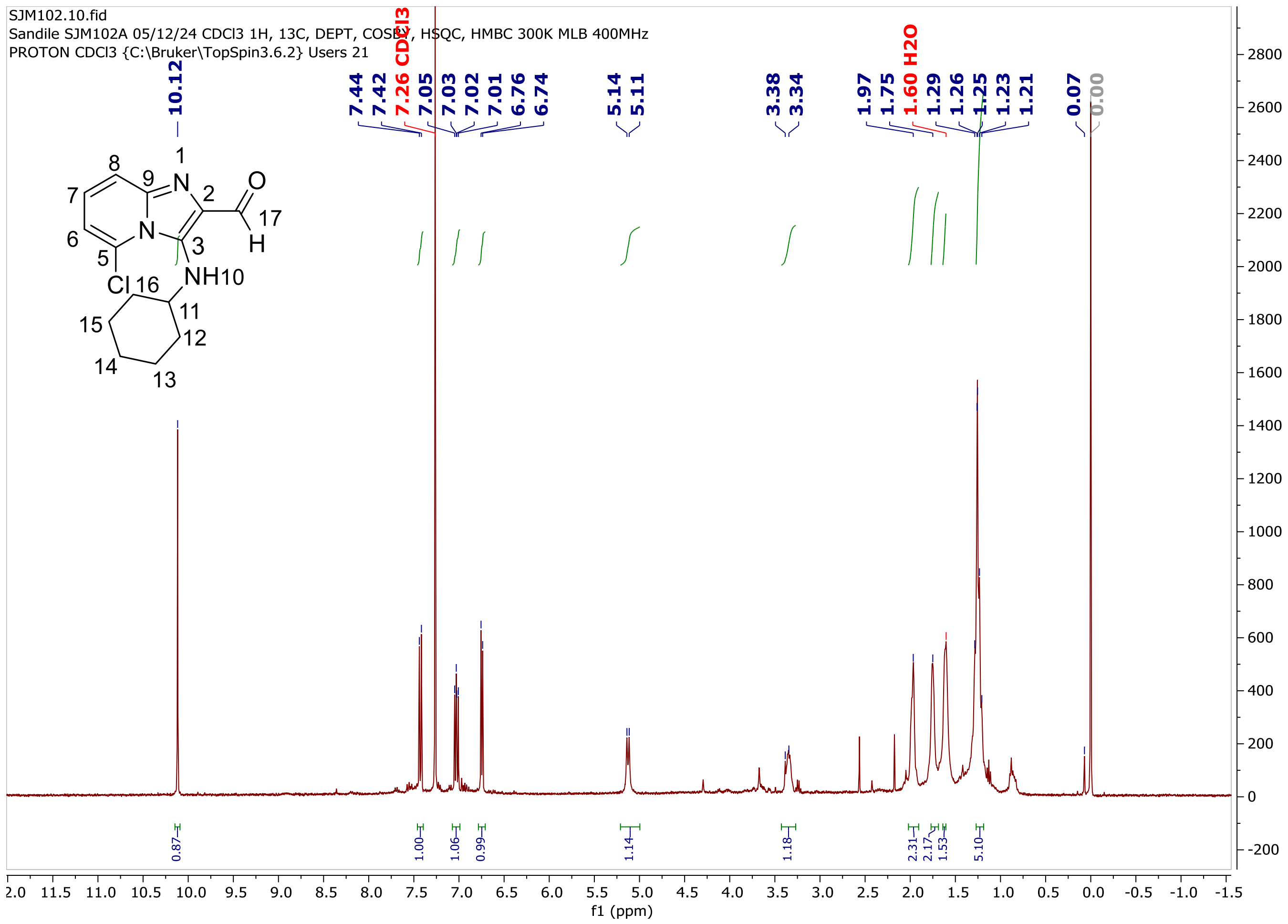

Fig. S36 ¹H NMR spectrum for compound **20c**

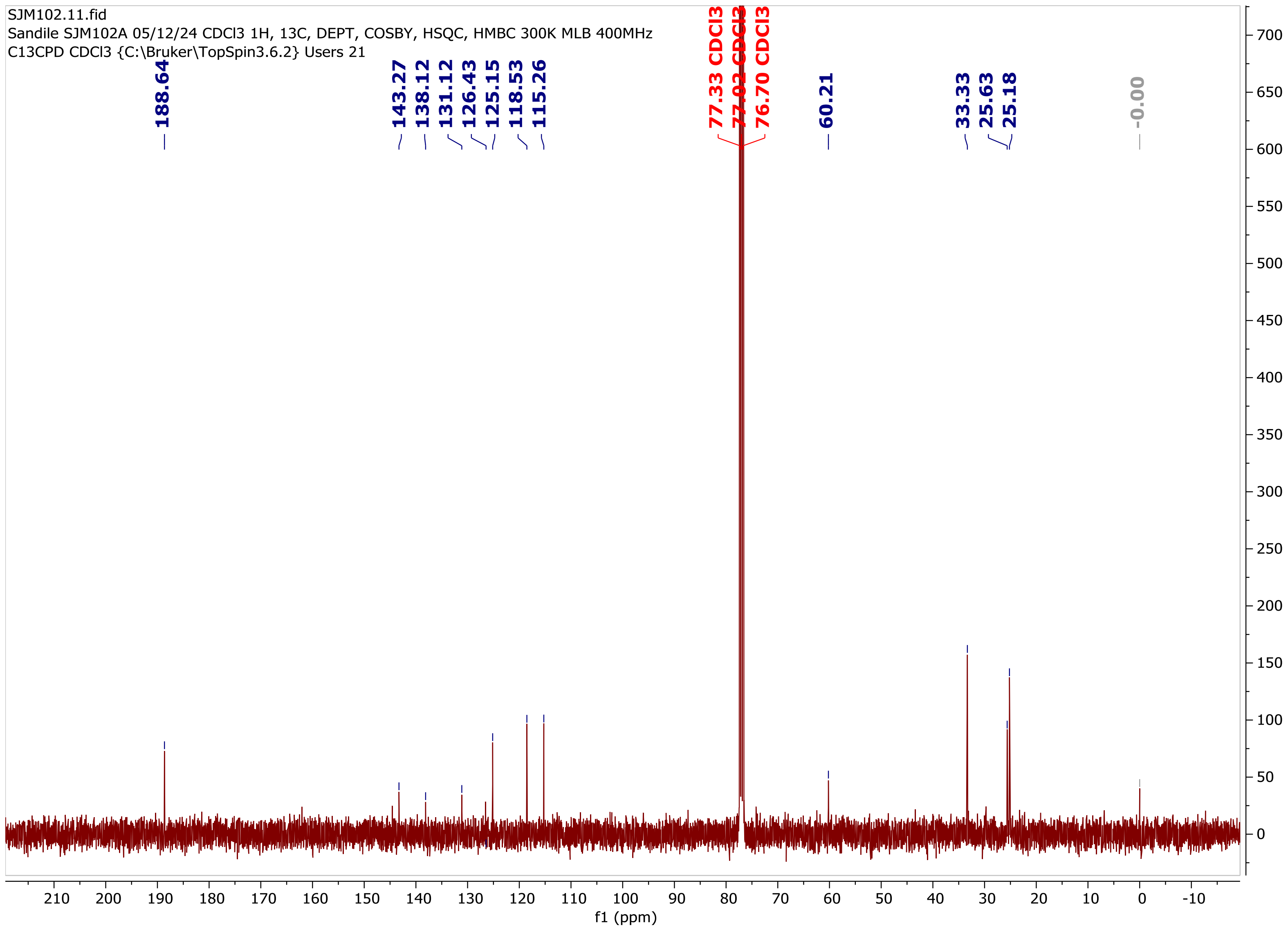

Fig. S37 ^{13}C NMR spectrum for compound **20c**

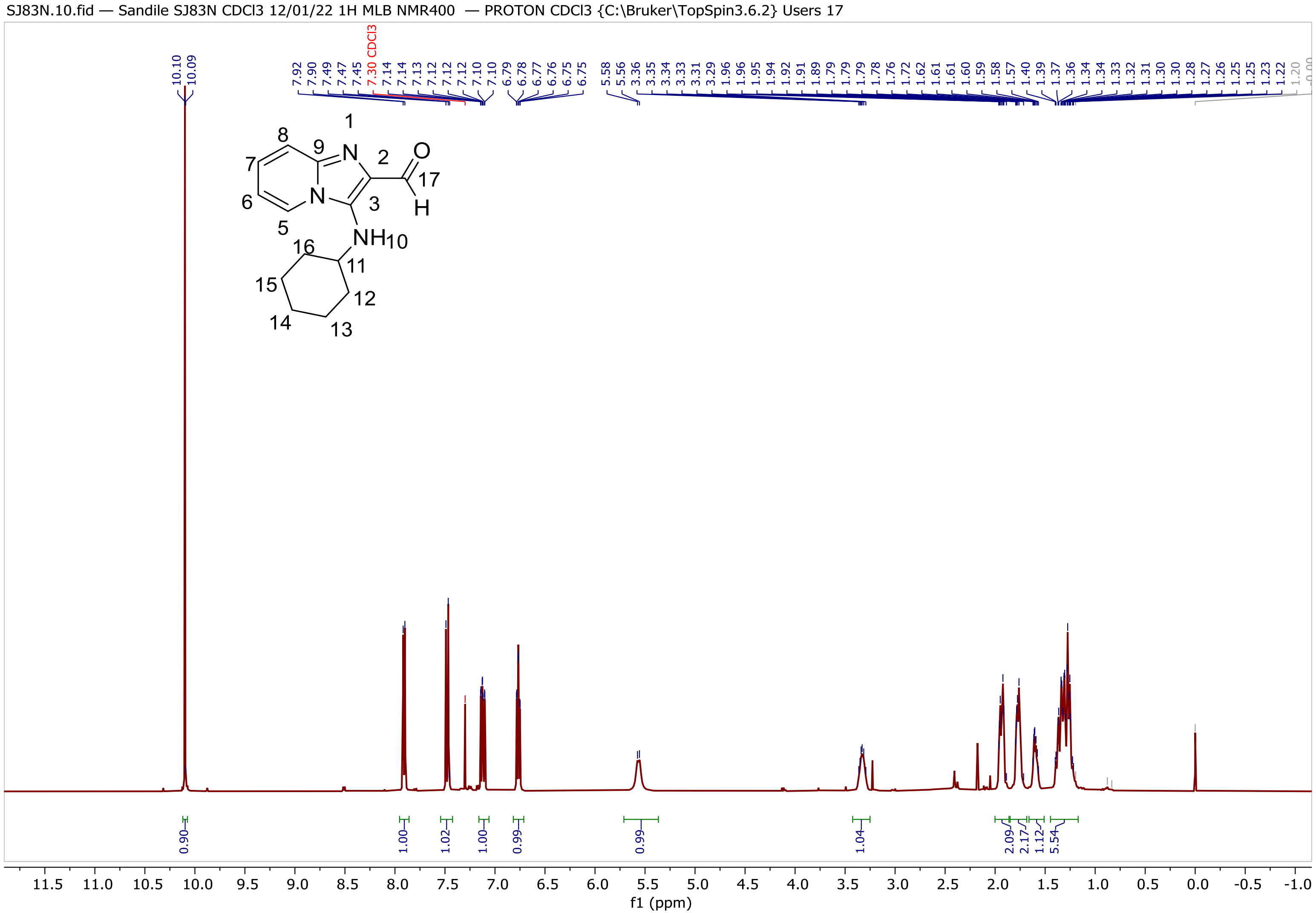

Fig. S38 ^{1}H NMR spectrum for compound **20d**

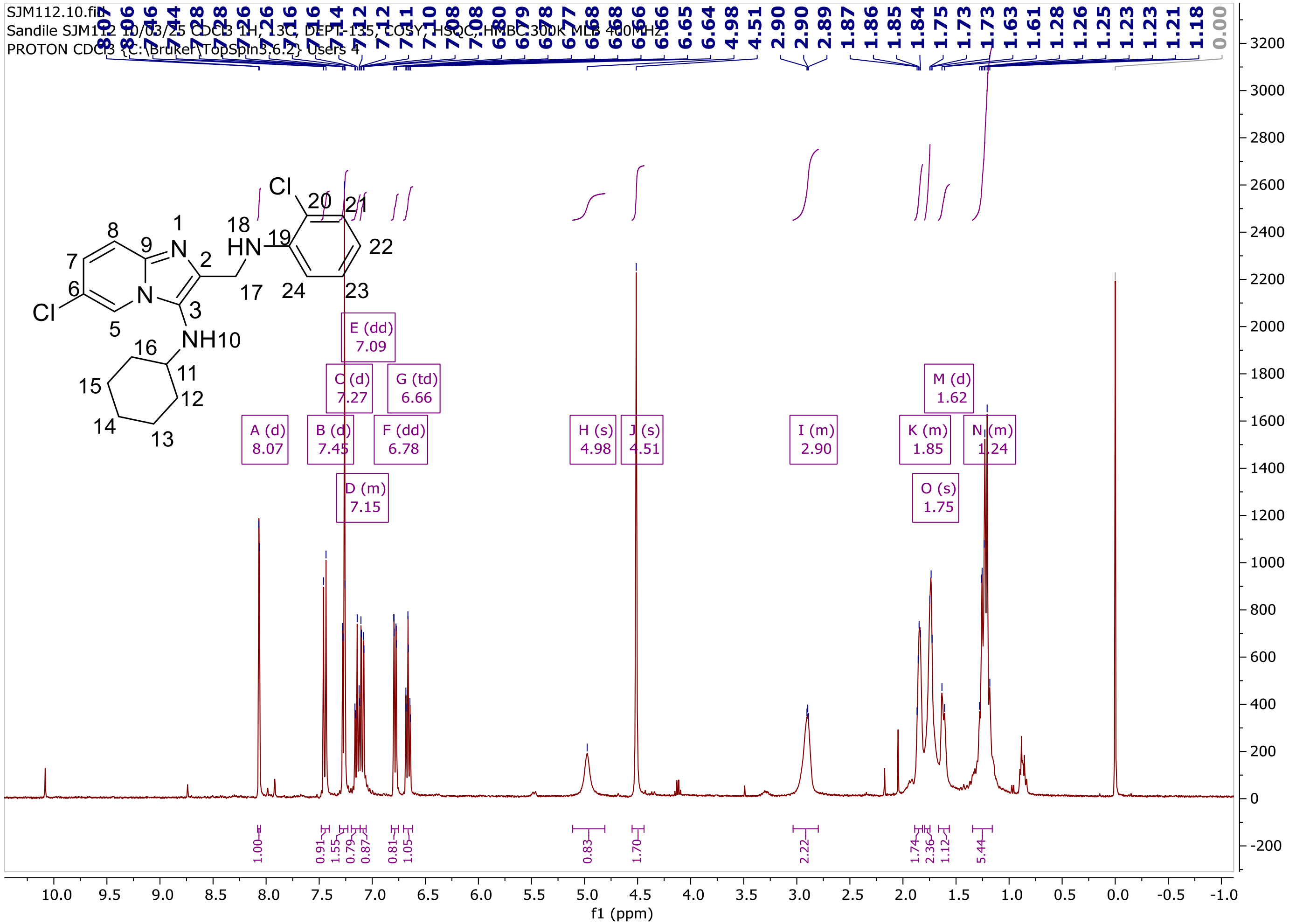

Fig. S39 ^{1}H NMR spectrum for compound **8a**

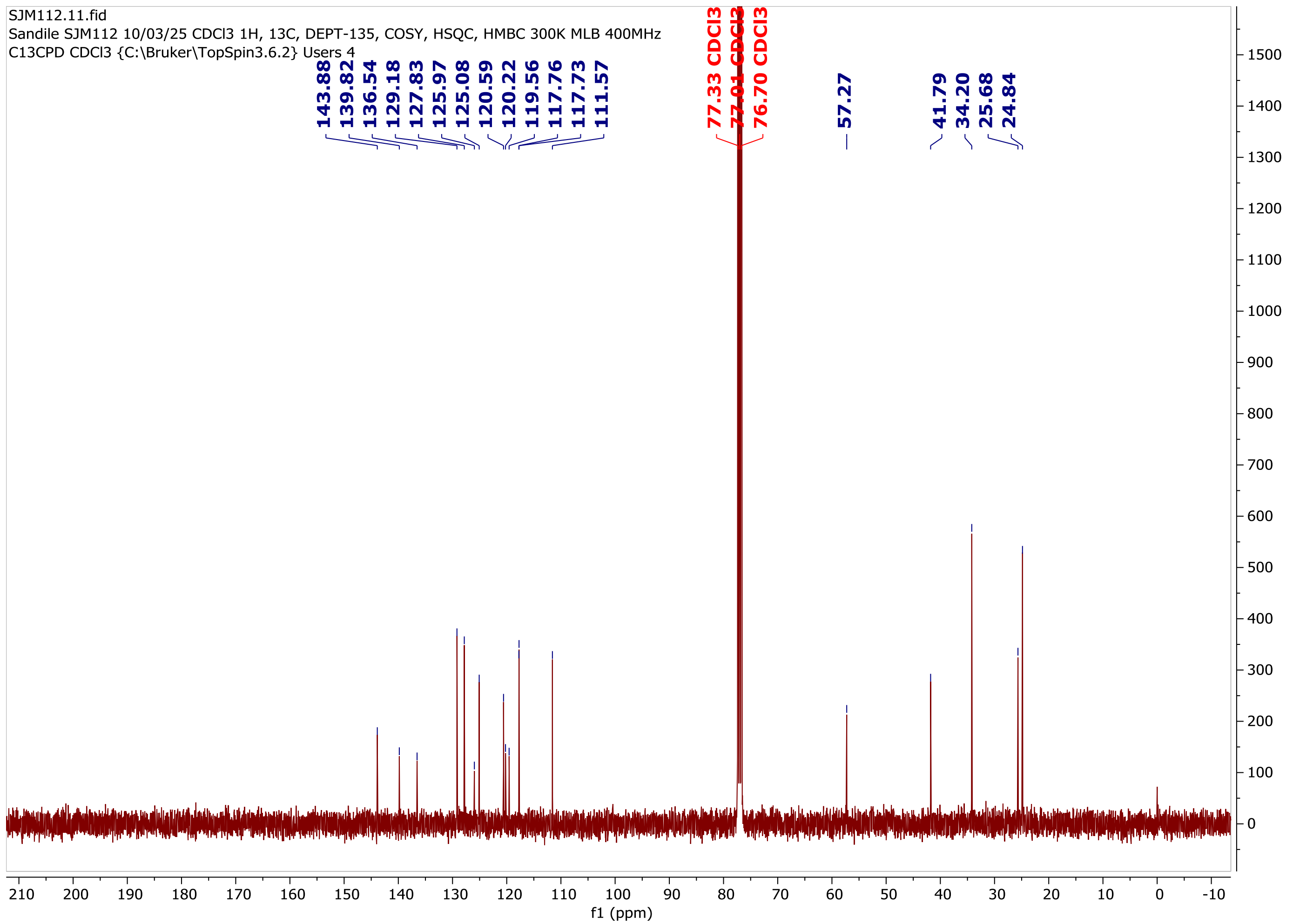

Fig. S40 ^{13}C NMR spectrum for compound **8a**

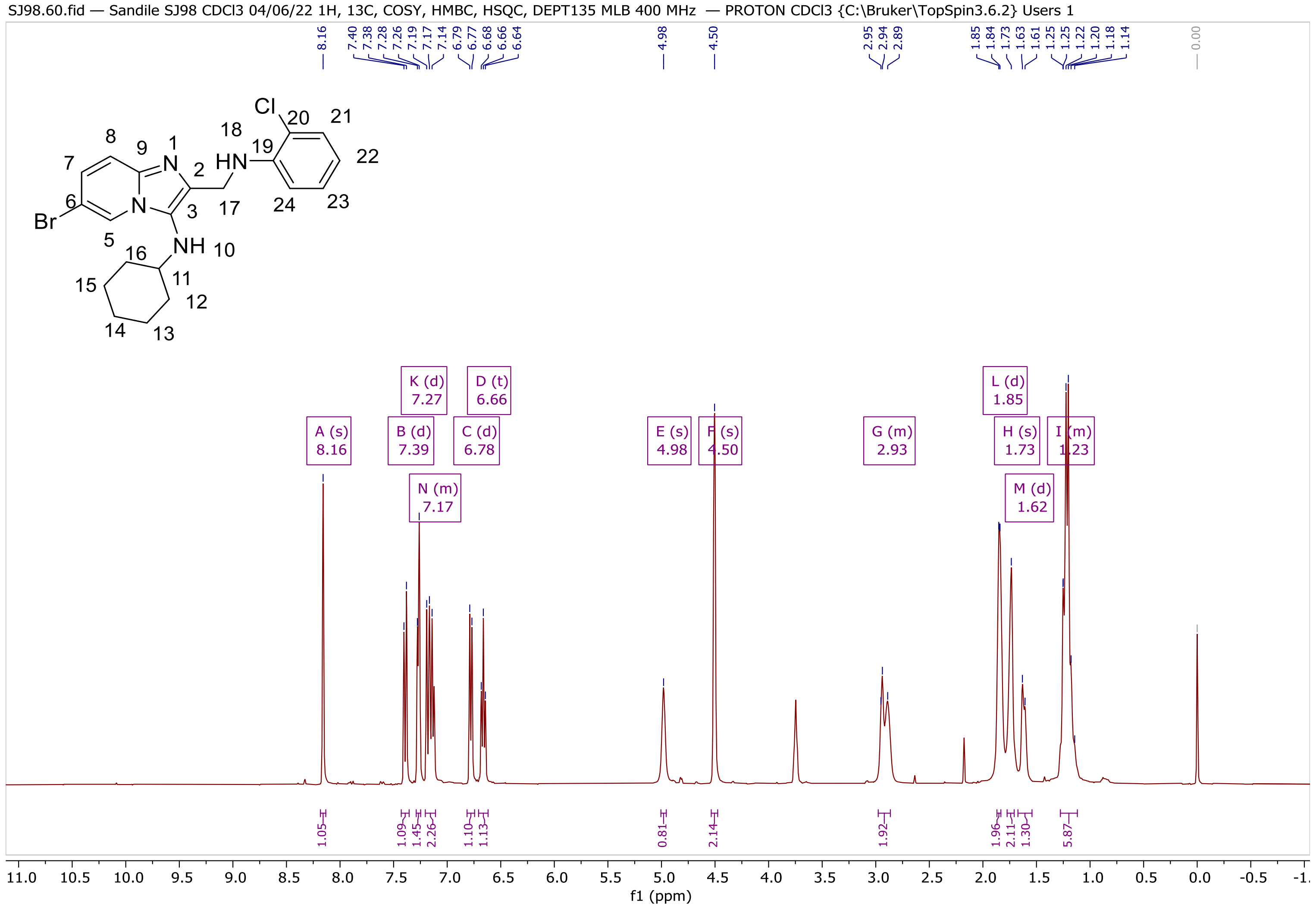

Fig. S41 ^{1}H NMR spectrum for compound **8b**

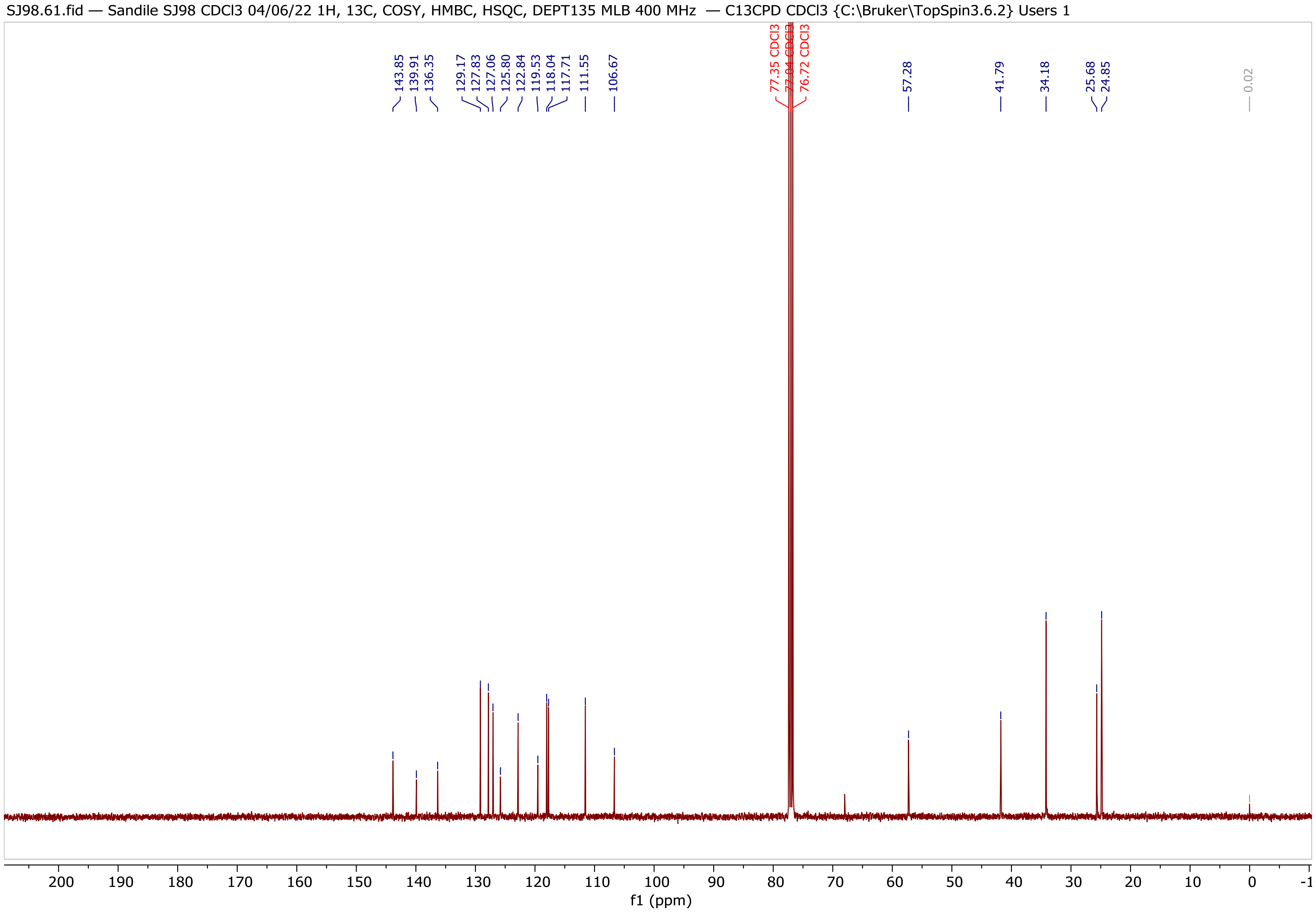

Fig. S42 ^{13}C NMR spectrum for compound **8b**

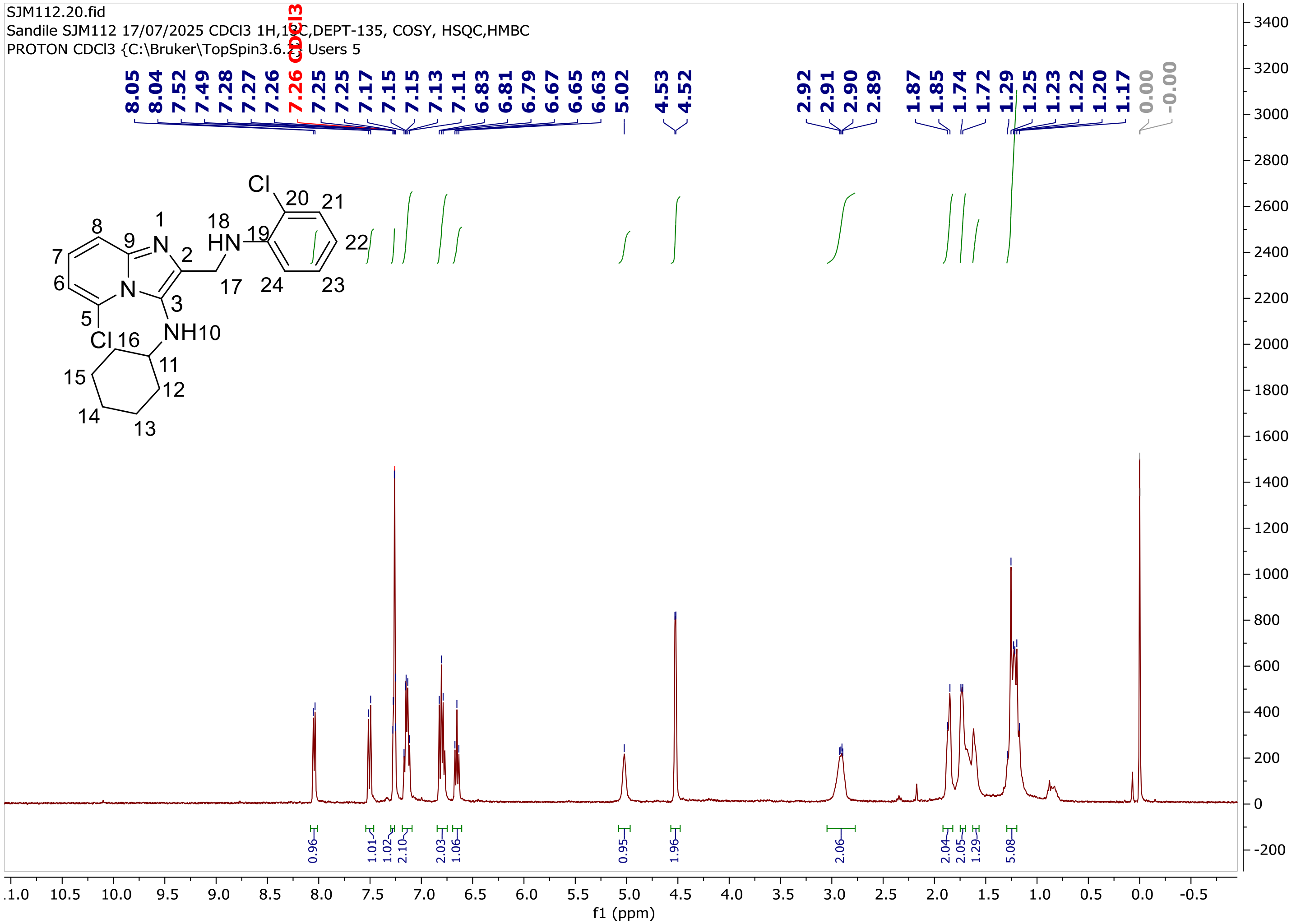

Fig. S43 ^{1}H NMR spectrum for compound **8c**

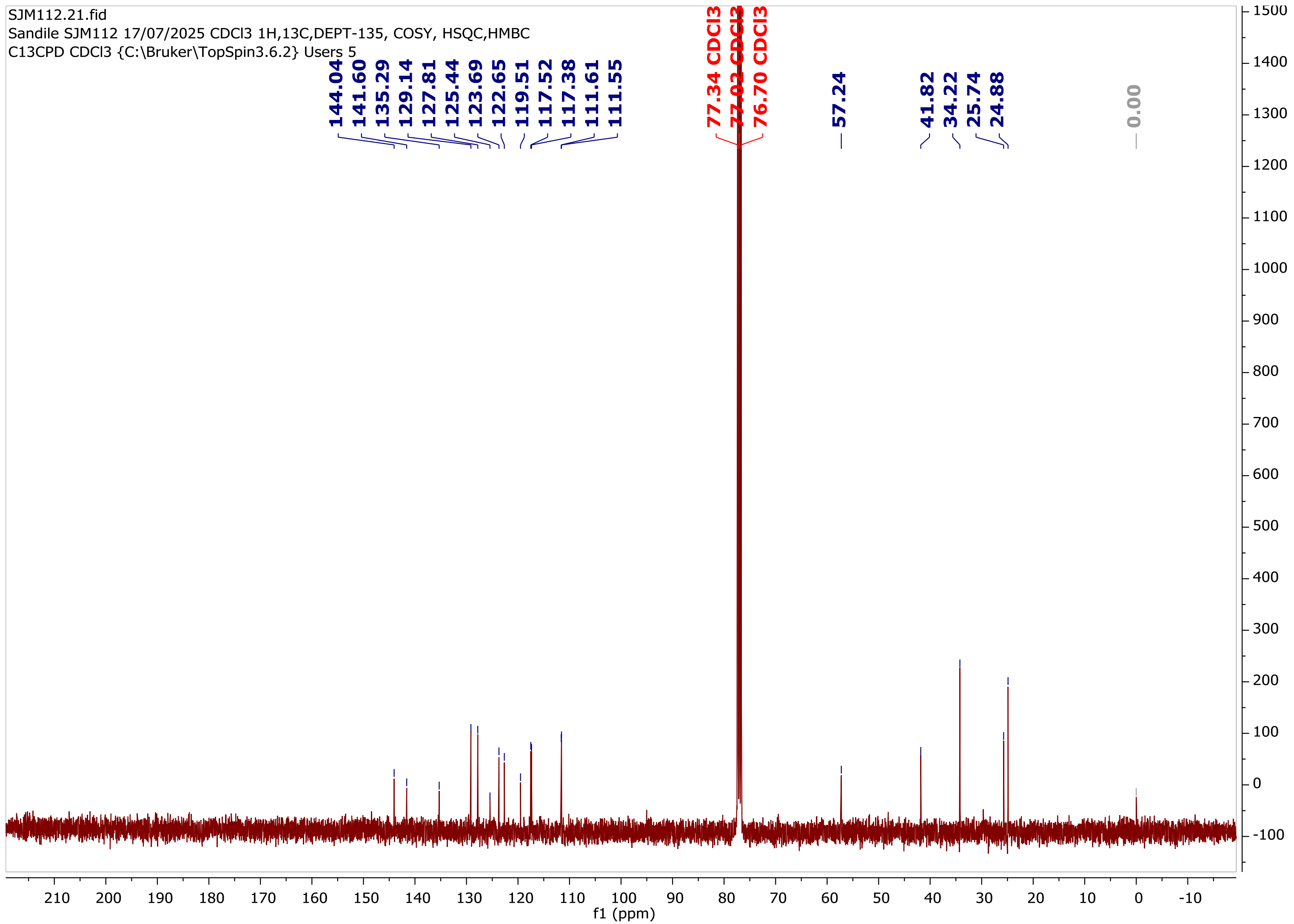

Fig. S44 ^{13}C NMR for compound **8c**

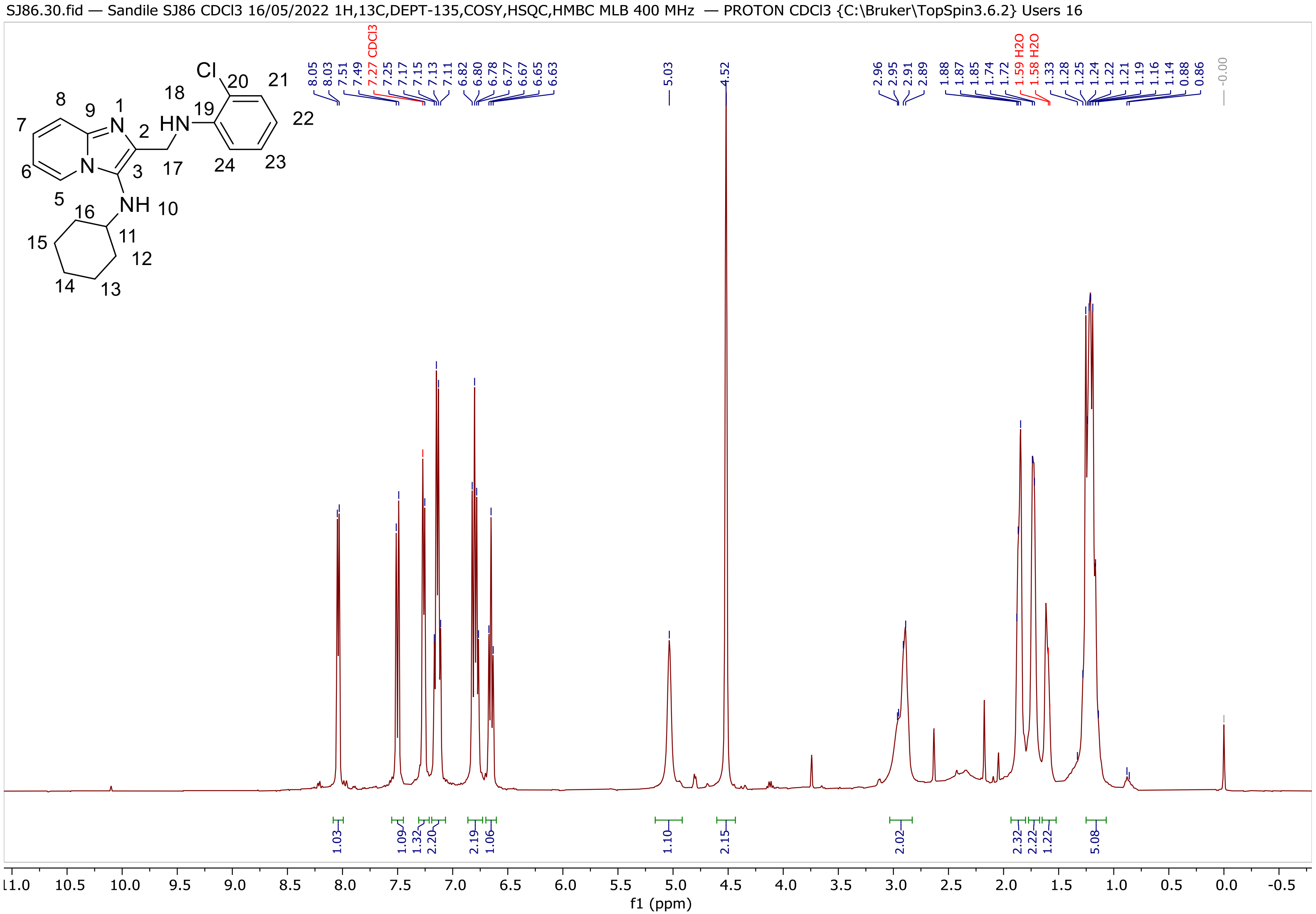

Fig. S45 ^{1}H NMR spectrum for compound **8d**

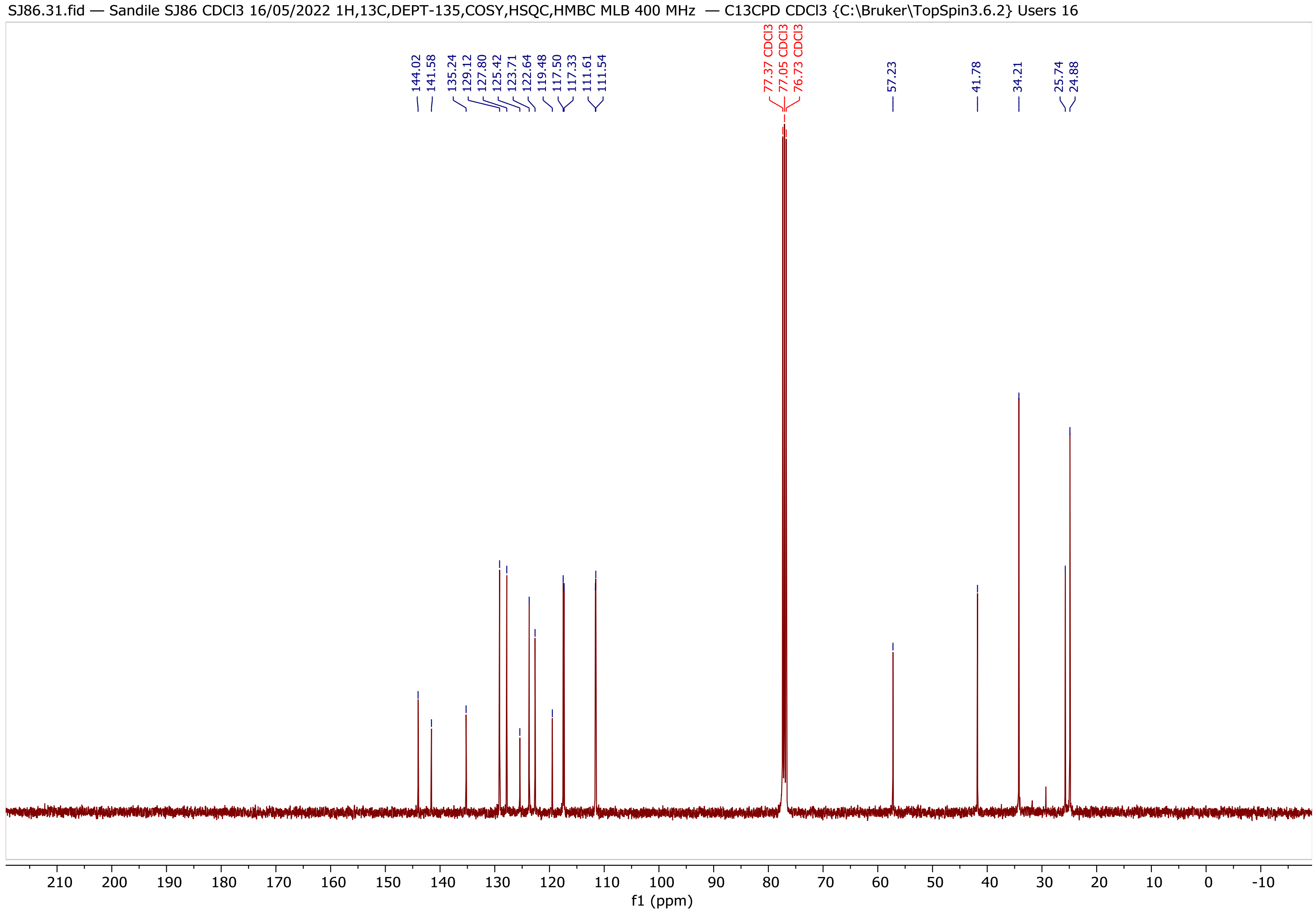

Fig. S46 ^{13}C NMR spectrum for compound **8d**

Crystal data collection and refinement

Intensity data for compound **18a** were collected on a Bruker D8 Venture Bio PHOTON III 28 pixel array area detector (208 × 128 mm 2) diffractometer with a Mo Kα IμS DIAMOND source (50 kV, 1.4 mA) at −100 °C. The collection method involved ω- and φ-scans, and 1536 × 1024 bit data frames. The unit cell and full data set were collected using APEX4[1], SAINT was used to integrate the data, and SADABS was used to make empirical absorption corrections and scale the data. Space group assignments were made using XPREP on all compounds. Using OLEX2[2], the crystal structures were solved with the ShelXT[3] structure solution program using Intrinsic Phasing and refined with the ShelXL[4] refinement package using Least Squares minimization. Non-hydrogen atoms were first refined isotropically followed by anisotropic refinement by full matrix least-squares calculations based on F_2.

Crystal data for $C_{13}H_{16}ClN_3O_2$ (M =281.74 g/mol): monoclinic, space group $P2_1$ (no. 4), a = 5.1405(2) Å, b = 6.5148(2) Å, c = 19.9758(8) Å, β = 97.171(2)°, V = 663.74(4) Å^3, Z = 2, T = 173.00 K, μ(MoKα) = 0.290 mm^{-1}, $Dcalc$ = 1.410 g/cm^3, 5327 reflections measured (6.166° ≤ 2Θ ≤ 55.986°), 2922 unique (R_{int} = 0.0723, R_{sigma} = 0.1080) which were used in all calculations. The final R_1 was 0.0546 (I > 2σ(I)) and wR_2 was 0.1060 (all data). CCDC deposition number: 2533649.

[1] APEX4, version 2021.4-1 data collection software which includes SAINT version 2028.2040B, SADABS-2016/2022 and XPREP version 2014/2022. Bruker AXS Inc., Madison, Wisconsin, USA.
[2] Dolomanov, O.V., Bourhis, L.J., Gildea, R.J, Howard, J.A.K. & Puschmann, H, *J. Appl. Cryst.*, **2009**, 42, 339-341.
[3] Sheldrick, G.M., *Acta Cryst.*, **2015**, A71, 3-8.
[4] Sheldrick, G.M., *Acta Cryst.*, **2015**, C71, 3-8.

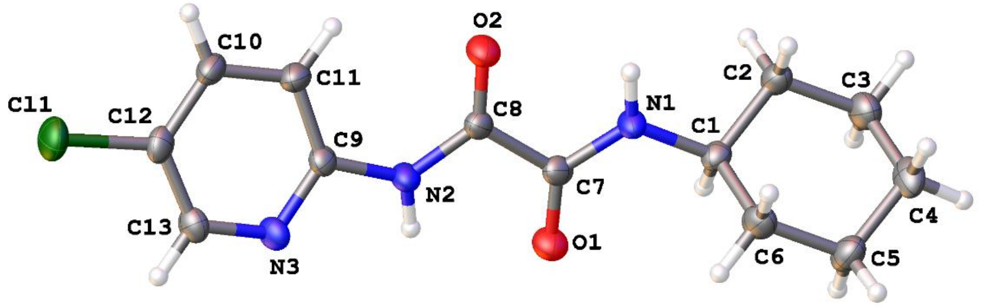

Fig. S47 Single crystal X-ray structure of compound **18a**. ORTEP diagram drawn at 50% probability level.

Table 1 Crystal data and structure refinement for **18a**.

Identification code	22mbB_MLB_SJ93
Empirical formula	$C_{13}H_{16}ClN_3O_2$
Formula weight	281.74
Temperature/K	173.00
Crystal system	monoclinic
Space group	$P2_1$
a/Å	5.1405(2)
b/Å	6.5148(2)
c/Å	19.9758(8)
α/°	90
β/°	97.171(2)
γ/°	90
Volume/Å^3	663.74(4)
Z	2
ρ_{calc}g/cm^3	1.410
μ/mm^{-1}	0.290
F(000)	296.0
Crystal size/mm^3	0.265 × 0.226 × 0.186
Radiation	MoKα (λ = 0.71073)
2Θ range for data collection/°	6.166 to 55.986
Index ranges	$-6 \le h \le 6$, $-8 \le k \le 8$, $-26 \le l \le 26$
Reflections collected	5327
Independent reflections	2922 [R_{int} = 0.0723, R_{sigma} = 0.1080]
Data/restraints/parameters	2922/1/180
Goodness-of-fit on F^2	1.013
Final R indexes [I>=2σ (I)]	R_1 = 0.0546, wR_2 = 0.0910
Final R indexes [all data]	R_1 = 0.1050, wR_2 = 0.1060
Largest diff. peak/hole / e Å^{-3}	0.26/-0.27
Flack parameter	-0.05(8)

Table S2 Fractional atomic coordinates (×10[4]) and equivalent isotropic displacement parameters (Å[2]×10[3]) for **18a**. U_{eq} is defined as 1/3 of the trace of the orthogonalised U_{IJ} tensor.

Atom	*x*	*y*	*z*	U(eq)
Cl^1	5908(2)	-3344.8(19)	9574.2(7)	41.4(4)
O^1	4050(5)	7223(5)	7132.4(15)	31.6(8)
O^2	9829(5)	5411(5)	8065.7(15)	31.0(8)
N^1	8259(7)	8354(6)	7141.0(19)	24.9(9)
N^2	5641(7)	4141(6)	7977.2(18)	25.3(9)
N^3	3899(7)	1095(5)	8265.5(19)	26.9(9)
C^1	7646(8)	10012(7)	6651(2)	24.4(10)
C^2	9756(8)	11622(8)	6747(2)	29.4(10)
C^3	9132(9)	13384(8)	6246(2)	36.8(13)
C^4	8770(9)	12594(8)	5525(2)	35.6(12)
C^5	6722(10)	10919(7)	5426(2)	36.8(12)
C^6	7310(10)	9179(8)	5936(2)	34.3(12)
C^7	6409(8)	7128(7)	7335(2)	23.6(10)
C^8	7514(8)	5481(7)	7836(2)	22.9(10)
C^9	5942(8)	2355(7)	8376(2)	23.2(10)
C^{10}	8111(8)	1998(7)	8841(2)	28.5(11)
C^{11}	8157(9)	204(7)	9215(2)	29.2(11)
C^{12}	6031(8)	-1119(7)	9103(2)	26.7(10)
C^{13}	3969(9)	-637(7)	8627(2)	28.0(11)

Table S3 Anisotropic displacement parameters (Å[2]×10[3]) for **18a**. The anisotropic displacement factor exponent takes the form: $-2\pi^2[h^2a^{*2}U_{11}+2hka^*b^*U_{12}+\ldots]$.

Atom	U_{11}	U_{22}	U_{33}	U_{23}	U_{13}	U_{12}
Cl^1	48.5(7)	34.3(7)	41.5(7)	15.7(6)	6.1(6)	-0.6(7)
O^1	22.5(16)	35(2)	37(2)	4.8(15)	3.2(14)	-0.1(15)
O^2	21.6(17)	32.6(19)	38(2)	7.8(16)	1.6(14)	-1.8(15)
N^1	16.2(19)	26(2)	32(2)	9.4(17)	1.5(17)	3.1(19)
N^2	19(2)	28(2)	28(2)	6.1(18)	-0.2(17)	-2.8(19)
N^3	25(2)	23(2)	33(2)	3.3(16)	1.7(16)	-1.9(17)
C^1	21(2)	28(3)	23(3)	4.1(19)	0.8(19)	3(2)
C^2	34(2)	28(2)	25(2)	1(2)	-1.9(19)	-5(2)
C^3	40(3)	28(3)	41(3)	7(2)	1(2)	-5(2)
C^4	31(3)	33(3)	44(3)	14(2)	6(2)	5(2)
C^5	45(3)	36(3)	27(3)	5(2)	-4(2)	-1(2)
C^6	40(3)	28(3)	33(3)	3(2)	-1(2)	-7(2)
C^7	22(2)	27(3)	22(2)	-1.3(19)	5.8(18)	-2(2)
C^8	23(2)	25(3)	21(2)	1(2)	2.4(19)	-3(2)
C^9	21(2)	26(2)	24(2)	4(2)	6.3(18)	0(2)
C^{10}	25(2)	32(3)	27(3)	5(2)	-0.1(19)	-3(2)
C^{11}	25(2)	37(3)	25(3)	7(2)	1.3(19)	-1(2)

Table S3 Anisotropic displacement parameters ($Å^2 \times 10^3$) for **18a**. The anisotropic displacement factor exponent takes the form: $-2\pi^2[h^2a^{*2}U_{11}+2hka^*b^*U_{12}+\ldots]$.

Atom	U_{11}	U_{22}	U_{33}	U_{23}	U_{13}	U_{12}
C^{12}	32(2)	27(3)	24(3)	5(2)	10(2)	3(2)
C^{13}	29(2)	26(3)	29(3)	2(2)	7(2)	-2(2)

Table S4 Bond lengths for **18a**.

Atom	Atom	Length/Å	Atom	Atom	Length/Å
Cl^1	C^{12}	1.735(5)	C^1	C^6	1.517(6)
O^1	C^7	1.231(5)	C^2	C^3	1.531(6)
O^2	C^8	1.221(5)	C^3	C^4	1.517(6)
N^1	C^1	1.466(5)	C^4	C^5	1.512(6)
N^1	C^7	1.336(5)	C^5	C^6	1.530(6)
N^2	C^8	1.355(5)	C^7	C^8	1.528(6)
N^2	C^9	1.407(6)	C^9	C^{10}	1.378(6)
N^3	C^9	1.329(5)	C^{10}	C^{11}	1.387(6)
N^3	C^{13}	1.338(5)	C^{11}	C^{12}	1.388(6)
C^1	C^2	1.504(6)	C^{12}	C^{13}	1.368(6)

Table S5 Bond angles for **18a**.

Atom	Atom	Atom	Angle/°	Atom	Atom	Atom	Angle/°
C^7	N^1	C^1	122.2(4)	N^1	C^7	C^8	112.9(3)
C^8	N^2	C^9	128.3(4)	O^2	C^8	N^2	125.6(4)
C^9	N^3	C^{13}	117.6(4)	O^2	C^8	C^7	122.5(4)
N^1	C^1	C^2	109.7(3)	N^2	C^8	C^7	111.9(3)
N^1	C^1	C^6	110.8(4)	N^3	C^9	N^2	112.8(4)
C^2	C^1	C^6	111.4(4)	N^3	C^9	C^{10}	124.3(4)
C^1	C^2	C^3	110.5(3)	C^{10}	C^9	N^2	122.9(4)
C^4	C^3	C^2	110.9(4)	C^9	C^{10}	C^{11}	117.6(4)
C^5	C^4	C^3	111.8(4)	C^{10}	C^{11}	C^{12}	118.4(4)
C^4	C^5	C^6	111.8(4)	C^{11}	C^{12}	Cl^1	120.9(3)
C^1	C^6	C^5	110.6(4)	C^{13}	C^{12}	Cl^1	119.4(4)
O^1	C^7	N^1	125.6(4)	C^{13}	C^{12}	C^{11}	119.7(4)
O^1	C^7	C^8	121.4(4)	N^3	C^{13}	C^{12}	122.3(4)

Table S6 Torsion angles for **18a**.

A	B	C	D	Angle/°	A	B	C	D	Angle/°
Cl^1	C^{12}	C^{13}	N^3	177.4(3)	C^4	C^5	C^6	C^1	54.2(5)
O^1	C^7	C^8	O^2	-176.8(4)	C^6	C^1	C^2	C^3	57.8(5)
O^1	C^7	C^8	N^2	4.1(5)	C^7	N^1	C^1	C^2	156.1(4)
N^1	C^1	C^2	C^3	-179.2(4)	C^7	N^1	C^1	C^6	-80.4(5)
N^1	C^1	C^6	C^5	-178.9(4)	C^8	N^2	C^9	N^3	-162.5(4)
N^1	C^7	C^8	O^2	4.8(6)	C^8	N^2	C^9	C^{10}	18.9(7)
N^1	C^7	C^8	N^2	-174.2(4)	C^9	N^2	C^8	O^2	-3.8(8)
N^2	C^9	C^{10}	C^{11}	178.5(4)	C^9	N^2	C^8	C^7	175.2(4)
N^3	C^9	C^{10}	C^{11}	0.1(7)	C^9	N^3	C^{13}	C^{12}	0.8(6)
C^1	N^1	C^7	O^1	0.4(7)	C^9	C^{10}	C^{11}	C^{12}	-0.1(6)
C^1	N^1	C^7	C^8	178.6(4)	C^{10}	C^{11}	C^{12}	Cl^1	-177.7(3)
C^1	C^2	C^3	C^4	-56.4(5)	C^{10}	C^{11}	C^{12}	C^{13}	0.4(7)
C^2	C^1	C^6	C^5	-56.5(5)	C^{11}	C^{12}	C^{13}	N^3	-0.8(7)
C^2	C^3	C^4	C^5	54.7(5)	C^{13}	N^3	C^9	N^2	-179.0(4)
C^3	C^4	C^5	C^6	-53.8(5)	C^{13}	N^3	C^9	C^{10}	-0.5(6)

Table S7 Hydrogen atom coordinates (Å×10^4) and isotropic displacement parameters (Å^2×10^3) for **18a.**

Atom	*x*	*y*	*z*	U(eq)
H^{1A}	5954.07	10657.02	6737.19	29
H^{2A}	11461.5	11000.65	6679.68	35
H^{2B}	9897.03	12160.62	7213.51	35
H^{3A}	7510.92	14086.52	6341.45	44
H^{3B}	10579.78	14395.74	6301.1	44
H^{4A}	8236.15	13744.68	5214.53	43
H^{4B}	10460.91	12054.26	5412.18	43
H^{5A}	6659	10359.13	4963.17	44
H^{5B}	4979.8	11509.11	5473.18	44
H^{6A}	5854.93	8173.39	5882.52	41
H^{6B}	8932.78	8461.15	5849.58	41
H^{10}	9523.05	2948.07	8902.09	34
H^{11}	9609.09	-111.5	9541.52	35
H^{13}	2537.58	-1565.38	8551.5	34
H^1	9810(80)	7960(60)	7270(20)	19(12)
H^2	4130(90)	4370(80)	7740(20)	47(16)
